# Protein Abundance Control by Non-coding Antisense Transcription

**DOI:** 10.1016/j.celrep.2016.05.043

**Published:** 2016-06-09

**Authors:** Florian Huber, Daria Bunina, Ishaan Gupta, Anton Khmelinskii, Matthias Meurer, Patrick Theer, Lars M. Steinmetz, Michael Knop

**Affiliations:** 1 Zentrum für Molekulare Biologie der Universität Heidelberg (ZMBH), University of Heidelberg, Im Neuenheimer Feld 282, 69120 Heidelberg, Germany; 2 Genome Biology Unit, European Molecular Biology Laboratory (EMBL), Meyerhofstraße 1, 69117 Heidelberg, Germany; 3 Deutsches Krebsforschungszentrum (DKFZ), Im Neuenheimer Feld 280, 69120 Heidelberg, Germany; 4 Stanford Genome Technology Center, Stanford University, Palo Alto, CA 94304, USA; 5 Department of Genetics, Stanford University School of Medicine, Stanford, CA 94305, USA

## Abstract

Stable unannotated transcripts (SUTs), some of which overlap protein-coding genes in antisense direction, are a class of non-coding RNAs. While case studies have reported important regulatory roles for several of such RNAs, their general impact on protein abundance regulation of the overlapping gene is not known. To test this, we employed seamless gene manipulation to repress antisense SUTs of 162 yeast genes by using a unidirectional transcriptional terminator and a GFP tag. We found that the mere presence of antisense SUTs was not sufficient to influence protein abundance, that observed effects of antisense SUTs correlated with sense transcript start site overlap, and that the effects were generally weak and led to reduced protein levels. Antisense regulated genes showed increased H3K4 di- and trimethylation and had slightly lower than expected noise levels. Our results suggest that the functionality of antisense RNAs has gene and condition-specific components.

## Highlights

• The impact of antisense transcription on protein levels is measured for 188 genes
• Antisense has mostly weak suppressive effects on ∼25% of the genes
• Regulation by antisense correlates with promoter overlap and H3K4 methylation
• Antisense-regulated genes have lower than expected noise levels

## Graphical Abstract

**Figure:**
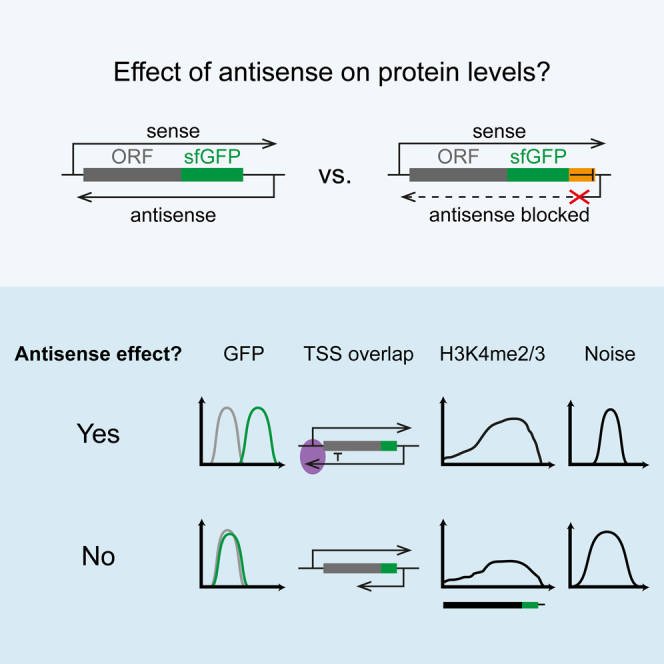

## Teaser

Huber et al. conduct a systematic study of the function of antisense RNAs in yeast by selectively suppressing the antisense transcripts of 188 GFP-tagged genes and quantifying the resulting changes in protein expression levels. The authors use this information to identify features that distinguish functional from non-functional antisense RNAs.

## Introduction

High-throughput technologies such as tiling arrays and deep sequencing enable genome-wide and strand-specific detection of RNAs and have revealed the pervasive nature of transcription in eukaryotic genomes (Bertone et al., 2004, David et al., 2006, Nagalakshmi et al., 2008), resulting in the identification of many classes of non-coding RNAs (ncRNAs). In *Saccharomyces cerevisiae,* ncRNAs typically originate from nucleosome-depleted regions (NDRs), which are frequently associated with bidirectional promoters of protein-coding genes (Neil et al., 2009, Xu et al., 2009). However, such pervasive transcription from NDRs is limited by a combination of transcriptome surveillance mechanisms such as transcription attenuation mediated by the Nrd1-Nab3-Sen1 (NNS) termination complex (Arigo et al., 2006, Schulz et al., 2013), suppression of divergent transcription via histone marks (Churchman and Weissman, 2011, Marquardt et al., 2014, Uprety et al., 2016), or rapid degradation of the resulting transcripts by the exosome (Davis and Ares, 2006, van Dijk et al., 2011, Wyers et al., 2005).

In contrast to exosome-sensitive cryptic unstable transcripts (CUTs), stable unannotated transcripts (SUTs) are readily detectable in wild-type cells, and more than 600 of such transcripts have been annotated in yeast (Xu et al., 2009). When ncRNAs are transcribed in antisense direction to an open reading frame (ORF), they are also referred to as antisense RNAs (asRNAs).

In a number of detailed studies, asRNAs were found to exert important biological functions by regulating the expression of the overlapping gene. For example, the asRNA *RME2* blocks entry into meiosis in haploid yeast cells by repressing transcription elongation of the *IME4* gene (Gelfand et al., 2011, Hongay et al., 2006). Lenstra and colleagues demonstrated that an RNA antisense to *GAL10* prevents transcriptional leakage of both *GAL10* and *GAL1*, thus modulating the responsiveness of the underlying metabolic switch (Lenstra et al., 2015). In addition, strong regulatory functions of asRNAs in yeast have been shown for several genes, including *CDC28* (Nadal-Ribelles et al., 2014), *PHO84* (Camblong et al., 2009, Camblong et al., 2007, Castelnuovo et al., 2013), *PHO5* (Uhler et al., 2007), and *IME1* (van Werven et al., 2012), each with individual mechanistic characteristics different from RNAi, as *S. cerevisiae* lacks a functional RNAi machinery (Drinnenberg et al., 2011, Drinnenberg et al., 2009).

While these cases are well established, our understanding of which asRNAs serve a biological function and whether those share certain characteristics remains incomplete (Pelechano and Steinmetz, 2013). Several high-throughput transcriptome studies generated correlative data on transcript levels of sense-antisense pairs on a genome-wide scale. From these studies, a picture emerges where sense levels are anticorrelated with antisense. However, not all sense transcript levels are affected by changes in antisense transcript levels, and the anticorrelation is often weak (Alcid and Tsukiyama, 2014, Castelnuovo et al., 2014, Schulz et al., 2013, Xu et al., 2011, Xu et al., 2009). Importantly, others have pointed out that there is a lack of anticorrelation at the level of nascent transcription (Murray et al., 2015). In the latter study, Murray and colleagues detected specific chromatin signatures associated with antisense transcription. From this, they hypothesized that the main biological function of asRNAs lies in affecting traits such as expression noise or gene silencing rather than affecting bulk protein abundances. Indeed, studies showing the on/off switching of sense-antisense pairs in different conditions (Lenstra et al., 2015, Nguyen et al., 2014) and suggesting effects of antisense transcription on noise levels (Xu et al., 2011) exist. However, how common such roles are among all antisense transcripts remains to be determined.

Therefore, our understanding of the general principles that govern antisense-dependent gene regulation remains incomplete (Pelechano and Steinmetz, 2013). Some of the main reasons concern methodological difficulties. First, studies that measure protein rather than RNA levels are lacking. Second, datasets typically rely on correlative data and use mutants that interfere with ncRNA stability, since direct ncRNA abrogation is difficult given the overlapping arrangement of sense-antisense pairs. Hence, the causality of sense-antisense regulation is often unclear. In addition, determining the proportion of antisense transcripts that have a biological function is difficult. Finally, it remains an open question which features are predictive of asRNA functionality and whether such features are shared among many asRNAs.

Here, we interfered with antisense transcription of 162 genes and assessed the impact of this disturbance on the level of the expressed protein using single-cell microscopy. The strategy used is based on seamless genomic manipulations (Khmelinskii et al., 2011) and a specific DNA element from the *PHO5* termination region that blocks transcription in a unidirectional manner (Irniger et al., 1991). We applied this strategy to 188 genes in *S. cerevisiae*, 162 of which had been annotated with antisense SUTs. We then assessed the resulting changes of protein levels of GFP-tagged variants of the overlapping genes by high-throughput fluorescence microscopy in four growth conditions and by using flow cytometry. This allowed us to investigate the general impact of asRNAs on protein abundance and gene expression regulation.

## Results

### Antisense Library Construction

**Figure 1:**
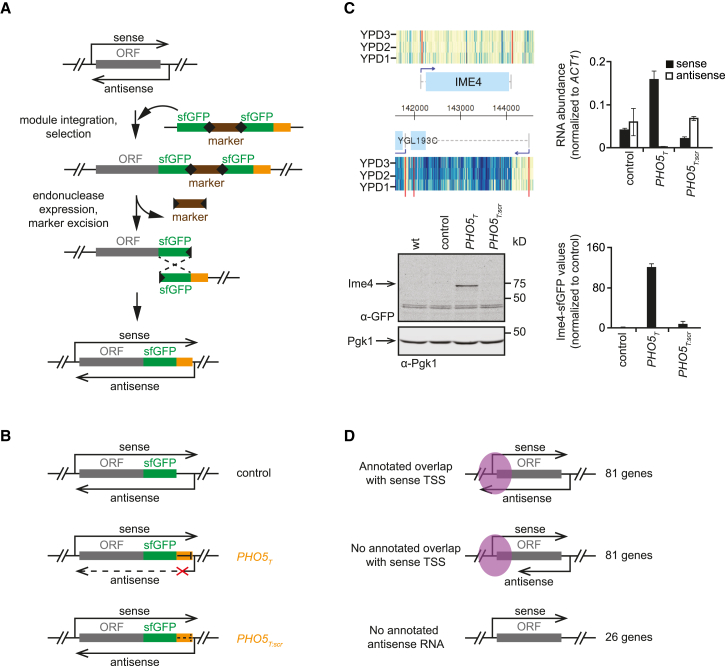
Antisense Deletion Library: Strategy, Library Construction, and Validation (A) Abrogation of gene-specific asRNA transcription using seamless gene tagging. A cassette containing one full sfGFP and a ΔN-sfGFP fragment and two I-SceI sites flanking a counter selectable marker (*URA3*) is inserted using PCR targeting at the 3′ end of the ORF in a strain also containing a Gal1-I-SceI endonuclease cassette. Upon expression of I-SceI on galactose-containing medium, the inserted cassette is cleaved and recombination between the sfGFP repeats occurs with high efficiency. (B) Three different cassettes, containing only sfGFP, or sfGFP and either *PHO5*_*T*_ or *PHO5*_*T:scr*_ were used to generate for each gene three different strains. (C) RNA and protein expression analysis for the *IME4* gene using strains constructed with the method described in (A) and (B). Top left: expression data from tiling arrays of the *IME4* genomic region in haploid cells for the Watson (top) and Crick (bottom) strands are shown. Plots show normalized signal intensities from three hybridizations (YPD1, YPD2, and YPD3) using cells grown in rich medium (YPD). Transcript boundaries are depicted in red, and the darker blue color indicates a higher hybridization signal. Top right: *IME4* RNA and asRNA abundances measured by qRT-PCR using strand specific primers for the sfGFP sequence. The values were normalized to the *ACT1* gene. Bottom: western blotting and quantification of the signal of Ime4-sfGFP (Ime4). Pgk1 was used as a loading control. One representative blot is shown. Quantifications are based on three replicates, normalized using Pgk1 as a reference. Error bars indicate SD. (D) Three categories of genes were chosen for tagging based on the data from Xu et al. (2009), as indicated. The transcript start site (TSS) area of the sense gene is shown in purple.

A previous study on yeast polyadenylation sites reported that a short sequence of ∼100 bp from the 3′ intergenic region of the *PHO5* gene acts as a unidirectional terminator (Irniger et al., 1991). To explore whether such an element could be used for the specific abrogation of antisense transcription, we tailored a strategy based on seamless gene tagging (Khmelinskii et al., 2011). We inserted this fragment, termed *PHO5*_*T*_, and a scrambled control, *PHO5*_*T:scr*_, directly downstream of the stop codon of genes so that no auxiliary sequences such as marker genes were left behind after insertion and seamless marker excision. In order to measure protein levels, we simultaneously fused superfolder GFP (sfGFP; Pédelacq et al., 2006) to the C terminus of the proteins (Figures 1A and 1B). We tested this strategy using *IME4*, a gene that is suppressed by the asRNA *RME2* in haploid yeast (Gelfand et al., 2011, Hongay et al., 2006). We observed Ime4-sfGFP mRNA and protein upregulation and asRNA downregulation upon insertion of the *PHO5*_*T*_ element, but not with the *PHO5*_*T:scr*_ element or when sfGFP was inserted alone (Figure 1C). In diploid yeast, transcription of *RME2* is suppressed (Hongay et al., 2006). Accordingly, using strand-specific qRT-PCR on *IME4* in a diploid context, we could not detect asRNA. Notably, there were no significant differences in sense RNA levels between the three constructs, indicating that *PHO5*_*T*_ and *PHO5*_*T:scr*_ do not, in general, lead to changes in RNA stability or transcription rates when compared to the control (Figure S1A).

We also applied our strategy to *RSC58*, a gene with no reported asRNA. Sense RNA and protein levels did not change (Figures S1B and S1C), indicating that the insertion of *PHO5*_*T*_ and *PHO5*_*T:scr*_ did not affect transcription of this gene. Interestingly, for this gene, qRT-PCR detected low levels of an antisense transcript, which was repressed by the *PHO5*_*T*_ element. This indicates that current SUT/CUT annotations are probably a conservative estimate dictated by the sensitivity of the specific assays used for their detection and that antisense transcription did not influence sense levels of this particular gene.

We conclude that our strategy represents a suitable approach to study the impact of antisense transcription on protein levels by specifically disrupting antisense transcription while leaving sense transcription unaffected.

The above results encouraged us to apply the *PHO5*_*T*_ strategy to study the function of a larger number of asRNAs. To this end, we tagged approximately one-quarter of the 613 yeast ORFs that are annotated with antisense SUTs starting downstream of the STOP codon (Xu et al., 2009), using *sfGFP-PHO5*_*T*_, *sfGFP-PHO5*_*T:scr*_ or *sfGFP* alone. A total of 188 ORFs were chosen randomly from the subset of genes predicted to be not affected by the C-terminal sfGFP tag (based on previous genome-wide datasets; Gavin et al., 2006, Ghaemmaghami et al., 2003, Huh et al., 2003, Khmelinskii et al., 2014). The selected genes differ in the extent to which the asRNA overlaps the sense. For 81 genes, the annotated asRNA overlaps the transcript start site (TSS). For another 81 genes, the asRNA terminates earlier. In addition, we selected 26 genes that contain no annotated asRNA (Figure 1D; Table S1). To control for PCR errors associated with PCR targeting during library construction, we used two biological replicates of each strain for subsequent experiments (Experimental Procedures).

### Analysis of the Antisense Library Using Quantitative Microscopy

**Figure 2:**
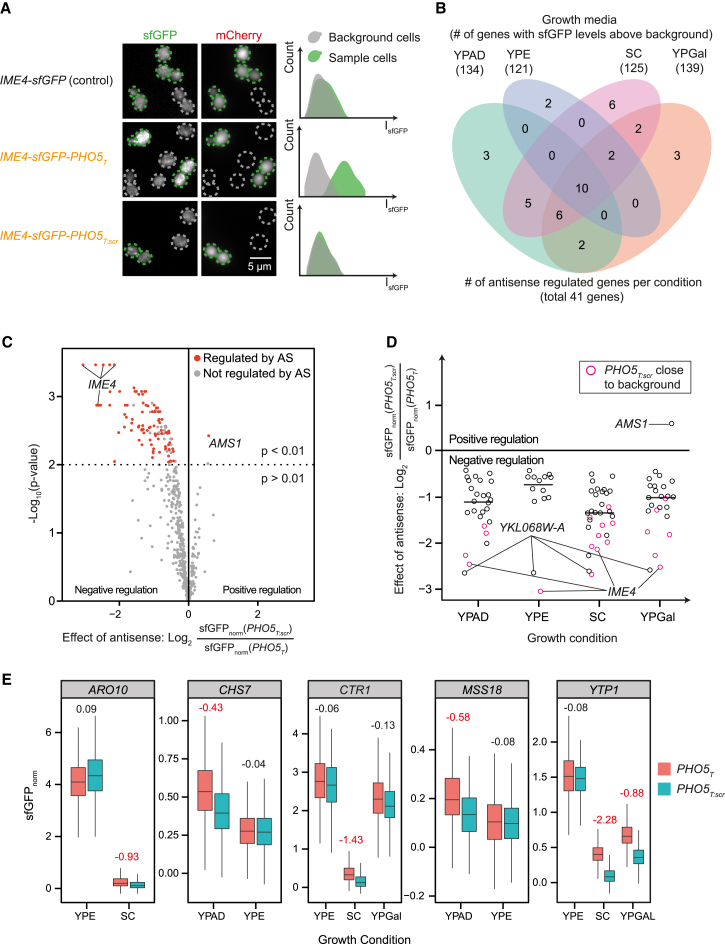
High-Throughput Quantitative Microscopy Identifies Antisense-Regulated Genes (A) Cells were imaged, and whole-cell fluorescence intensities were measured using co-cultured non-fluorescent cells (identified by differential labeling with an mCherry reporter) for background subtraction and fluorescence normalization on a per-well basis (Experimental Procedures). Example cells for *IME4* in YPAD are shown. Gray, non-fluorescent cells; green, sfGFP-expressing sample cells. (B) Venn diagram showing the number of antisense-regulated genes under the different growth conditions. Numbers in parentheses indicate the total number of genes with sfGFP levels above background (for *PHO5*_*T*_ and/or *PHO5*_*T:scr*_). In total, 41 genes were found to be antisense regulated under at least one condition. (C) A volcano plot of p values versus log2-fold changes: ratio of sfGFP levels between *PHO5*_*T:scr*_ (with antisense) and *PHO5*_*T*_ (no antisense) strains. Each dot represents one gene in one growth condition. Red indicates genes that met our criteria for being regulated by antisense (see text). (D) Log2-fold changes between *PHO5*_*T:scr*_ and *PHO5*_*T*_ for each growth condition. Selected examples are labeled. Short black bars indicate the median. Colored circles indicate genes whose sfGFP intensities could not be reliably distinguished from background for the *PHO5*_*T:scr*_ construct. To be able to visualize these genes in the diagram, a small offset for the *PHO5*_*T:scr*_ expression value was introduced if necessary (see Experimental Procedures for details). (E) Box plots of sfGFP intensities (sfGFP_norm_, normalized to the fluorescence of the control cells without sfGFP) are shown for genes found to exhibit condition-specific regulation by antisense. Only the conditions differing in their regulatory strength are shown. Numbers indicate the log2-fold change of *PHO5*_*T:scr*_ over *PHO5*_*T*_. Red numbers indicate that the gene was found to be significantly regulated in that condition and fill colors represent the construct (*PHO5*_*T*_ or *PHO5*_*T:scr*_). Populations of both biological replicates were pooled in the boxplots for simplicity.

To determine the effect of antisense transcription on protein abundance, we compared sfGFP intensities of the *PHO5*_*T*_, *PHO5*_*T:scr*_ and control strains in our library. For this purpose, we established and validated a high-throughput quantitative fluorescence microscopy pipeline and quantified total cellular sfGFP fluorescence in >100 cells for each of the strains using automated image acquisition and analysis (Figure 2A; Experimental Procedures). We used mid-log phase cells grown at 30°C using the four growth conditions in which the SUTs had been annotated (Xu et al., 2009): rich medium with either glucose, galactose, or ethanol (YPAD, YPGal, and YPE) and synthetic complete medium (SC). Approximately 5% of the data points were removed based on different quality control criteria (Experimental Procedures; Figures S2A–S2C; Tables S2 and S3). The measured sfGFP intensities are given in Table S2. Because the original RNA expression dataset used to annotate the SUTs was obtained from a diploid strain (Xu et al., 2009), we repeated the strand-specific tiling array assay using the haploid library background strain and using the same four growth conditions. Microscopy-based protein measurements and RNA levels measured by tiling arrays exhibited the correlation expected from the literature (R^2^ between 0.49 and 0.62; Figure S2D; Csárdi et al., 2015). Depending on the growth condition, between 121 and 139 genes showed sfGFP fluorescence above background (Figure 2B), even though our library was enriched for low-expressed genes (Figure S2E). This demonstrates the validity and sensitivity of our approach.

Next, we identified genes where the suppression of antisense transcription led to significant changes in protein abundance (Figures 2B and 2C; the genes are listed in Table S4). We calculated the average fold change of the sfGFP levels between the *PHO5*_*T:scr*_ and the *PHO5*_*T*_ constructs. These values were well reproducible, as judged by a repetition of the experiment using a subset of the strains (R^2^ = 0.81; Figure S2F). p values were obtained using a linear modeling approach (Experimental Procedures), and the significance threshold was set to p < 0.01. In addition, we stipulated that protein expression values of *PHO5*_*T:scr*_ and sfGFP (*wt*) constructs be within a 50% expression range of each other, which was true for all but six cases, and that protein abundances between the *PHO5*_*T*_ and *PHO5*_*T*:scr_ constructs differed more than biological replicates (Experimental Procedures). Depending on the growth condition, we found between 14 out of 121 (12%) and 31 out of 125 (25%) genes with significant differences between the *PHO5*_*T*_ and *PHO5*_*T*:scr_ constructs. This corresponds to 41 genes where significant regulation by antisense was observed under at least one condition (Figures 2B and 2C).

On average, the presence of antisense led to an ∼2-fold reduction in protein abundance, with effect sizes ranging from 1.35-fold (*CHS7*) to 6.3-fold (*YKL068W-A*; Figure 2D). Only one gene, *AMS1*, was positively regulated by antisense (Figures 2C and 2D). Depending on the growth condition, between 1 and 9 of the antisense-regulated genes had in the *PHO5*_*T:scr*_ background sfGFP levels that were below the threshold we determined for calling a gene “expressed,” suggesting a potential antisense-dependent on/off switch for these genes (Figure 2D; Table S4).

Next, we looked for genes that were regulated by antisense under only a subset of the conditions where expression was observed (Experimental Procedures). We found five such genes (Figure 2E). Interestingly, in all of those cases regulation was absent in YPE. Mostly, however, regulation between conditions did not change significantly.

To validate our findings, we used northern blotting to detect the mRNAs of a selected number of antisense-regulated genes (Figure S3A). In agreement with microscopy results, the mRNA levels were higher in the strains with the *PHO5*_*T*_ than in the strains with the control or *PHO5*_*T:scr*_ constructs. In some instances, we noted that the sense transcripts were slightly shorter for *PHO5*_*T*_ than for *PHO5*_*T:scr*_. It is currently unclear whether this is due to an antisense-specific regulation of the sense polyadenylation site (see Discussion).

These data shed light on which antisense transcripts influence sense protein levels in a native context and provide evidence that SUTs can change the protein abundance of their parent genes in at least 12%–25% of the cases. The majority of the genes was found to be negatively regulated by antisense.

### The Regulatory Effect of Antisense Correlates with Antisense Levels and Anticorrelates with Sense Levels

**Figure 3:**
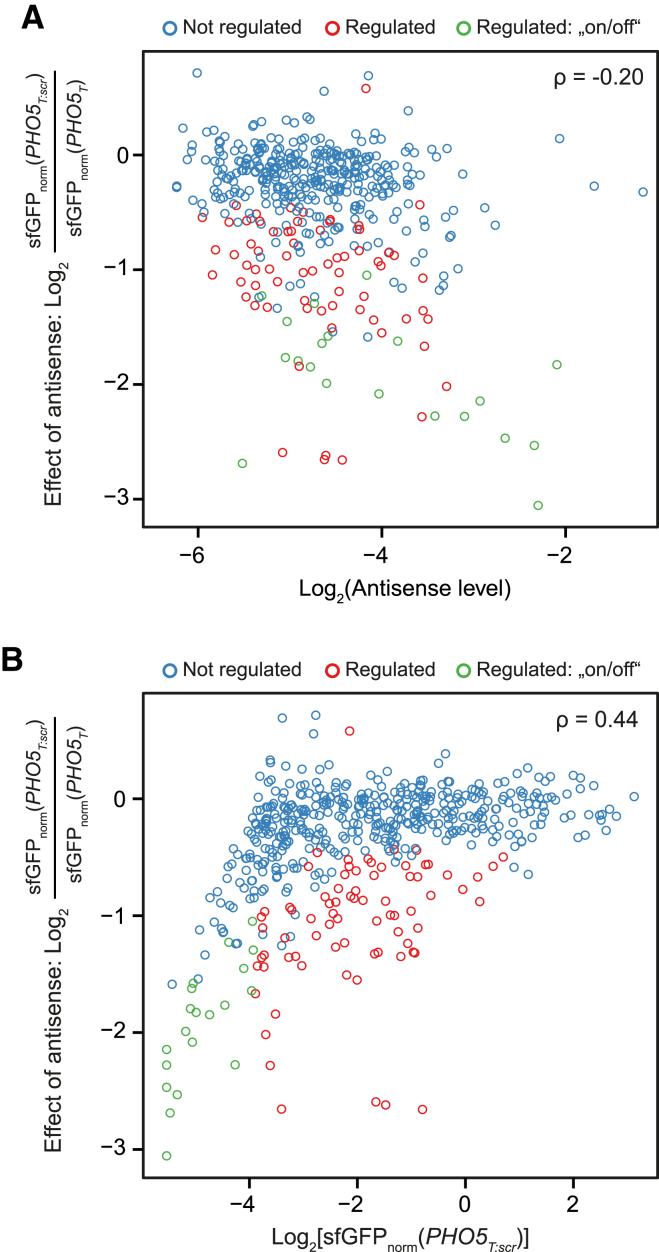
Correlation of Regulation by Antisense with Antisense and Protein Levels (A) The effect of antisense on protein levels (log2-fold change of sfGFP levels between *PHO5*_*T:scr*_ and *PHO5*_*T*_) versus antisense transcript levels. Data obtained for all conditions from fluorescence microscopy and from tiling arrays are shown. These were obtained using the same strain background and the same growth conditions. Blue circles represent genes not found to be regulated by antisense. Red and green circles indicate genes with regulation by antisense with the latter being subject to an antisense-dependent on/off switch (see Figure 2 and main text). Spearman’s correlation coefficient ρ is indicated in the plot. (B) As in (A), but with sfGFP intensities of the *PHO5*_*T:scr*_ construct plotted on the x axis.

In order to better understand the relationship of antisense-dependent effects and gene expression, we first explored whether there is a correlation between the strength of the effect of antisense regulation (microscopy data) and sense/antisense RNA levels (tiling array data). We found that the repressive effect of antisense is stronger with increasing antisense levels (Spearman’s ρ = −0.20; Figures 3A and S4A). However, the antisense to sense ratio for a particular gene was a stronger predictor for the repressive effect (ρ = −0.43; Figures S4B and S4C). Finally, we found that the repressive effect of antisense declines with increasing protein expression levels (ρ = 0.44; Figures 3B and S4D) and that highly expressed genes (sfGFP_norm_ of *PHO5*_*T:scr*_ > 1.63) did not exhibit regulation by antisense. These trends suggest that sense and antisense inhibit one another. However, there is also a considerable variability between genes in their susceptibility for antisense dependent regulation.

### Regulation by Antisense Correlates with Transcript Start Site Overlap

**Figure 4:**
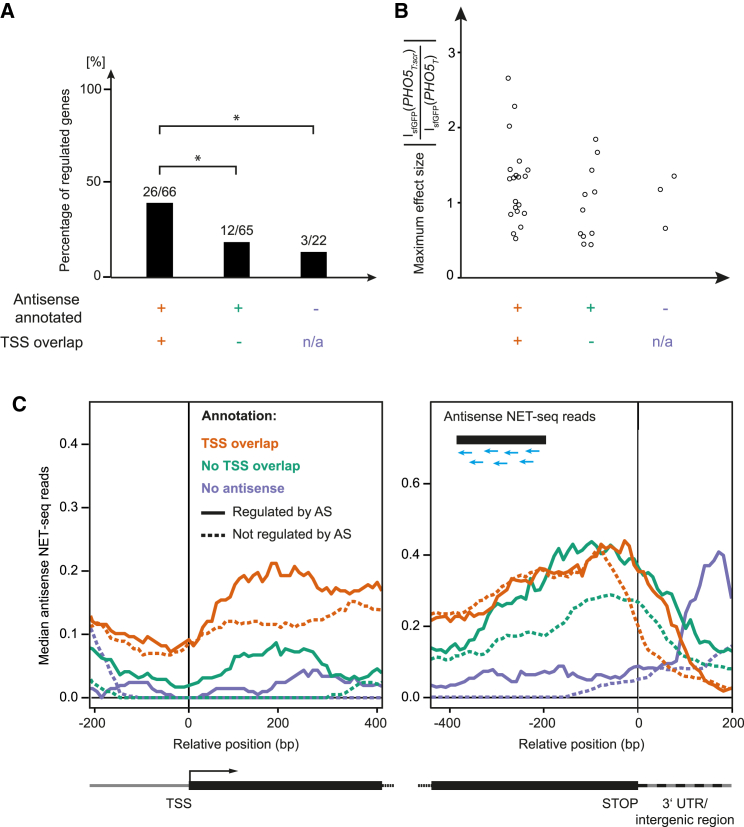
TSS Overlap Correlates with Regulation by Antisense Transcription (A) Genes that were tested for antisense regulation in at least one condition were divided into three groups that differ in the degree of antisense overlap with the sense (see main text). Next, the number of genes that were regulated by antisense in at least one condition was determined. The frequency of genes regulated in at least one condition is significantly higher when the antisense is annotated to overlap the TSS than in the group without annotated TSS overlap or the group where no antisense has been annotated (Fisher’s exact test, p < 0.05). (B) For every gene found to be regulated in at least one condition the maximum significant log2-fold-change between PHO5_T:scr_ and PHO5_T_ was determined across growth conditions. This does not include genes with an “all or nothing” behavior (see main text). (C) Genes were aligned at their TSS (left) or stop codons (right). The aligned NET-seq traces are shown using group-wise smoothed medians (Experimental Procedures). Colors indicate the extent of SUT overlap with the sense as annotated in Xu et al. (2009). Solid lines indicate antisense-regulated genes, whereas dashed lines indicate non-regulated genes.

Previous studies showed that antisense transcription across the TSS of the overlapping gene may cause repression of the latter mediated by a variety of chromatin-dependent mechanisms (Castelnuovo et al., 2014, Castelnuovo et al., 2013, van Werven et al., 2012). Consistent with this, we found that genes whose antisense transcript overlaps the TSS had a higher chance of being antisense regulated than genes whose antisense transcript terminated earlier (Figure 4A). While the size of the antisense effect was not higher in the case of TSS overlap (Figure 4B), more genes showed an “all or nothing” behavior (11/26 in set 1 versus 2/12 in set 2; Table S4).

TSS overlaps were classified based on tiling array data. Native elongating transcript sequencing (NET-seq; Churchman and Weissman, 2011) is a complementary method that reports on the positions of actively transcribing RNA polymerase complexes. It is thus capable of detecting transcriptional events not visible by bulk analysis methods such as tiling arrays. We made use of available NET-seq data (Churchman and Weissman, 2011) and plotted their read numbers in antisense direction relative to the position of the TSS or the stop codon of the genes in our dataset (Figure 4C). This revealed for antisense-regulated genes the presence of active antisense transcription at the sense TSS even if the respective SUTs were annotated to not overlap the TSS. Similarly, regulated genes without annotated SUTs also showed reads near the TSS. In contrast, non-regulated genes in those two groups did not show any reads near the TSS. For those genes where TSS overlap was annotated, both regulated and non-regulated genes showed reads near the TSS. This suggests that TSS overlap is not a sufficient determinant of regulation. As an alternative explanation, we noted that the peak of antisense transcription for the non-regulated genes was clearly shifted into the coding sequence at the stop codon. This could mean that some of the antisense transcripts in this group initiated in the coding region, which would result in a failure of termination by the *PHO5*_*T*_ construct.

In summary, the NET-seq data analysis further strengthens the notion of a functional connection between antisense regulation and antisense transcription across the sense TSS.

### Antisense-Regulated Genes Show Increased H3K4 Di- and Trimethylation

**Figure 5:**
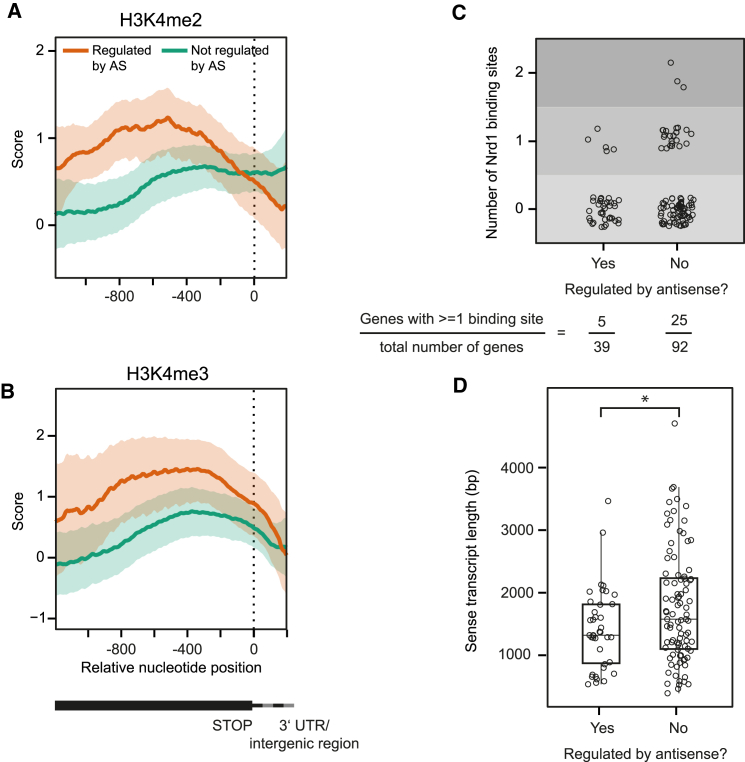
Features Correlating with Regulation by Antisense (A and B) H3K4me2 and H3K4me3 densities in antisense-regulated (red) versus non-regulated (green) genes. All genes were aligned at their stop codons. Lines and ribbons indicate bootstrapping-based mean and 95% confidence interval estimates, respectively. (C) The number of Nrd1 binding sites as determined previously (Hogan et al., 2008) was determined for all transcripts in our library and the number of binding sites per gene is shown, grouped by whether the gene was found to be regulated by antisense in at least one condition or not. (D) Transcript lengths of genes grouped by whether they were regulated by antisense in at least one condition or not. The groups are significantly different (Wilcoxon’s rank sum test, p < 0.05).

We proceeded to identify more features correlating with regulation by antisense. To ensure that potential differences are not due to differences in transcript levels, we did not consider genes without annotated antisense for this and subsequent comparisons. Of the remaining genes, antisense regulated and non-regulated genes showed similar sense and antisense levels, as measured by tiling arrays (Figures S5A and S5B). Since histone modifications have been implicated in antisense-dependent gene regulation (Camblong et al., 2007, Castelnuovo et al., 2014, Uhler et al., 2007, van Werven et al., 2012), we first tested whether specific chromatin modifications are associated with functional antisense transcripts. We made use of available datasets (Experimental Procedures) and compared histone traces of antisense-regulated and non-regulated genes relative to their TSSs and their stop codons. This revealed increased H3K4 di- and trimethylation (H3K4me2/3) at the 3′ end of the regulated genes (Figures 5A and 5B). H3R2me2 is known to counteract H3K4me3 (Kirmizis et al., 2007). Consistent with this, H3R2me2 was decreased in this region (Figure S5C). We could not detect significant changes for other histone modifications (Table S5). This implies a functional relevance of H3K4 methylation in gene regulation by antisense transcripts.

### Lack of Obvious Sequence Features Associated with Regulation by Antisense

Next, we searched for sequence motifs associated with genes in either the antisense regulated or the non-regulated group. Extensive searches using databases of annotated DNA-protein binding sites, a catalog of RNA-protein interaction sites, and de novo motif identification tools did not reveal any significant results (Bailey et al., 2009, Hogan et al., 2008, Shannon, 2015, Venters et al., 2011; see Experimental Procedures for further details). Of special interest in this respect are motifs specific for transcription attenuation by the NNS complex known to limit non-coding transcription of CUTs in yeast (Arigo et al., 2006, Schulz et al., 2013). The involvement of the NNS complex in early termination and the correlation of regulation by antisense and TSS overlap suggested that non-regulated genes might be enriched in Nrd1-Nab3 binding sites. To test this, we used previously published data on Nrd1 binding sites (Hogan et al., 2008). The fraction of genes that displayed at least one Nrd1 binding site in antisense direction was increased in non-regulated genes versus regulated ones (25 out of 92 versus 5 out of 39), but this increase was not significant (Figure 5C) and may in part be due to the increased length of non-regulated genes versus regulated ones (Figure 5D). A similar comparison using strand-specific photoactivatable ribonucleoside-enhanced crosslinking and immunoprecipitation (PAR-CLIP) data (Creamer et al., 2011) did not reveal any significant differences either (data not shown).

Together, this suggests that antisense transcripts do not contain sequence information sufficient to predict their regulatory potential.

### Antisense-Regulated Genes Show Reduced Protein Expression Noise

**Figure 6:**
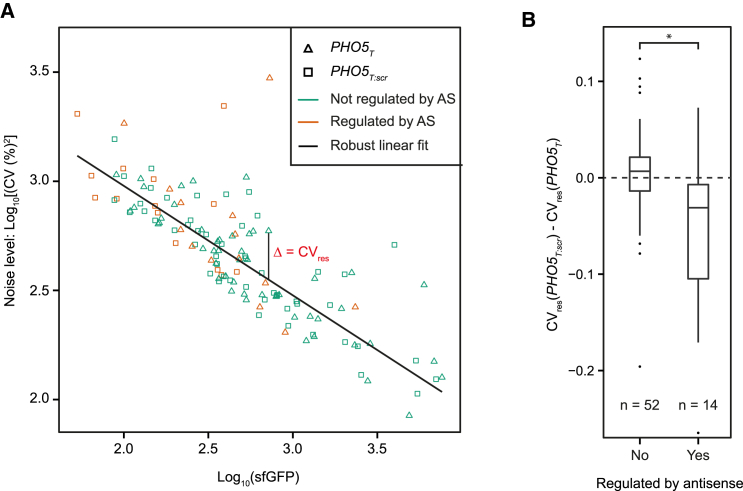
Impact of Antisense Suppression on Gene Expression Noise (A) Log_10_[CV (%)^2^] versus log_10_(sfGFP) of 66 genes permit robust linear fitting (solid black line) and the calculation of noise levels corrected for differences in abundance (CV_res_; one example is indicated). Different fits are possible, but the result (shown in B) is the same in all cases (see also Figure S6D and Experimental Procedures. *PHO5*_*T*_ and *PHO5*_*T:scr*_ strains are indicated by triangles and squares, respectively. Genes regulated by antisense are shown in orange, whereas non-regulated genes are shown in green. (B) Distribution of the gene-wise differences of CV_res_ values of *PHO5*_*T:scr*_ minus *PHO5*_*T*_ constructs from (A), grouped depending on whether the genes were found to be antisense regulated under the growth condition used for FACS (SC) or not. The difference between the groups is significant (Wilcoxon’s rank sum test, p < 0.05).

Previous studies discussed a correlation between the presence of an overlapping antisense transcript and the degree of cell-to-cell variability (“noise”) in protein abundance (Pelechano and Steinmetz, 2013, Xu et al., 2011). We followed the flow cytometry based noise measurement strategy of Newman et al. (Newman et al., 2006; see Experimental Procedures for details) to investigate differences in noise levels between *PHO5*_*T*_ and *PHO5*_*T:scr*_ strains. Briefly, a special gating procedure is applied to select for a homogeneous population of unbudded G1 cells (Figure S6A). Consequently, sources of extrinsic noise are minimized and the resulting coefficient of variation (CV % = (SD/mean) × 100) values are composed of intrinsic noise and extrinsic noise specific to the pathway that regulates that gene’s expression (Newman et al., 2006, Raser and O’Shea, 2005). The limited sensitivity of flow cytometry meant that we could only measure noise levels for 66 genes, including 14 antisense-regulated ones (see Figure S6B for a comparison with microscopy). As previously reported, we observed an inverse proportional relationship between CV^2^ and protein abundance (Figure 6A; Newman et al., 2006, Paulsson, 2004). For the antisense-regulated genes, protein abundances of the *PHO5*_*T:scr*_ strains are on average smaller than for the *PHO5*_*T*_ strains (Figure S6B). This also applies for the genes considered to be not regulated by antisense, albeit in a non-significant manner. Because of this slight deviation from zero, a direct comparison of noise between the two categories will be strongly affected by changes in protein abundances (Figure S6C). To obtain noise levels independent of this confounding influence, we adapted the analysis procedure of Newman et al., 2006 (Newman et al., 2006). We calculated the residuals of each CV value (CV_res_) to a robust regression model of the CV values (Figure 6A). By calculating the difference in the CV_res_ values of *PHO5*_*T:scr*_ − *PHO5*_*T*_, one can compare the noise levels of the two constructs for a given gene and for different categories in general. Interestingly, the distributions of the gene-wise differences in CV_res_ values were significantly smaller for antisense regulated genes than for non-regulated ones (Wilcoxon’s rank sum test, p < 0.05; Figure 6B) and were mainly negative. We conclude that, provided that a gene is antisense regulated, noise levels are reduced in the presence of antisense transcription.

## Discussion

Reports that provide experimental evidence for the implications of antisense regulation on the expressed protein amounts of the overlapping sense gene are scarce, with most studies focusing on RNA levels or on individual genes. Here, we investigated the impact of SUT antisense transcripts on expressed protein amounts for a larger group of genes. We employed yeast high-throughput strain construction and a strategy to specifically terminate antisense transcription and used this to investigate the function of >150 SUTs in regulating protein levels under four growth conditions. We found that SUTs led to a significant reduction of protein abundance for approximately one quarter of the genes. Therefore, the mere presence of an antisense SUT for a given ORF is no indication for a functional role of this ncRNA. Our results provide experimental confirmation for the previous observations from transcriptomics studies that antisense transcription seems to generally repress sense expression. Our finding that 41 out of 152 genes with detectable GFP signals exhibit antisense-dependent gene regulation under at least one condition is consistent with the idea that only a fraction of genes are sensitive to antisense transcription (Alcid and Tsukiyama, 2014, Castelnuovo et al., 2014, Schulz et al., 2013, Xu et al., 2009). We observed that the regulatory effect of antisense SUTs tended to be weak, leading on average to a 50% reduction in protein levels. Our data show that both antisense levels and antisense/sense ratios correlated with the repressive effect of antisense. At high sense levels, repressive effects were markedly reduced. We also found that the regulatory potential of antisense transcripts was increased in the case of TSS overlap and was paralleled by a reduction in protein expression noise.

The strategy to employ a DNA element (*PHO5*_*T*_) to abrogate SUTs in a strand-specific manner bears the risk that the introduced element introduces additional disturbances, as is the case for any gene- and protein-tagging strategy. For example, a certain number of antisense transcripts may be initiated within the ORF of the gene, in which case the *PHO5*_*T*_ strategy does not work. This might be the case for a number of non-regulated genes with TSS-overlapping SUTs as indicated by a pronounced shift of the NET-seq read maximum toward the inside of the coding region in this group (Figure 4C). Inhibition of an antisense transcript could also lead to the derepression of a cryptic downstream antisense transcript, thus reconstituting antisense repression. Moreover, the approach we used may occasionally introduce artifacts. For example, the actual strength and unidirectionality of our terminator may depend on the genomic context (Guo and Sherman, 1996). Northern blots showed that in some cases, the sense transcripts with the *PHO5*_*T*_ were shorter than expected (Figure S3A). This could be due to premature termination or selection of an alternative poly(A) site as a function of the *PHO5*_*T*_ element. Equally well, however, this could be caused as a function of the abrogated antisense RNA. Nevertheless, we are confident that the *PHO5*_*T*_ element functions as expected in a majority of the cases. First, there was a neutral effect of both the *PHO5*_*T*_ and the *PHO5*_*T:scr*_ constructs on *IME4-sfGFP* RNA levels in a diploid background, where the *RME2* antisense transcript is efficiently repressed. Second, we would expect that general premature termination of transcripts results in decreased stability. The results for *IME4*, the absence of mRNA degradation products in our northern blots and the fact that protein levels for the *PHO5*_*T*_ strains were usually increased indicate that this is not a general concern.

Alternative approaches to study antisense transcription employ mutants in genes that globally affect the stability and transcription of non-coding RNAs. However, such mutations are likely to influence a cell’s RNA homeostasis globally, and hence the predictive value for the assignment of regulatory functions of antisense transcripts is limited. Interestingly, the clustered regularly interspaced short palindromic repeats (CRISPR)/Cas9 system has been recently used in yeast to suppress a ncRNA in a strand-specific and position-dependent manner (Lenstra et al., 2015). This offers the advantage of not manipulating the endogenous locus. However, it requires gene-specific optimization in order to identify guide RNAs that are effective in quenching the antisense transcript (Lenstra et al., 2015), which makes it difficult to use this strategy in systemic studies.

Antisense regulation might affect gene noise with or without affecting the average expression level of a gene. Interestingly, we found that regulation of protein amounts by antisense results in a concomitant reduction in protein expression noise. Our fluorescence-activated cell sorting (FACS) measurement could only assess genes that are well expressed. For this subset of antisense-regulated genes, our result is in contrast to the view that antisense transcription increases the noise of genes in the “on” state (Pelechano and Steinmetz, 2013, Xu et al., 2011). It will be interesting to study whether the effect we observed results from antisense-regulated genes being intrinsically less noisy or because the pathways involved in antisense-dependent repression act as extrinsic low-level noise sources.

The fact that 13 of 41 genes showed full repression of protein expression under at least one growth condition (within the limits of sfGFP detection; Figure 2D; Table S4) supports the idea that antisense is frequently involved in the switching between “on” and “off” states and serves to suppress the leakiness of gene expression (Castelnuovo et al., 2013, Hongay et al., 2006, Lenstra et al., 2015, Xu et al., 2011). However, whether these potential on/off switches are associated with a physiological function remains to be determined for each individual gene.

What distinguishes antisense-regulated genes from non-regulated ones? First, our data indicate that increasing antisense levels led to a stronger regulation by antisense while higher sense levels reduced such an effect. This favors the idea that sense and antisense competitively inhibit each other as studied in detail in a previous report using an artificial gene construct in yeast (Buetti-Dinh et al., 2009). However, in contrast to that study, we could not observe a similar decrease in the repressive effect for weakly expressed genes.

Next, we found that antisense transcription overlapping the TSS is a strong predictor for the functionality of antisense transcription. NET-seq data revealed reads around the TSS even for those genes where tiling array experiments did not detect transcripts in that region. This suggests that the act of antisense transcription rather than the amount of the antisense transcript is of functional importance. This would also imply that the distinction between different types of ncRNAs such as CUTs/XUTs/NUTs/SUTs, while interesting in terms of RNA metabolism, may be less important from a functional point of view. For example, Castelnuovo and colleagues presented evidence that led them to hypothesize that the frequency of CUTs escaping early termination and extending into the sense TSS is inversely correlated with sense levels (Castelnuovo et al., 2014, Castelnuovo et al., 2013). We propose that similar mechanisms are at work for SUTs. In this respect, it is unclear which role the NNS complex plays in regulating protein abundance by antisense SUTs. While we found a slight increase in the number of Nrd1 binding sites in the non-regulated gene set, this increase was not significant. At the same time, early termination events are a mechanism that could help to explain how antisense SUTs are prevented from reaching the sense TSS. The fact that non-regulated genes are longer than antisense regulated ones could further decrease the chance of TSS overlap. We speculate that the impact of antisense may be modulated by a combination of strand-specific termination signals and a relative shift of TSS positions, for example by changing the lengths or initiation sites of transcription units. We also did not find any other motifs or protein binding sites associated with antisense-dependent gene regulation. Considering the importance of where in the gene antisense transcription takes place, it is possible that narrowing motif searches down to certain positions in the gene will reveal sequence motifs missed in our analysis.

When comparing histone modification profiles across the genes of interest we found a significant increase in H3K4me2/3 distribution at the end of antisense-regulated genes. H3K4me3 is a modification associated with actively transcribed genes (Pokholok et al., 2005). Thus, one explanation might be that transcription of the antisense RNA in the regulated genes is more active in certain regions and thus redistributes this mark into the gene body, as was proposed in a recent study (Murray et al., 2015). H3K4me3 has also been linked to *SET1*-dependent antisense transcription (Castelnuovo et al., 2014, Margaritis et al., 2012). Our data provide evidence for a widespread functional role of this modification in gene regulation by SUTs. No other histone marks were found to be associated with antisense dependent gene regulation, suggesting that they are not involved in the regulation of protein amounts.

Even genes whose antisense SUTs overlapped the TSS were not always regulated by antisense transcription. This indicates that TSS overlap is either not sufficient to impose antisense-dependent regulation or that certain factors blocked such a repression under the conditions tested. Other possibilities include that the regulated genes were more responsive to certain chromatin modifications under the conditions tested, as exemplified by the genes subject to condition-specific regulation.

In summary, we conclude that the majority of antisense transcripts are unlikely to be effector molecules whose synthesis and presence is involved in regulating the abundance of the sense genes. A smaller fraction of antisense transcripts exhibit weak suppressive and denoising functions that may in some cases lead to a complete shutdown of the sense gene. Given that cases of strong and functional antisense-dependent gene regulation have been observed in yeast, this argues that single antisense transcripts may acquire new roles to regulate the sense gene by making use of a variety of different mechanisms that differ from gene to gene. This leaves room for the evolution of gene-specific mechanisms by which antisense transcription may acquire new physiologically relevant regulatory functions.

## Experimental Procedures

### Yeast Strains and Culturing Conditions

Yeast cells were grown according to standard methods (Sherman, 2002). Cultures were grown to logarithmic phase (optical density 600 [OD_600_] between 0.5 and 1.0) unless otherwise stated. See Supplemental Experimental Procedures for a list of strains and growth media.

### Antisense Library Construction

Details about plasmids and strains are listed in Supplemental Experimental Procedures. Briefly, gene-specific oligos containing S2/S3 annealing sites (Knop et al., 1999) for the template cassettes (pMaM201 with *sfGFP-PHO5*_*T*_, pMaM203 with *sfGFP-PHO5*_*T:scr*_, and pMaM175 with *sfGFP*) were used to generate PCR products using a high-fidelity polymerase. Strain YMaM330 was used for transformation, and for each transformation, six clones were picked, singled out, and validated using colony PCR.

### RNA Extractions and Northern Blots

For all methods, total RNA was extracted using a hot phenol protocol (Collart and Oliviero, 2001). Remaining DNA was removed using the TURBO DNA-free Kit (Life Technologies). Northern blotting is explained in Supplemental Experimental Procedures.

### Tiling Arrays

The background strain of the antisense library (YMaM330) was grown to mid-log phase in one of the four growth media (YPAD, YPGal, YPE, or SC), and total RNA was extracted and hybridized to tiling arrays as described previously (Xu et al., 2009). The dataset for the whole genome is available in a searchable web database (http://steinmetzlab.embl.de//cgi-bin/viewKnopLabArray.pl?showSamples=KnopHaploidTest2&type=heatmap).

### Fluorescence Microscopy

Details about imaging and image processing can be found in Supplemental Experimental Procedures. Briefly, sample cells were mixed 1:1 with sfGFP-negative control cells for background subtraction and normalization on a well-by-well basis. Exponentially growing cells were fixed and seeded on 384-well microscopy plates. Imaging was performed on a Nikon Ti-E screening wide-field epifluorescence microscope using different exposure times for sfGFP and including controls to correct for shading artifacts. Image post-processing, quantification, and quality control were performed using custom scripts in ImageJ, MATLAB (MathWorks), and R (R Core Team, 2015). The whole single-cell dataset can be downloaded from the University of Heidelberg heiDATA Dataverse Network (http://dx.doi.org/10.11588/data/10073). Scripts and raw imaging data are available upon request.

### Bioinformatic and Statistical Analyses

Data analyses were mainly performed using the open source software *R* (R Core Team, 2015), making extensive use of the Bioconductor framework (Huber et al., 2015) and numerous publicly available datasets. Please see Supplemental Experimental Procedures for further details. Scripts are available upon request.

### Flow Cytometry

Cells were grown to mid-log phase in SC medium in 96-well plates and analyzed using a flow cytometer equipped with a high throughput stage (BD FACSCanto RUO HTS). 100,000 events were recorded per well. Gating and calculations to obtain noise estimates were done in R with the help of the flowCore package (Ellis et al., 2015), essentially using the method of Newman et al. (2006). See Supplemental Experimental Procedures for further details. Scripts and data files can be downloaded from the University of Heidelberg heiDATA Dataverse Network (http://dx.doi.org/10.11588/data/10073).

### Western Blots

Proteins were extracted from mid log phase cell cultures grown in the respective medium using the trichloroacetic acid (TCA) method (Knop et al., 1999). Proteins were then separated by SDS-PAGE as described (Laemmli, 1970) and transferred to nitrocellulose membranes using a semi-wet blotter (XCell II Blot Module; Invitrogen). Membranes were incubated overnight with primary anti-GFP antibodies (Abcam) or anti-PGK1 antibodies (Molecular Probes). Secondary antibodies were labeled with Alexa_680_ (Invitrogen) or IRDye_800_ (Rockland Immunochemicals). Detection and quantification was performed with an Odyssey Infrared Imaging System (Li-Cor Biosciences).

### qRT-PCRs

DNase-treated RNA from exponentially growing yeast cells was used as an input for reverse transcription using 2 pmol gene-specific primer and 1 μg RNA using SuperScript III reverse transcriptase (Invitrogen) according to the manufacturer’s instructions but supplemented with 20 μg/ml actinomycin D to ensure strand specificity of the reverse transcription (Perocchi et al., 2007). For qPCR, cDNA samples and -RT controls were diluted to 1 ml, and 2.5 μl were amplified using the LightCycler 480 SYBR Green I Master Mix (Roche). Actin mRNA was used as the reference gene.

## Author Contributions

M.K. conceived the project with input from A.K. and L.M.S.; A.K., M.M., and D.B. constructed the library; F.H. and D.B. designed and analyzed the experiments with input from I.G.; D.B. and F.H. performed the microscopy with technical assistance from P.T.; I.G. performed and analyzed the tiling array experiments; F.H. performed bioinformatics and statistical analyses with input from I.G.; all authors commented on the manuscript; and F.H., D.B., and M.K. wrote the manuscript.

## Accession Numbers

The accession number for the tiling array data reported in this paper is ArrayExpress: E-MTAB-4731.

## Supplemental Information

Document S1. Supplemental Experimental Procedures, Figures S1–S6, and Tables S3–S5

Table S1. Oligos Used for Antisense Library Construction, Related to Figure 1

Table S2. Microscopy Measurement Values and Quality Control Results of Microscopy, Related to Figure 2

Document S2. Article plus Supplemental Information
