# Supplementary material for: Protein Abundance Control by Non-coding Antisense Transcription
Source: Cell Rep. 2016 Jun 9;15(12):2625–36. doi: 10.1016/j.celrep.2016.05.043 (PMC4920891; doi:10.1016/j.celrep.2016.05.043)
Supplement: Document S1. Supplemental Experimental Procedures, Figures S1–S6, and Tables S3–S5 [file mmc1.pdf]

Cell Reports, Volume *15*

Supplemental Information

Protein Abundance Control

by Non-coding Antisense Transcription

Florian Huber, Daria Bunina, Ishaan Gupta, Anton Khmelinskii, Matthias Meurer, Patrick Theer, Lars M. Steinmetz, and Michael Knop

Figure S1

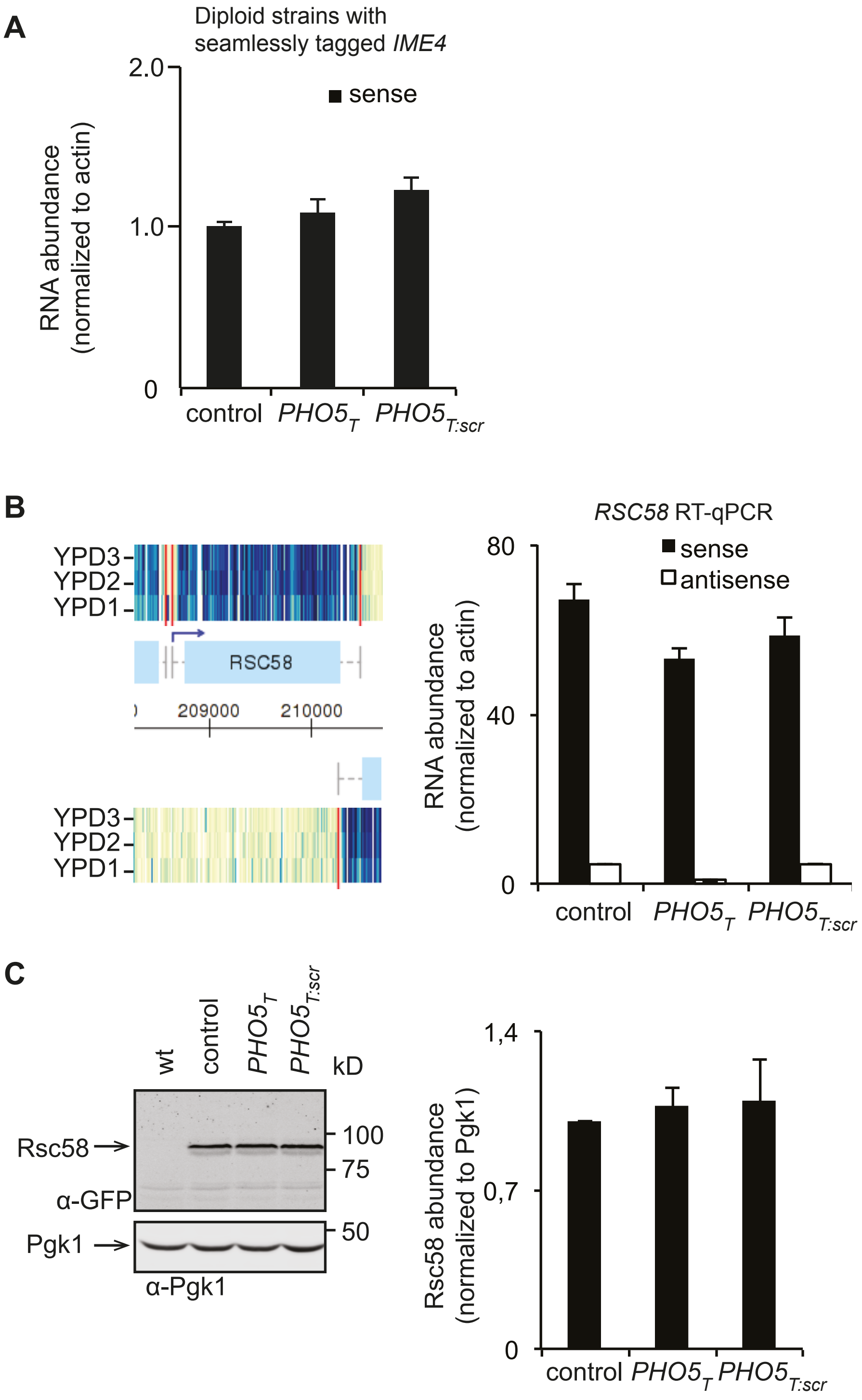
A
Diploid strains with seamlessly tagged IME4
sense
RNA abundance (normalized to actin)
2.0
1.0
0
control
PHO5T
PHO5T:scr
B
YPD3
YPD2
YPD1
RSC58
209000
210000
RSC58 RT-qPCR
sense
antisense
80
40
C
wt
control
PHO5T
PHO5T:scr
kD
Rsc58
100
75
α-GFP
Pgk1
50
α-Pgk1
Rsc58 abundance (normalized to Pgk1)
1,4
0,7

Figure S1. qRT-PCRs and Western blots of diploid *IME4* and *RSC58*, related to Figure 1. (**A**) Sense RNA abundances in the diploid strains with *IME4*-sfGFP (or $PHO5_T$ or $PHO5_{T:scr}$) were analysed by qRT-PCR. Values are normalized to *ACT1* and the control (sfGFP only) was set to 1. Error bars indicate standard deviation of 3 replicates. Antisense transcripts were not detectable as they could not be distinguished from –RT controls. (**B**) Transcript abundances around the *RSC58* locus from the tiling array data are shown. Watson and Crick strands are at the top and the bottom, respectively. Three biological replicates in YPD medium are depicted. Darker blue color indicates higher hybridization signal. On the right RNA levels are shown, normalized to *ACT1* as determined by qRT-PCR for *RSC58*. Black: sense levels, gray: antisense levels. Error bars indicate standard deviation of 3 replicates. (**C**) Rsc58 protein abundance in the indicated constructs was determined by Western blotting with anti-GFP antibodies. The quantification of the normalized to Pgk1 band intensities for 3 replicates is shown on the right, error bars indicate standard deviations of 3 replicates.

Figure S2

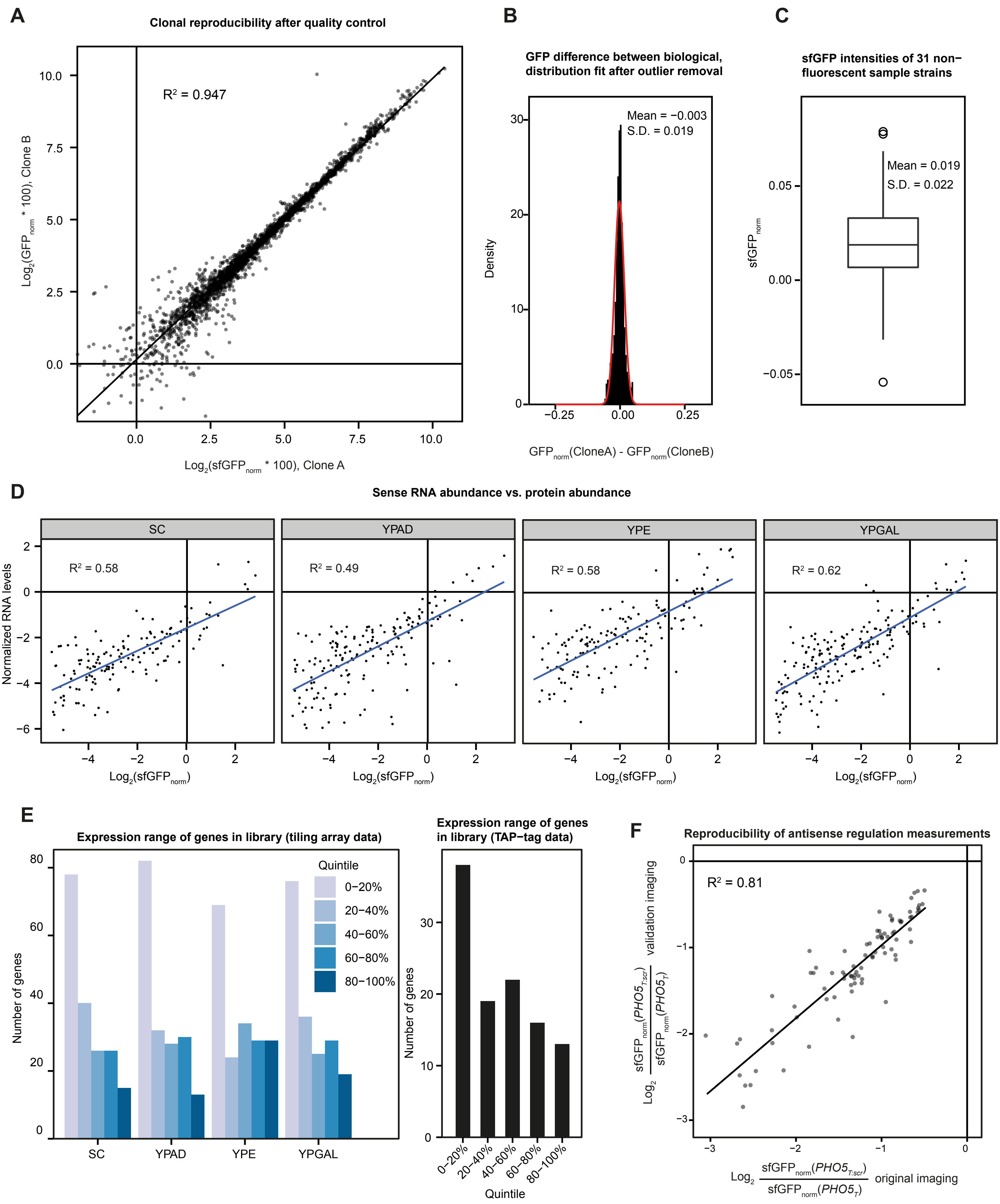
A
Clonal reproducibility after quality control
R² = 0.947
Log2(GFPnorm * 100), Clone B
Log2(sfGFPnorm * 100), Clone A
B
GFP difference between biological, distribution fit after outlier removal
Mean = −0.003
S.D. = 0.019
Density
GFPnorm(CloneA) - GFPnorm(CloneB)
C
sfGFP intensities of 31 non−fluorescent sample strains
Mean = 0.019
S.D. = 0.022
sfGFPnorm
D
Sense RNA abundance vs. protein abundance
SC
YPAD
YPE
YPGAL
R² = 0.58
R² = 0.49
R² = 0.58
R² = 0.62
Normalized RNA levels
Log2(sfGFPnorm)
E
Expression range of genes in library (tiling array data)
Quintile
0−20%
20−40%
40−60%
60−80%
80−100%
Number of genes
SC
YPAD
YPE
YPGAL
Expression range of genes in library (TAP−tag data)
Number of genes
Quintile
F
Reproducibility of antisense regulation measurements
R² = 0.81
Log2 sfGFPnorm(PHO5T:scr) / sfGFPnorm(PHO5T) validation imaging
Log2 sfGFPnorm(PHO5T:scr) / sfGFPnorm(PHO5T) original imaging

Figure S2. Quality control statistics of high throughput fluorescence microscopy and Northern blots, related to Figure 2. (**A**) Plot of the $Log_2(sfGFP_{norm} * 100)$ values of all pairs of biological replicates (clones) of the library after quality control with Pearson's R^2 indicated. (**B**) Distribution of the differences of normalized GFP intensities $sfGFP_{norm}$ of clones A and B before quality control but after outlier removal. The red line indicates a normal distribution with the mean and standard deviation values of the distribution as annotated in the plot. (**C**) Distribution of the normalized GFP intensities $sfGFP_{norm}$ (normalized to the fluorescence of the control cells without sfGFP) of 31 strains known to be non-fluorescent due to sfGFP frameshift mutations. The mean and standard deviation of this distribution was used to define thresholds for calling a gene expressed as explained in the Supplemental Experimental Procedures section below. (**D**) Normalized RNA levels from our tiling array data were plotted against protein expression values values obtained from microscopy. Straight lines are linear fits with the corresponding R^2 values indicated in the plot. (**E**) Left panel: using tiling array data, the number of genes in the antisense library falling in the respective quintile was plotted for every growth condition. Right panel: Using TAP-tag protein expression data obtained by (Ghaemmaghami et al., 2003) the number of genes in the antisense library falling in the respective quintile was plotted. Only values in YPAD were considered as the TAP-tag data were also obtained in that medium. The counts are lower than in the left panel because not for all genes in the library protein expression values were available. (**F**) 33 of the genes found to be regulated by antisense ('original imaging') were reimaged ('validation imaging'). For the conditions where those genes were found to be regulated in the original imaging, the ratios from the original imaging and the validation imaging were plotted against each other. The straight line is a linear fit with Pearson's R^2 shown in the plot.

Figure S3

A

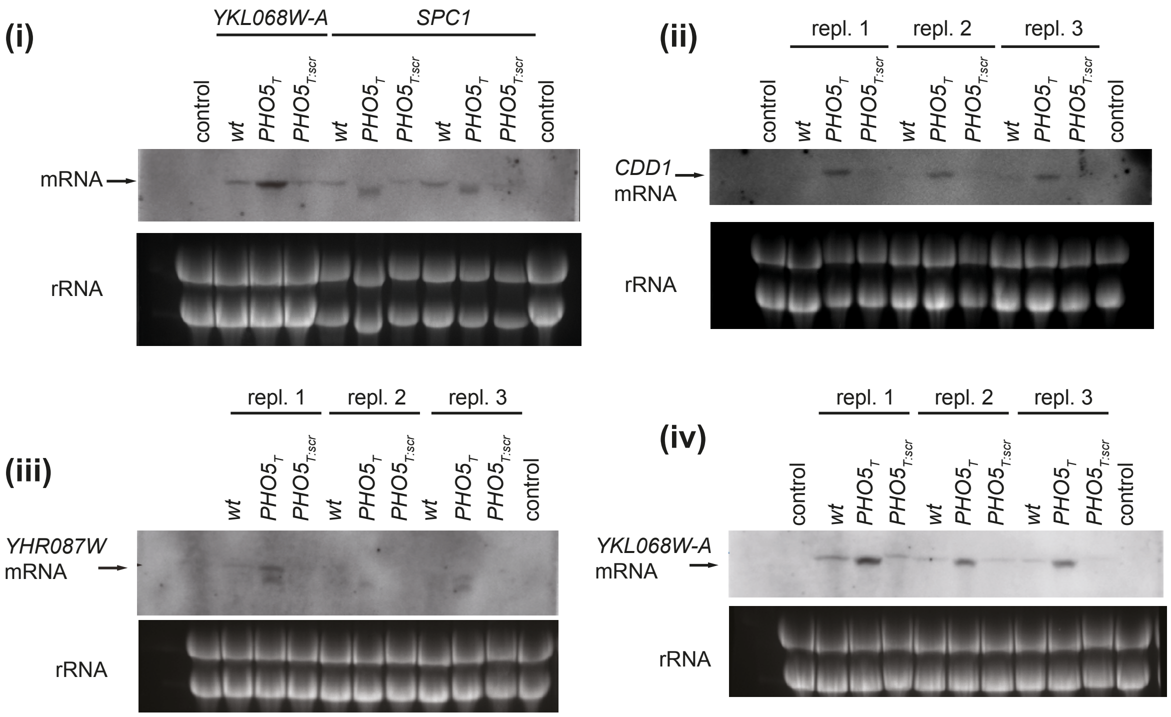
(i)
YKL068W-A
SPC1
control
wt
PHO5T
PHO5T:scr
mRNA
rRNA
(ii)
repl. 1
repl. 2
repl. 3
CDD1 mRNA
(iii)
YHR087W mRNA
(iv)
YKL068W-A mRNA

Figure S3. Northern blots against mRNA of selected antisense-regulated genes, related to Figure 2. (**A**) Total RNA from up to three independent clones of each construct of the selected library genes was extracted and separated on the gels, followed by transfer to nylon membranes. The mRNAs of the indicated genes were detected using a DIG-labelled probe annealing in the sfGFP region of the transcripts. Ribosomal RNA staining by ethidium bromide was used as a loading control.

Figure S4

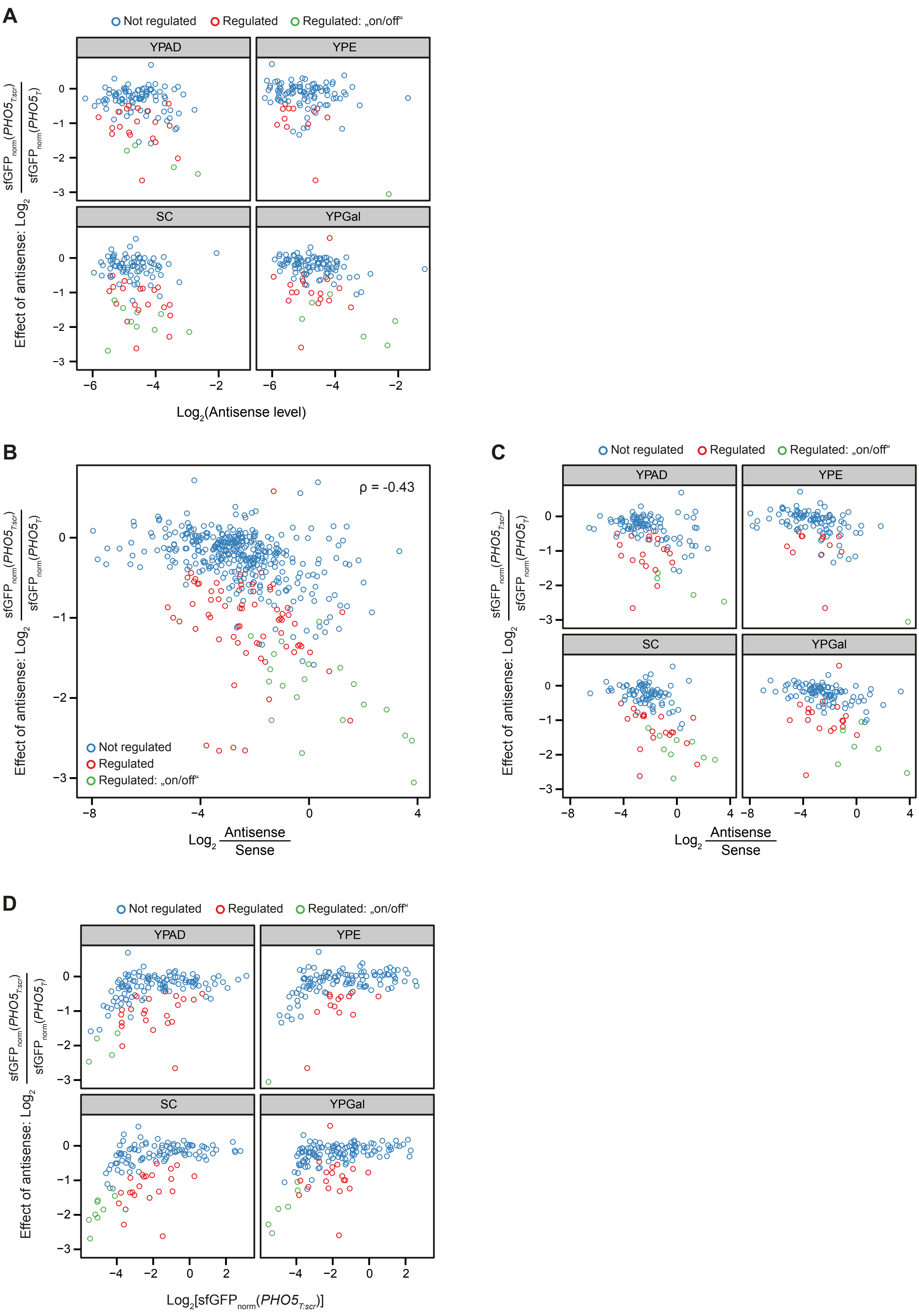

A
Not regulated
Regulated
Regulated: „on/off“
YPAD
YPE
SC
YPGal
Effect of antisense: Log2 sfGFPnorm(PHO5T:scr) / sfGFPnorm(PHO5T)
Log2(Antisense level)
B
ρ = -0.43
Not regulated
Regulated
Regulated: „on/off“
Effect of antisense: Log2 sfGFPnorm(PHO5T:scr) / sfGFPnorm(PHO5T)
Log2 Antisense / Sense
C
Not regulated
Regulated
Regulated: „on/off“
YPAD
YPE
SC
YPGal
Effect of antisense: Log2 sfGFPnorm(PHO5T:scr) / sfGFPnorm(PHO5T)
Log2 Antisense / Sense
D
Not regulated
Regulated
Regulated: „on/off“
YPAD
YPE
SC
YPGal
Effect of antisense: Log2 sfGFPnorm(PHO5T:scr) / sfGFPnorm(PHO5T)
Log2[sfGFPnorm(PHO5T:scr)]

Figure S4. Correlation of regulation by antisense and antisense/protein levels, related to Figure 3. (**A**) The effect of antisense as measured by microscopy (log2-fold change of sfGFP levels between $PHO5_{T:scr}$ and $PHO5_{T}$) was plotted against the tiling array based antisense levels for all genes with annotated antisense. The different growth conditions are plotted in separate panels. (**B**). As in (**A**) but with the $\log_2$ of antisense/sense ratios as measured by tiling arrays plotted on the x-axis. (**C**) As in (B) but with different conditions plotted in separate panels. (**D**) As in (A) but with sfGFP intensities of the $PHO5_{T:scr}$ construct plotted on the x-axis.

Figure S5

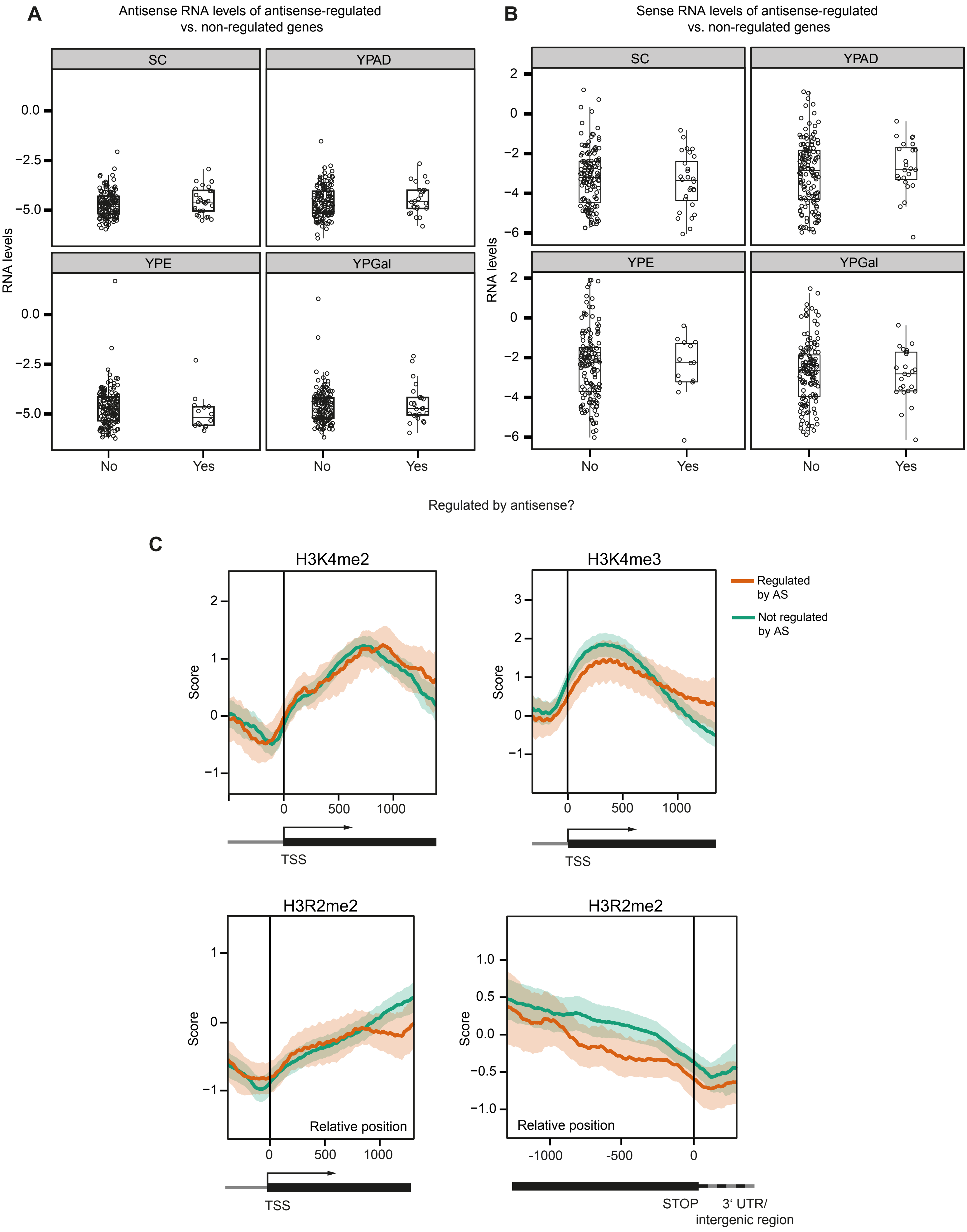

A
Antisense RNA levels of antisense-regulated vs. non-regulated genes
SC
YPAD
YPE
YPGal
RNA levels
0.0
−2.5
−5.0
No
Yes
B
Sense RNA levels of antisense-regulated vs. non-regulated genes
2
0
−2
−4
−6
Regulated by antisense?
C
H3K4me2
H3K4me3
Regulated by AS
Not regulated by AS
Score
TSS
500
1000
H3R2me2
Relative position
-1000
-500
STOP
3' UTR/ intergenic region

Figure S5. RNA levels of library genes and histone modification metagene plots, related to Figure 5. (**A**) Tiling array RNA expression levels of all the SUTs in the antisense library split by condition and depending on whether the corresponding sense gene was regulated by antisense in the respective condition. (**B**) Sense expression levels for the same genes shown in (A). (**C**) H3K4me2, H3K4me3 and H3R2me2 densities in antisense-regulated (red) vs. non-regulated (green) genes relative to either the TSS or the STOP codon as shown below the plots. Lines and ribbons indicate bootstrapping-based mean and 95% confidence interval estimates, respectively.

Figure S6

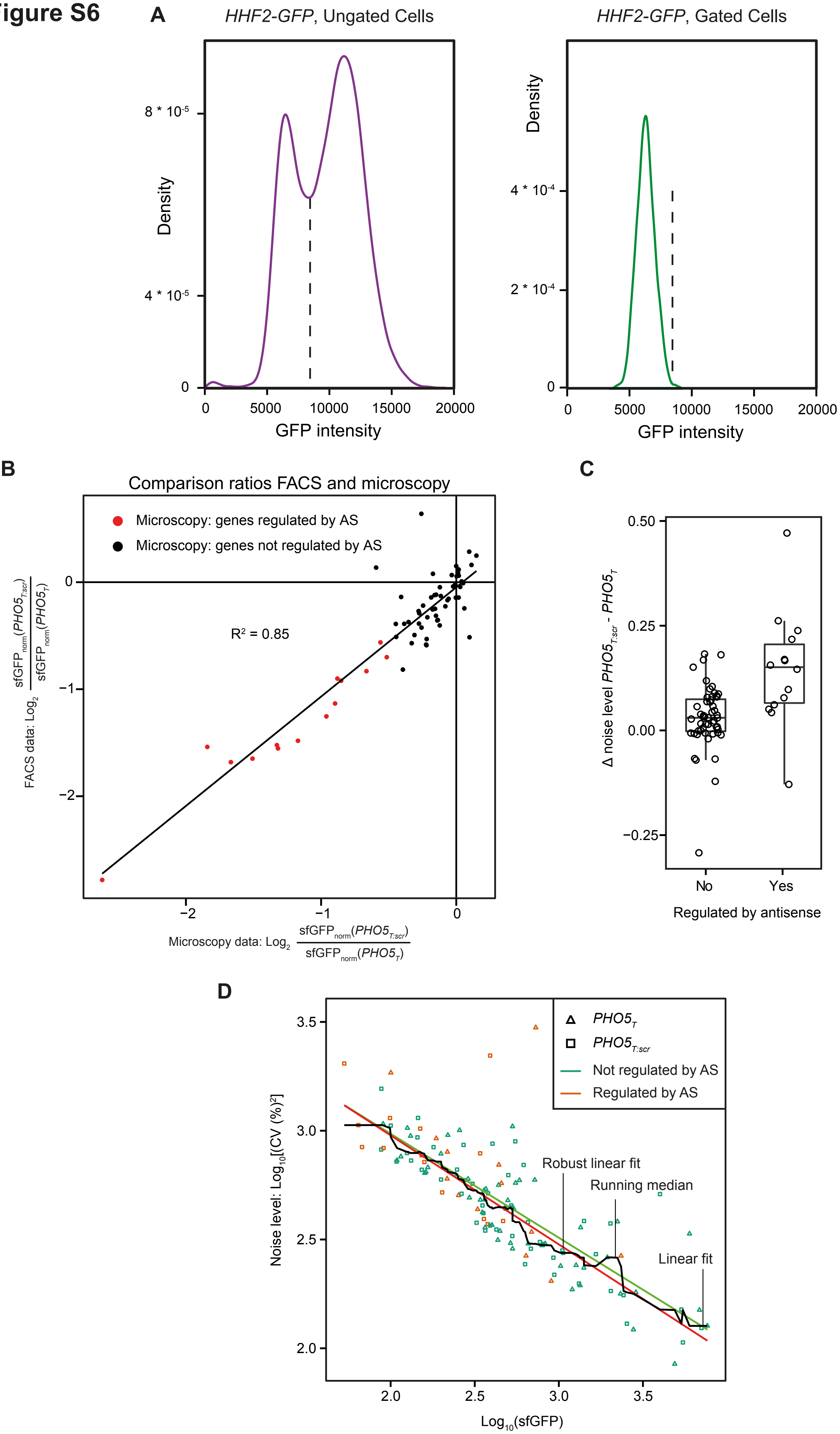
A
HHF2-GFP, Ungated Cells
HHF2-GFP, Gated Cells
Density
GFP intensity
8 * 10-5
4 * 10-5
4 * 10-4
2 * 10-4
0
5000
10000
15000
20000
B
Comparison ratios FACS and microscopy
Microscopy: genes regulated by AS
Microscopy: genes not regulated by AS
R² = 0.85
FACS data: Log2 sfGFPnorm(PHO5T:scr) / sfGFPnorm(PHO5T)
Microscopy data: Log2 sfGFPnorm(PHO5T:scr) / sfGFPnorm(PHO5T)
C
Δ noise level PHO5T:scr - PHO5T
No
Yes
Regulated by antisense
D
PHO5T
PHO5T:scr
Not regulated by AS
Regulated by AS
Robust linear fit
Running median
Linear fit
Noise level: Log10[(CV (%)²]
Log10(sfGFP)

Figure S6. Quality control of FACS results, related to Figure 6. (**A**) Distribution of GFP intensities of the histone gene *HHF2* before (left) and after (right) gating to obtain noise levels, as described in the Supplemental Experimental Procedures. (**B**) Log2 value sof the ratios of $PHO5_{T:scr}$ over $PHO5_{T}$ obtained either by FACS (y-axis) or microscopy (x-axis) were plotted against each other. Red color indicates that the gene was found to be regulated by antisense in microscopy. The straight line indicates a linear fit with (Pearson's $R^2 = 0.85$). The underlying data are from the SC growth condition. (C) A direct comparison of the gene-wise differences in noise levels (in units of $Log_{10}[CV\ (\%)^2]$, see Figure 6A) of $PHO5_{T:scr}$ minus $PHO5_{T}$ constructs. (**D**) In the original study by Churchman et al., a running median (black line) was used to fit the correlation between $Log_{10}(CV^2)$ and $Log_{10}(GFP)$. Due to the lower number of genes in our study, we found a robust linear fit to be less noisy (red line) and be less affected by outliers than a conventional linear fit (green line). Importantly, the outcome of the statistical test as shown in Figure 6B was not affected by the fitting procedure (data not shown).

Supplemental Tables

Table S1. Oligos used for antisense library construction, related to Figure 1.

This table is provided as a separate Excel file.

Table S2. Microscopy measurement values and quality control results of microscopy, related to Figure 2.

This table is provided as a separate Excel file.

Table S3. Quality control statistics, related to Figure 2.

Result of quality control	Number of wells	Percentage
**OK**	4217	93.5%
**Dead GFP**	212	4.7%
**QC fail**	83	1.8%
	**4512 total**<br>(4 conditions * 3 constructs * 2 clones * 188 genes)	100%

Table S4. Antisense regulated genes, related to Figure 2.

Growth condition	TSS overlap	No TSS overlap	No annotated antisense
YPAD	*CDD1, COX5B, FCY21, IME4, IML3, MCH5, MSS18, MTH1, OPT1, PTH1, SPC1, YHR022C-A, YHR087W, YKL068W-A, YNR004W, YPL162C, YTP1*	*CHS7, CTR1, PAN6, SHE9, SUR1, YHR112C*	*MRPL23, RCR2, YLR108C*
YPE	*CDD1, COX5B, IME4, NCA3, SPC1, YDR185C, YHR087W, YKL068W-A, YPL162C*	*ELO1, PAN6, SUR1, YGR203W*	*RCR2*
SC	*ARO10, CDD1, COX5B, FCY21, IME4, MBR1, MIG2, NCA3, OPT1, PTH1, SPC1, YDR185C, YGP1, YHR022C-A, YHR087W, YIL089W, YKL068W-A, YLR091W, YNR004W, YPL162C, YTP1*	*CTR1, MEP1, PAN6, SHE9, SUR1, YHR112C, YOR011W-A*	*MRPL23, RCR2, YLR108C*
YPGal	*CDD1, COX5B, FCY21, FET4, IME4, MCH5, MSS18, NCA3, PTH1, SPC1, YDR185C, YHR022C-A, YHR087W, YIL089W, YKL068W-A, YLR091W, YNR004W, YPL162C, YTP1*	*AMS1, JEN1, PAN6, SUR1, YHR112C*	*RCR2*

XY = Genes with possible switching behavior (PHO5$_{T:scr}$ values below expression threshold) in the respective condition.

Table S5. Tested histone modifications, related to Figure 5.

Modification	5’ ends	3’ ends	Reference
Nucleosome density	=	=	(Kirmizis et al., 2007)
H3K4me1	=	=	(Kirmizis et al., 2007)
H3K4me2	=	+	(Kirmizis et al., 2007)
H3K4me3	=	+	(Kirmizis et al., 2007)
H3K36me3	=	=	(Kirmizis et al., 2009)
H3K79me3	=	=	(Kirmizis et al., 2009)
H3K4ac	=	=	(Guillemette et al., 2011)
H3K9ac	=	=	(Pokholok et al., 2005)
H3K14ac	=	=	(Pokholok et al., 2005)
H4ac	=	=	(Pokholok et al., 2005)
H3R2me1	=	=	(Kirmizis et al., 2009)
H3R2me2	=	-	(Kirmizis et al., 2007)
Spt15	=	=	(Venters and Pugh, 2008)
Sua7	=	=	(Venters and Pugh, 2008)
Ser2-P	=	=	(Kim et al., 2010)
Ser5-P	=	=	(Kim et al., 2010)
Ser7-P	=	=	(Kim et al., 2010)

Supplemental Experimental Procedures

Yeast growth media. YPAD, YPGal and YPE were made by mixing 10 g/l peptone (BD), 10 g/l yeast extract (BD) and 0.05 g/l adenine (Sigma) and adding 2% of carbon source (glucose/galactose/ethanol, 20 g, 20 g and 20 ml, respectively). SC medium was made as previously described (Sherman, 2002).

Yeast strains. The background strain for construction of the antisense library was YMaM330: *MATα, can1Δ::STE2pr-his5 lyp1Δ::STE3pr-LEU2, his3Δ1 ura3Δ0 met15Δ0, leu2Δ0::GAL1pr-I-SCEI-natNT2* (Khmelinskii et al., 2011). The mCherry containing strain used for crossing was YDB1: *leu2Δ0 met15Δ0 ura3Δ0 his3Δ1::pGPD-mCherry*. The diploid *IME4* strains were obtained by crossing the looped-out library strains with YDB1, resulting in either *IME4-sfGFP/IME4*; *IME4-sfGFP-*$PHO5_T$*/IME4* or *IME4-sfGFP-*$PHO5_{T:scr}$*/ IME4.*

Plasmids. pFA6a is an *E. coli* plasmid with an *ampR* cassette (Wach et al., 1994). For seamless tagging with sfGFP plasmid pMaM175 was used (pFA6a-S3-sfGFP-SceIsite-S.Parad.Tcyc1-ScURA3-SceIsite-sfGFPΔN–S2). For tagging with *sfGFP-*$PHO5_T$ plasmid pMaM201 was used (pFA6a-S3-sfGFP-SceIsite-S.Parad.Tcyc1-ScURA3-SceIsite-sfGFPΔN–Tpho5-S2). For tagging with $PHO5_{T:scr}$ plasmid pMaM203 was used (pFA6a-S3-sfGFP-SceIsite-S.Parad.Tcyc1-ScURA3 -SceIsite-sfGFPΔN–Tpho5scr-S2). For integration of mCherry pDB2 (pRS303H with pGPD-mCherry) was used. All plasmids are available upon request.

Selection of genes for tagging. Genes with antisense SUT transcription were selected based on annotations from previous tiling array data (Xu et al., 2009). From all genes with antisense transcripts starting downstream of the STOP codon we randomly selected 81 ORFs whose antisense transcripts were annotated to overlap the TSS (including *IME4*) and 81 genes where this was not the case. As a third group, we added 26 ORFs without annotated antisense. Genes were C-terminally tagged using a cassette that allows for seamless tagging in the YMaM330 background (Khmelinskii et al., 2011).

Seamless tagging procedure. For C-terminal tagging of the selected genes 188 pairs of standard S2/S3 gene-specific oligonucleotide primers were synthesised, each of which contained 55 bp homology to the target locus to allow in-frame fusion of the sfGFP (Janke et al., 2004). Plasmid pMaM175 contained a seamless tagging cassette for tagging with sfGFP without any additional terminator sequence. The cassette was flanked by S2/S3 primer binding sites and contained two sfGFPs: one full sequence and an N-terminal truncation for subsequent homologous recombination. In between, there was a *URA3* marker surrounded by two I-SceI endonuclease sites. The order of genetic elements was thus pFA6a-S2site-sfGFP-SceI-URA3-SceI-ΔN-sfGFP-S3site. To create $PHO5_T$ and $PHO5_{T:scr}$ constructs, the $PHO5_T$ or $PHO5_{T:scr}$ sequence were inserted in antisense direction to the cassette described above between the ΔN-sfGFP and S3 site, resulting in plasmids pMaM201 and pMaM203. The haploid parent yeast strain YMaM330 was transformed with the PCR products generated from one of the cassettes using gene-specific primers. The clones were selected on SC -uracil medium (synthetic complete media with 2% glucose lacking uracil). Insertion of the cassette by homologous recombination was verified by PCR of samples from individual colonies using ORF specific forward primers and the generic reverse primer

sfGFPrev_alt2. Four positive clones of each strain were frozen and two were taken for further experiments. A list of all the ORFs with S2/S3 and validation primers can be found in Table S1.

To later distinguish sample from autofluorescent control cells in microscopy, the antisense library was crossed to YDB1, which contains a heterologous copy of mCherry, and subsequently selected for haploids containing both the tagged genes and mCherry using the SGA protocol (Tong and Boone, 2007). Two clones of the antisense library strains were then subjected to the loop out of the *URA3* marker.

Loop-out of the marker is based upon conditional expression of the I-SceI endonuclease on Galactose/Raffinose plates and subsequent double strand break repair via homologous recombination (Khmelinskii et al., PloS One 2011). After stimulating the loop-out with galactose cells with looped out marker are selected for by counterselection of the *URA3* marker on SC medium containing 5-fluorootic acid. The final seamlessly tagged strains were used in all further experiments.

Fluorescence microscopy.

Cells were inoculated to 96-well plates in their respective media and grown to saturation over night at 30 °C. For growth in YPE the cells were additionally pre-cultured in YPE after saturation in YPAD. Saturated cultures were diluted to OD_{600} = 0.025 into fresh medium and mixed 1:1 with sfGFP-negative control cells for background subtraction and signal normalization on a well-by-well basis. Populations were distinguished by using an mCherry cassette contained in the antisense library. Cultures were grown to mid log phase for 7-8 hours. 70 µl of the cultures were transferred to 96-well PCR plates and fixed with 70 µl of freshly prepared 8% paraformaldehyde in 1x PBS for 5 minutes. The cells were washed 5 times using 1x PBS and then resuspended in 120 µl of 1x PBS. 20 µl of the cell suspension were used for microscopy in 384-well glass-bottom microscopy plates (Matriplate, GE Healthcare) pretreated with concanavalin A (100 µg/ml, Sigma). Imaging was performed on a Nikon Ti-E wide field epifluorescence screening microscope with a 60x ApoTIRF oil-immersed objective (1.49 NA, Nikon), an LED light engine (SpectraX, Lumencor), a 2048x2048 pixel (6.5 µm) sCMOS camera (Flash4, Hamamatsu) and an autofocus system (Perfect Focus System, Nikon). Nine images for each of mCherry, sfGFP and brightfield channels were recorded per position using three different exposure times for sfGFP. Wells to correct for shading artifacts were set up for every plate.

Image postprocessing and cell segmentation. All fluorescence images were flat-field corrected to correct for uneven illumination across the field of view. Before flat-field correction, the camera offset, which is introduced by manufacturers to prevent negative values, was subtracted from all images (including reference images). Next, flat-field correcation was done on a plate-by-plate basis by dividing every image by a reference image derived from a well containing a diluted recombinant mCherry-sfGFP fusion protein. To obtain the reference image, all images from the corresponding well were median projected, smoothed by a combination of median, mean and Gaussian filters and divided by the image median. Next, cells were segmented by processing out of focus brightfield images using a modified version of the pipeline published by Buggenthin and colleagues (Buggenthin et al., 2013). The obtained images were further processed using custom ImageJ scripts to generate cell masks of the appropriate size. These scripts minimized the number of masks derived from out of focus cells and excluded all masks with an area <5 µm^2 or >15 µm^2 or with a circularity <0.85. Sample cells and autofluorescent control cells were co-cultured and distinguished by a heterologous mCherry cassette in the

sample cells. Masks were therefore split into a sample and an autofluorescent control cell population using image-based thresholding in the mCherry channel. Next, masks were applied to the flat-field corrected GFP images resulting for every cell in mean fluorescence intensity values and morphological parameters.

Calculation of normalized sfGFP values and determining a gene expression threshold. Background subtraction and normalization were done for every well independently to control for well-to-well and plate-to-plate variability. For every well, background subtraction was done by subtracting the median sfGFP intensity of the autofluorescent background cell population from the sfGFP intensity of every sample cell. The resulting background subtracted sfGFP intensities of the sample cells were then normalized by dividing by the median sfGFP intensity of the autofluorescent background cell population. Thus, the normalized sfGFP intensity for every sample cell is calculated by:

$$sfGFP_{norm} = \frac{sfGFP - median(sfGFP_{bg})}{median(sfGFP_{bg})}$$

where $sfGFP_{norm}$ is the normalized sfGFP intensity of a sample cell and *sfGFP* denotes its raw sfGFP signal intensity. median($sfGFP_{bg}$) is the median sfGFP signal intensity of the autofluorescent background cell population. The obtained median of the normalized $sfGFP_{norm}$ intensities of the sample cell population represented our preliminary gene expression values.

To find out which strains expressed their tagged genes above background, we determined the normalized sfGFP intensity of 31 non-functional sfGFP strains in the library where both visual inspection and Sanger sequencing confirmed the absence of functional sfGFP as compared to the second clone of the strain. We considered the preliminary expression values of those strains as our background (Figure S2C). The resulting mean (0.019) was subtracted from preliminary expression values to obtain final gene expression values normalized to the autofluorescence of non-fluorescent cells on a well-by-well basis (termed $sfGFP_{norm}$). Subsequently, we defined a strain to be expressed above background if the final gene expression value was higher than 3 standard deviations of the $sfGFP_{norm}$ values of the non-functional sfGFP strains (resulting in a threshold of 0.065, see Figure S2C). To prevent ratios from approaching infinity, gene expression values found to be lower than 1 standard deviation of the non-functional strains were set to 1 standard deviation of those strains.

Quality control of microscopy data. First, uniformity of growth between sample and autofluorescent control cells was determined by checking the differences in the median areas between the two populations. We ensured that these values were centered around 0 with only minor deviations (< 0.5 μm^2, data not shown). Three sfGFP exposure times, 50 ms, 300 ms, and 1000 ms were applied during imaging. Suitable exposure times were chosen independently for each well by taking the highest possible exposure time where images were not overexposed. Only two genes, *FBA1* and *PDC1* were overexposed at all exposure times and excluded from further analysis. Proper separation of mCherry negative and mCherry positive populations (see Image postprocessing and cell segmentation section above) was checked by requiring that median mCherry intensities differed by at least four-fold between the two populations and by performing occasional visual inspection. Wells failing to meet these criteria were excluded from further analysis. Wells were also excluded from the analysis if the number of cells in any of the two populations was less than 100 as we found medians obtained from such population sizes to be

less well reproducible (data not shown). However, cell numbers were in general much higher with the median cell number per well being 950 for every population and the lower decile at 357 cells.
Next, we identified non-fluorescent strains among the expressed strains. These are a result of sequence mutations introduced during the library construction which are not detectable by colony PCR and typically result in one of the two biological replicates (clones) lacking fluorescence. To identify such cases, we first determined how much clones differed on average by fitting a normal distribution to the distribution of interclonal gene expression differences (Figure S2B). If two clones differed more than expected from this distribution and were more than 50% different from each other, the clone with a weaker expression was flagged. If a flagged clone was found to be consistently not expressed under any of the growth conditions, it was considered bad and excluded from further analysis, otherwise it was excluded only in the condition were quality control was not passed. A table containing the quality control results for the whole microscopy run can be found in Table S2. Summary statistics of quality control and reproducibility of our dataset after quality control can be found in Table S3. Subsequent Sanger sequencing revealed that >90% of the excluded clones showed frameshift mutations in the regions responsible for homologous recombination of the tagging cassette (data not shown).

Determining antisense-regulated genes. To determine genes regulated by antisense in a particular growth condition, we calculated p-values for the log2-fold changes of the ratios between the $PHO5_{T:scr}$ and the $PHO5_{T}$ strains using a linear modelling approach as implemented in the limma package of Bioconductor (Ritchie et al., 2015; Smyth, 2004). Multiple testing correction was done using the method by Benjamini and Hochberg (Benjamini and Hochberg, 1995). Hits were required to have p value of <0.01 and to differ more than 4 standard deviations of the distribution of interclonal differences (Figure S2B). Genes were $PHO5_{T:scr}$ differed more than 50% from the control (sfGFP only) strains were excluded prior to this analysis because we reasoned that in those cases the introduction of additional nucleotides at the 3' end of the gene led to a major impairment of native gene function. We also did not consider genes that under the respective condition fell below the expression in both the $PHO5_{T}$ and the $PHO5_{T:scr}$ constructs.

Determining condition-specific regulation. All genes that were regulated in at least one condition were selected as candidates for condition-specific regulation. Cases where $\log_2(PHO5_{T:scr}/PHO5_{T})$ differed significantly were determined using limma, setting the significance cutoff to p = 0.01 and requiring that $\log_2$-fold changes differ by at least 1 and/or approach 0 in one of the tested conditions. To call condition-specific regulation, we furthermore stipulated that (i) both $PHO5_{T:scr}$ and $PHO5_{T}$ be above the expression threshold because in those cases obtained $\log_2(PHO5_{T:scr}/PHO5_{T})$ are better reproducible and (ii) that the gene be significantly regulated (see above) in not all of the conditions.

Metagene analyses. For NET-seq metagene analysis (Figure 4C) the genome coordinates of 750 bp regions were retrieved for every gene, ranging either from -300 bp to +450 bp of the transcript start sites (TSSs) or from -450 bp to + 300 bp of the STOP codon (TSS and STOP being coordinates 0). TSS positions were based on tiling array data and are available upon request. Next, NET-seq read numbers at the respective regions were retrieved for every gene using the dataset from Churchman and colleagues (Churchman and Weissman, 2011). Genes were then first grouped into antisense-regulated and non-regulated genes and those groups were further

split up by their original classification, i.e. depending on whether antisense transcripts were annotated and if so, whether they overlapped the TSS or not. Only genes tested for regulation (see section "Determining antisense-regulated genes" above) were considered and antisense regulation was based on data obtained in YPAD as this corresponds to the condition used by Churchman et al. NET-seq read numbers were then averaged for every gene in bins of 10 nucleotides and the median read numbers of those bins determined for every group. Finally, the resulting traces were smoothed using a running mean of 10 bins. For metagene traces of histone modifications (Figures 5A and B and Figure S5C) the approach was similar but with changes in parameter settings as follows: Genes that did not have an annotated antisense transcript were not considered. The bin size was set to 1 nucleotide and the window size was set to 150 bins. Mean values and confidence intervals at a 95% level were calculated using nonparametric bootstrapping as implemented in the R Hmisc package, version 3.17-0. For a description of the data sources used see the paragraph "Data sources" below.

Data sources. Transcript boundaries and ncRNA annotations were based on tiling array data sets obtained in our laboratory as described above. Gene sequences, gene models and annotations were derived from Bioconductor packages using genome version sacCer3 from UCSC (2011). NET-seq data were obtained from (Churchman and Weissman, 2011). ChIP-chip data on H3K4me3 and H3R2me2 were obtained from (Kirmizis et al., 2007). ChIP-chip data on H3K36me3, H3K79me3, H3R2me1 data were obtained from (Kirmizis et al., 2009). ChIP-chip on H3K4ac was obtained from (Guillemette et al., 2011). Data on H3K9ac, H3K14ac and H4ac was obtained from (Pokholok et al., 2005). Data on Sua7 and Spt15 occupancy was obtained from (Venters and Pugh, 2008). PolII CTD phosphorylation patterns were obtained from (Kim et al., 2010). Binding sites of RNA-binding proteins were obtained from (Hogan et al., 2008). For identification of known DNA sequence motifs, the Bioconductor MotifDb package was used (Shannon, 2015). The motif database was cleared of redundant entries by preferring ScerTF over JASPAR over UniPROBE derived motifs. To discover *de novo* motifs that are enriched in hits vs. non-hits or vice versa, the tool DREME from the MEME suite, version 4.10.2 (Bailey et al., 2009).

Flow cytometry: data processing and noise estimation. Strains with intact sfGFP fluorescence (using the information obtained by microscopy) were obtained from our antisense library by streaking to single colonies. Two independent colonies (clones) were then grown in 96-well plates (8 plates in total). We recorded 100,000 events per well of mid-log phase cells growing in SC using a flow cytometer equipped with a high throughput stage (BD FACSCanto RUO HTS). Subsequently, single (G1) cells were selected by plotting the side scatter width of all cells and selecting for the smaller population (Matthias Meurer, personal communication). Next, a circular gate was defined as described in (Newman et al., 2006) that results in a homogeneous yeast cell population where the influence of extrinsic noise is minimized (leaving roughly 5000 – 10000 cells per well using a radius of 5000). Briefly, the medians of forward and side scatter widths are taken as the center of a circular gate and the radius of this gate is reduced in small steps. Initially, the coefficient of variation (CV) drops but at some point stabilizes at a certain value that is largely dominated by intrinsic noise (Newman et al., 2006). sfGFP values were normalized plate-wise by dividing by fluorescent beads added to all the wells of the first column of every plate. A threshold for expression was determined by measuring a non-fluorescent strain for every plate and calculating a 99.9% confidence interval. Strains with sfGFP values below this threshold were

not considered. For reproducibility, we stipulated that sfGFP and CV values be within a 15% range of each other. We validated the strategy by comparing the CV values obtained from a set of reference strains used in Newman et al., 2006. Similar values were obtained (data not shown). As an additional control, the GFP intensity profile of a *HHF2-GFP* strain resulted after gating in a uniform population of G1 cells (Figure S6A). To account for the dependence of the CV values on protein abundance (Figure 6A) we calculated a robust linear fit to the data, using the implementation as in the MASS package in R, version 7.3-45. We found this to be a better fit than the approach using a running median in Newman's study. Subsequent calculation of the residuals to that fit enable the assignment of noise levels while accounting for changes in protein abundance. We then subtracted the residual of $PHO5_{T:scr}$ from the one in $PHO5_{T}$ for every gene separately and compared the distributions of antisense-regulated genes to non-regulated ones as previously determined by microscopy in SC medium (Figure 6B).

Statistical tests. Unless otherwise specified, two distributions were compared as follows: First, the underlying data was tested for normality using the Shapiro-Wilk test. Next, in the case of normal distribution a Student's t-test was performed, otherwise a Wilcoxon's rank sum test (all tests run as implemented in R, package stats, version 3.2.2). Multiple testing corrections were performed if adequate, using the method by Benjamini and Hochberg (Benjamini and Hochberg, 1995).

Northern blotting. Total yeast RNA was extracted from mid-log phase cultures as described in (Collart and Oliviero, 2001). Northern blotting of total RNA was performed as described (Luke et al., 2008) with some modifications. Briefly, 20 µg of total RNA were separated by electrophoresis on a formaldehyde agarose 1.2% gel. RNA was transferred to IMMOBILON NY+ charged nylon membrane (Millipore). Blots were blocked in UltraHyb hybridization buffer (Ambion) for 1h at 42 °C and hybridized for 14-16h with DIG-labelled probes (DIG-labeling kit, Roche). Blots were washed (2x SSC with 0.1% SDS; 0.5x SSC with 0.1% SDS; 0.2x SSC with 0.1% SDS), then blocked and incubated with anti-DIG antibodies (Roche). The signals were detected using CDP-star reagent (Roche) and a chemiluminescent imager. Ribosomal RNA stained with ethidium bromide was used as a loading control.

RT-qPCR primer sequences.
sfGFP fwd: TGTTAGAGGTGAGGGCGAAG
sfGFP rev: TACTAGGGTTGGCCAAGGAA
actin fwd: TGATGACTTGACCATCTGGAAGTTCGTAGG
actin rev: GGACTTCGAACAAGAAATGCAAACCGC
sfGFPrev_alt2: CTCTTCACCCTTGGACATAGC

Supplemental References

Benjamini, Y., Hochberg, Y., 1995. Controlling the False Discovery Rate: A Practical and Powerful Approach to Multiple Testing. J. R. Stat. Soc. Ser. B Methodol. 57, 289–300.

Buggenthin, F., Marr, C., Schwarzfischer, M., Hoppe, P.S., Hilsenbeck, O., Schroeder, T., Theis, F.J., 2013. An automatic method for robust and fast cell detection in bright field images from high-throughput microscopy. BMC Bioinformatics 14, 297.

Guillemette, B., Drogaris, P., Lin, H.-H.S., Armstrong, H., Hiragami-Hamada, K., Imhof, A., Bonneil, É., Thibault, P., Verreault, A., Festenstein, R.J., 2011. H3 Lysine 4 Is Acetylated at Active Gene Promoters and Is Regulated by H3 Lysine 4 Methylation. PLoS Genet 7, e1001354. doi:10.1371/journal.pgen.1001354

Janke, C., Magiera, M.M., Rathfelder, N., Taxis, C., Reber, S., Maekawa, H., Moreno-Borchart, A., Doenges, G., Schwob, E., Schiebel, E., Knop, M., 2004. A versatile toolbox for PCR-based tagging of yeast genes: new fluorescent proteins, more markers and promoter substitution cassettes. Yeast 21, 947–962. doi:10.1002/yea.1142

Kim, H., Erickson, B., Luo, W., Seward, D., Graber, J.H., Pollock, D.D., Megee, P.C., Bentley, D.L., 2010. Gene-specific RNA polymerase II phosphorylation and the CTD code. Nat. Struct. Mol. Biol. 17, 1279–1286. doi:10.1038/nsmb.1913

Kirmizis, A., Santos-Rosa, H., Penkett, C.J., Singer, M.A., Green, R.D., Kouzarides, T., 2009. Distinct transcriptional outputs associated with mono- and dimethylated histone H3 arginine 2. Nat. Struct. Mol. Biol. 16, 449–451. doi:10.1038/nsmb.1569

Luke, B., Panza, A., Redon, S., Iglesias, N., Li, Z., Lingner, J., 2008. The Rat1p 5′ to 3′ Exonuclease Degrades Telomeric Repeat-Containing RNA and Promotes Telomere Elongation in Saccharomyces cerevisiae. Mol. Cell 32, 465–477. doi:10.1016/j.molcel.2008.10.019

Ritchie, M.E., Phipson, B., Wu, D., Hu, Y., Law, C.W., Shi, W., Smyth, G.K., 2015. limma powers differential expression analyses for RNA-sequencing and microarray studies. Nucleic Acids Res. doi:10.1093/nar/gkv007

Smyth, G.K., 2004. Linear Models and Empirical Bayes Methods for Assessing Differential Expression in Microarray Experiments. Stat. Appl. Genet. Mol. Biol. 3, 1–25.

Tong, A.H.Y., Boone, C., 2007. 16 High-Throughput Strain Construction and Systematic Synthetic Lethal Screening in, in: Methods in Microbiology. Elsevier, pp. 369–707.

Venters, B.J., Pugh, B.F., 2008. A canonical promoter organization of the transcription machinery and its regulators in the Saccharomyces genome. Genome Res. 19, 360–371. doi:10.1101/gr.084970.108

Wach, A., Brachat, A., Pöhlmann, R., Philippsen, P., 1994. New heterologous modules for classical or PCR-based gene disruptions in Saccharomyces cerevisiae. Yeast 10, 1793–1808. doi:10.1002/yea.320101310
