# Supplementary material for: Protein Abundance Control by Non-coding Antisense Transcription
Source: Cell Rep. 2016 Jun 9;15(12):2625–36. doi: 10.1016/j.celrep.2016.05.043 (PMC4920891; doi:10.1016/j.celrep.2016.05.043)
Supplement: Document S2. Article plus Supplemental Information [file mmc4.pdf]

Cell Reports

Article

Protein Abundance Control by Non-coding Antisense Transcription

Graphical Abstract

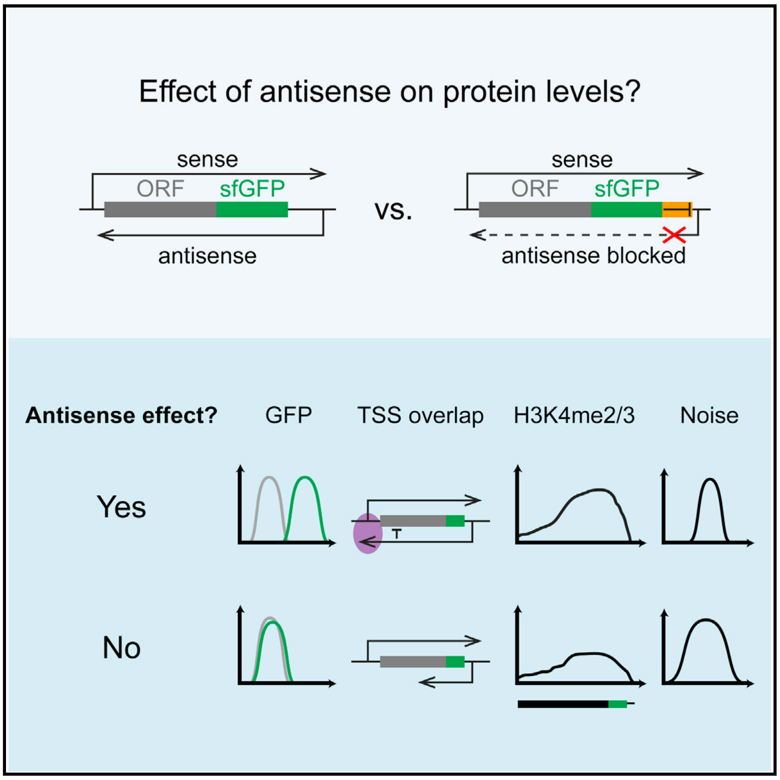

Authors

Florian Huber, Daria Bunina, Ishaan Gupta, ..., Patrick Theer, Lars M. Steinmetz, Michael Knop

Correspondence

m.knop@zmbh.uni-heidelberg.de

In Brief

Huber et al. conduct a systematic study of the function of antisense RNAs in yeast by selectively suppressing the antisense transcripts of 188 GFP-tagged genes and quantifying the resulting changes in protein expression levels. The authors use this information to identify features that distinguish functional from non-functional antisense RNAs.

Highlights

- The impact of antisense transcription on protein levels is measured for 188 genes
- Antisense has mostly weak suppressive effects on ~25% of the genes
- Regulation by antisense correlates with promoter overlap and H3K4 methylation
- Antisense-regulated genes have lower than expected noise levels

Accession Numbers

E-MTAB-4731

CrossMark

Huber et al., 2016, Cell Reports *15*, 2625–2636
June 21, 2016 © 2016 The Author(s).
http://dx.doi.org/10.1016/j.celrep.2016.05.043

CellPress

OPEN ACCESS
CellPress

Protein Abundance Control by Non-coding Antisense Transcription

Florian Huber,[1,6] Daria Bunina,[1,6] Ishaan Gupta,[2] Anton Khmelinskii,[1] Matthias Meurer,[1] Patrick Theer,[1,3] Lars M. Steinmetz,[2,4,5] and Michael Knop[1,3,*]
[1]Zentrum für Molekulare Biologie der Universität Heidelberg (ZMBH), University of Heidelberg, Im Neuenheimer Feld 282, 69120 Heidelberg, Germany
[2]Genome Biology Unit, European Molecular Biology Laboratory (EMBL), Meyerhofstraße 1, 69117 Heidelberg, Germany
[3]Deutsches Krebsforschungszentrum (DKFZ), Im Neuenheimer Feld 280, 69120 Heidelberg, Germany
[4]Stanford Genome Technology Center, Stanford University, Palo Alto, CA 94304, USA
[5]Department of Genetics, Stanford University School of Medicine, Stanford, CA 94305, USA
[6]Co-first author
*Correspondence: m.knop@zmbh.uni-heidelberg.de
http://dx.doi.org/10.1016/j.celrep.2016.05.043

SUMMARY

Stable unannotated transcripts (SUTs), some of which overlap protein-coding genes in antisense direction, are a class of non-coding RNAs. While case studies have reported important regulatory roles for several of such RNAs, their general impact on protein abundance regulation of the overlapping gene is not known. To test this, we employed seamless gene manipulation to repress antisense SUTs of 162 yeast genes by using a unidirectional transcriptional terminator and a GFP tag. We found that the mere presence of antisense SUTs was not sufficient to influence protein abundance, that observed effects of antisense SUTs correlated with sense transcript start site overlap, and that the effects were generally weak and led to reduced protein levels. Antisense regulated genes showed increased H3K4 di- and trimethylation and had slightly lower than expected noise levels. Our results suggest that the functionality of antisense RNAs has gene and condition-specific components.

INTRODUCTION

High-throughput technologies such as tiling arrays and deep sequencing enable genome-wide and strand-specific detection of RNAs and have revealed the pervasive nature of transcription in eukaryotic genomes (Bertone et al., 2004; David et al., 2006; Nagalakshmi et al., 2008), resulting in the identification of many classes of non-coding RNAs (ncRNAs). In *Saccharomyces cerevisiae,* ncRNAs typically originate from nucleosome-depleted regions (NDRs), which are frequently associated with bidirectional promoters of protein-coding genes (Neil et al., 2009; Xu et al., 2009). However, such pervasive transcription from NDRs is limited by a combination of transcriptome surveillance mechanisms such as transcription attenuation mediated by the Nrd1-Nab3-Sen1 (NNS) termination complex (Arigo et al., 2006; Schulz et al., 2013), suppression of divergent transcription via histone marks (Churchman and Weissman, 2011; Marquardt et al., 2014; Uprety et al., 2016), or rapid degradation of the resulting transcripts by the exosome (Davis and Ares, 2006; van Dijk et al., 2011; Wyers et al., 2005).

In contrast to exosome-sensitive cryptic unstable transcripts (CUTs), stable unannotated transcripts (SUTs) are readily detectable in wild-type cells, and more than 600 of such transcripts have been annotated in yeast (Xu et al., 2009). When ncRNAs are transcribed in antisense direction to an open reading frame (ORF), they are also referred to as antisense RNAs (asRNAs).

In a number of detailed studies, asRNAs were found to exert important biological functions by regulating the expression of the overlapping gene. For example, the asRNA *RME2* blocks entry into meiosis in haploid yeast cells by repressing transcription elongation of the *IME4* gene (Gelfand et al., 2011; Hongay et al., 2006). Lenstra and colleagues demonstrated that an RNA antisense to *GAL10* prevents transcriptional leakage of both *GAL10* and *GAL1*, thus modulating the responsiveness of the underlying metabolic switch (Lenstra et al., 2015). In addition, strong regulatory functions of asRNAs in yeast have been shown for several genes, including *CDC28* (Nadal-Ribelles et al., 2014), *PHO84* (Camblong et al., 2009, 2007; Castelnuovo et al., 2013), *PHO5* (Uhler et al., 2007), and *IME1* (van Werven et al., 2012), each with individual mechanistic characteristics different from RNAi, as *S. cerevisiae* lacks a functional RNAi machinery (Drinnenberg et al., 2011, 2009).

While these cases are well established, our understanding of which asRNAs serve a biological function and whether those share certain characteristics remains incomplete (Pelechano and Steinmetz, 2013). Several high-throughput transcriptome studies generated correlative data on transcript levels of sense-antisense pairs on a genome-wide scale. From these studies, a picture emerges where sense levels are anticorrelated with antisense. However, not all sense transcript levels are affected by changes in antisense transcript levels, and the anticorrelation is often weak (Alcid and Tsukiyama, 2014; Castelnuovo et al., 2014; Schulz et al., 2013; Xu et al., 2011, 2009). Importantly, others have pointed out that there is a lack of anticorrelation at the level of nascent transcription (Murray et al.,

© 2016 The Author(s).
This is an open access article under the CC BY license (http://creativecommons.org/licenses/by/4.0/).

2015). In the latter study, Murray and colleagues detected specific chromatin signatures associated with antisense transcription. From this, they hypothesized that the main biological function of asRNAs lies in affecting traits such as expression noise or gene silencing rather than affecting bulk protein abundances. Indeed, studies showing the on/off switching of sense-antisense pairs in different conditions (Lenstra et al., 2015; Nguyen et al., 2014) and suggesting effects of antisense transcription on noise levels (Xu et al., 2011) exist. However, how common such roles are among all antisense transcripts remains to be determined.

Therefore, our understanding of the general principles that govern antisense-dependent gene regulation remains incomplete (Pelechano and Steinmetz, 2013). Some of the main reasons concern methodological difficulties. First, studies that measure protein rather than RNA levels are lacking. Second, datasets typically rely on correlative data and use mutants that interfere with ncRNA stability, since direct ncRNA abrogation is difficult given the overlapping arrangement of sense-antisense pairs. Hence, the causality of sense-antisense regulation is often unclear. In addition, determining the proportion of antisense transcripts that have a biological function is difficult. Finally, it remains an open question which features are predictive of asRNA functionality and whether such features are shared among many asRNAs.

Here, we interfered with antisense transcription of 162 genes and assessed the impact of this disturbance on the level of the expressed protein using single-cell microscopy. The strategy used is based on seamless genomic manipulations (Khmelinskii et al., 2011) and a specific DNA element from the *PHO5* termination region that blocks transcription in a unidirectional manner (Irniger et al., 1991). We applied this strategy to 188 genes in *S. cerevisiae*, 162 of which had been annotated with antisense SUTs. We then assessed the resulting changes of protein levels of GFP-tagged variants of the overlapping genes by high-throughput fluorescence microscopy in four growth conditions and by using flow cytometry. This allowed us to investigate the general impact of asRNAs on protein abundance and gene expression regulation.

RESULTS

Antisense Library Construction

A previous study on yeast polyadenylation sites reported that a short sequence of ~100 bp from the 3′ intergenic region of the *PHO5* gene acts as a unidirectional terminator (Irniger et al., 1991). To explore whether such an element could be used for the specific abrogation of antisense transcription, we tailored a strategy based on seamless gene tagging (Khmelinskii et al., 2011). We inserted this fragment, termed $PHO5_T$, and a scrambled control, $PHO5_{T:scr}$, directly downstream of the stop codon of genes so that no auxiliary sequences such as marker genes were left behind after insertion and seamless marker excision. In order to measure protein levels, we simultaneously fused superfolder GFP (sfGFP; Pédelacq et al., 2006) to the C terminus of the proteins (Figures 1A and 1B). We tested this strategy using *IME4*, a gene that is suppressed by the asRNA *RME2* in haploid yeast (Gelfand et al., 2011; Hongay et al., 2006). We observed Ime4-sfGFP mRNA and protein upregulation and asRNA downregulation upon insertion of the $PHO5_T$ element, but not with the $PHO5_{T:scr}$ element or when sfGFP was inserted alone (Figure 1C). In diploid yeast, transcription of *RME2* is suppressed (Hongay et al., 2006). Accordingly, using strand-specific qRT-PCR on *IME4* in a diploid context, we could not detect asRNA. Notably, there were no significant differences in sense RNA levels between the three constructs, indicating that $PHO5_T$ and $PHO5_{T:scr}$ do not, in general, lead to changes in RNA stability or transcription rates when compared to the control (Figure S1A).

We also applied our strategy to *RSC58*, a gene with no reported asRNA. Sense RNA and protein levels did not change (Figures S1B and S1C), indicating that the insertion of $PHO5_T$ and $PHO5_{T:scr}$ did not affect transcription of this gene. Interestingly, for this gene, qRT-PCR detected low levels of an antisense transcript, which was repressed by the $PHO5_T$ element. This indicates that current SUT/CUT annotations are probably a conservative estimate dictated by the sensitivity of the specific assays used for their detection and that antisense transcription did not influence sense levels of this particular gene.

We conclude that our strategy represents a suitable approach to study the impact of antisense transcription on protein levels by specifically disrupting antisense transcription while leaving sense transcription unaffected.

The above results encouraged us to apply the $PHO5_T$ strategy to study the function of a larger number of asRNAs. To this end, we tagged approximately one-quarter of the 613 yeast ORFs that are annotated with antisense SUTs starting downstream of the STOP codon (Xu et al., 2009), using *sfGFP*-$PHO5_T$, *sfGFP*-$PHO5_{T:scr}$ or *sfGFP* alone. A total of 188 ORFs were chosen randomly from the subset of genes predicted to be not affected by the C-terminal sfGFP tag (based on previous genome-wide datasets; Gavin et al., 2006; Ghaemmaghami et al., 2003; Huh et al., 2003; Khmelinskii et al., 2014). The selected genes differ in the extent to which the asRNA overlaps the sense. For 81 genes, the annotated asRNA overlaps the transcript start site (TSS). For another 81 genes, the asRNA terminates earlier. In addition, we selected 26 genes that contain no annotated asRNA (Figure 1D; Table S1). To control for PCR errors associated with PCR targeting during library construction, we used two biological replicates of each strain for subsequent experiments (Experimental Procedures).

Analysis of the Antisense Library Using Quantitative Microscopy

To determine the effect of antisense transcription on protein abundance, we compared sfGFP intensities of the $PHO5_T$, $PHO5_{T:scr}$ and control strains in our library. For this purpose, we established and validated a high-throughput quantitative fluorescence microscopy pipeline and quantified total cellular sfGFP fluorescence in >100 cells for each of the strains using automated image acquisition and analysis (Figure 2A; Experimental Procedures). We used mid-log phase cells grown at 30°C using the four growth conditions in which the SUTs had been annotated (Xu et al., 2009): rich medium with either glucose, galactose, or ethanol (YPAD, YPGal, and YPE) and synthetic complete medium (SC). Approximately 5% of the data points were removed based on different quality control criteria (Experimental Procedures; Figures S2A–S2C; Tables S2 and

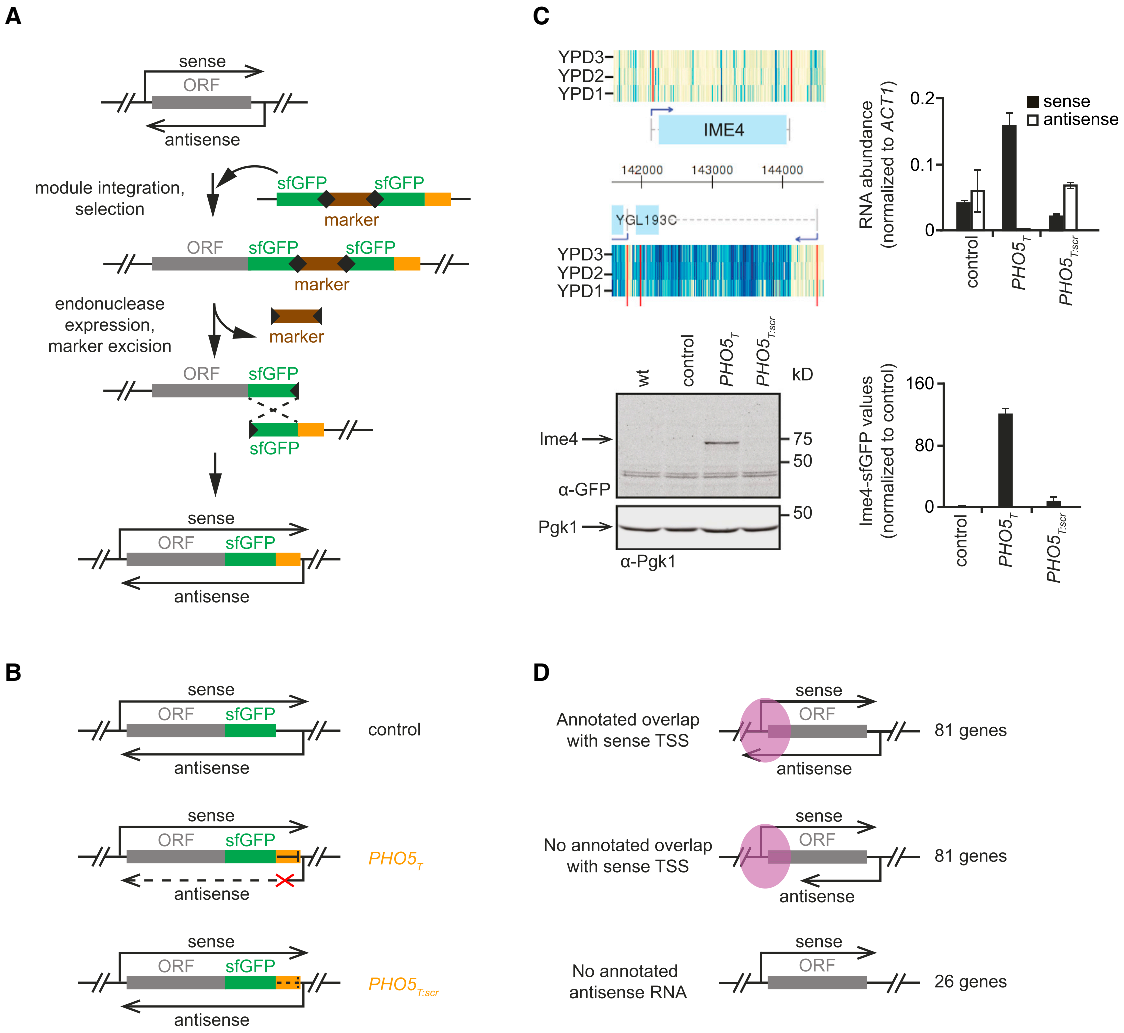

Figure 1. Antisense Deletion Library: Strategy, Library Construction, and Validation

(A) Abrogation of gene-specific asRNA transcription using seamless gene tagging. A cassette containing one full sfGFP and a ΔN-sfGFP fragment and two I-SceI sites flanking a counter selectable marker (*URA3*) is inserted using PCR targeting at the 3′ end of the ORF in a strain also containing a Gal1-I-SceI endonuclease cassette. Upon expression of I-SceI on galactose-containing medium, the inserted cassette is cleaved and recombination between the sfGFP repeats occurs with high efficiency.

(B) Three different cassettes, containing only sfGFP, or sfGFP and either $PHO5_T$ or $PHO5_{T:scr}$ were used to generate for each gene three different strains.

(C) RNA and protein expression analysis for the *IME4* gene using strains constructed with the method described in (A) and (B). Top left: expression data from tiling arrays of the *IME4* genomic region in haploid cells for the Watson (top) and Crick (bottom) strands are shown. Plots show normalized signal intensities from three hybridizations (YPD1, YPD2, and YPD3) using cells grown in rich medium (YPD). Transcript boundaries are depicted in red, and the darker blue color indicates a higher hybridization signal. Top right: *IME4* RNA and asRNA abundances measured by qRT-PCR using strand specific primers for the sfGFP sequence. The values were normalized to the *ACT1* gene. Bottom: western blotting and quantification of the signal of Ime4-sfGFP (Ime4). Pgk1 was used as a loading control. One representative blot is shown. Quantifications are based on three replicates, normalized using Pgk1 as a reference. Error bars indicate SD.

(D) Three categories of genes were chosen for tagging based on the data from Xu et al. (2009), as indicated. The transcript start site (TSS) area of the sense gene is shown in purple.

S3). The measured sfGFP intensities are given in Table S2. Because the original RNA expression dataset used to annotate the SUTs was obtained from a diploid strain (Xu et al., 2009), we repeated the strand-specific tiling array assay using the haploid library background strain and using the same four growth conditions. Microscopy-based protein measurements and RNA levels measured by tiling arrays exhibited the correlation expected from the literature (R^2 between 0.49 and 0.62; Figure S2D; Csárdi et al., 2015). Depending on the growth condition, between 121 and 139 genes showed sfGFP fluorescence above background (Figure 2B), even though our library was enriched for low-expressed genes (Figure S2E). This demonstrates the validity and sensitivity of our approach.

Next, we identified genes where the suppression of antisense transcription led to significant changes in protein abundance (Figures 2B and 2C; the genes are listed in Table S4). We calculated

OPEN
ACCESS
CellPress

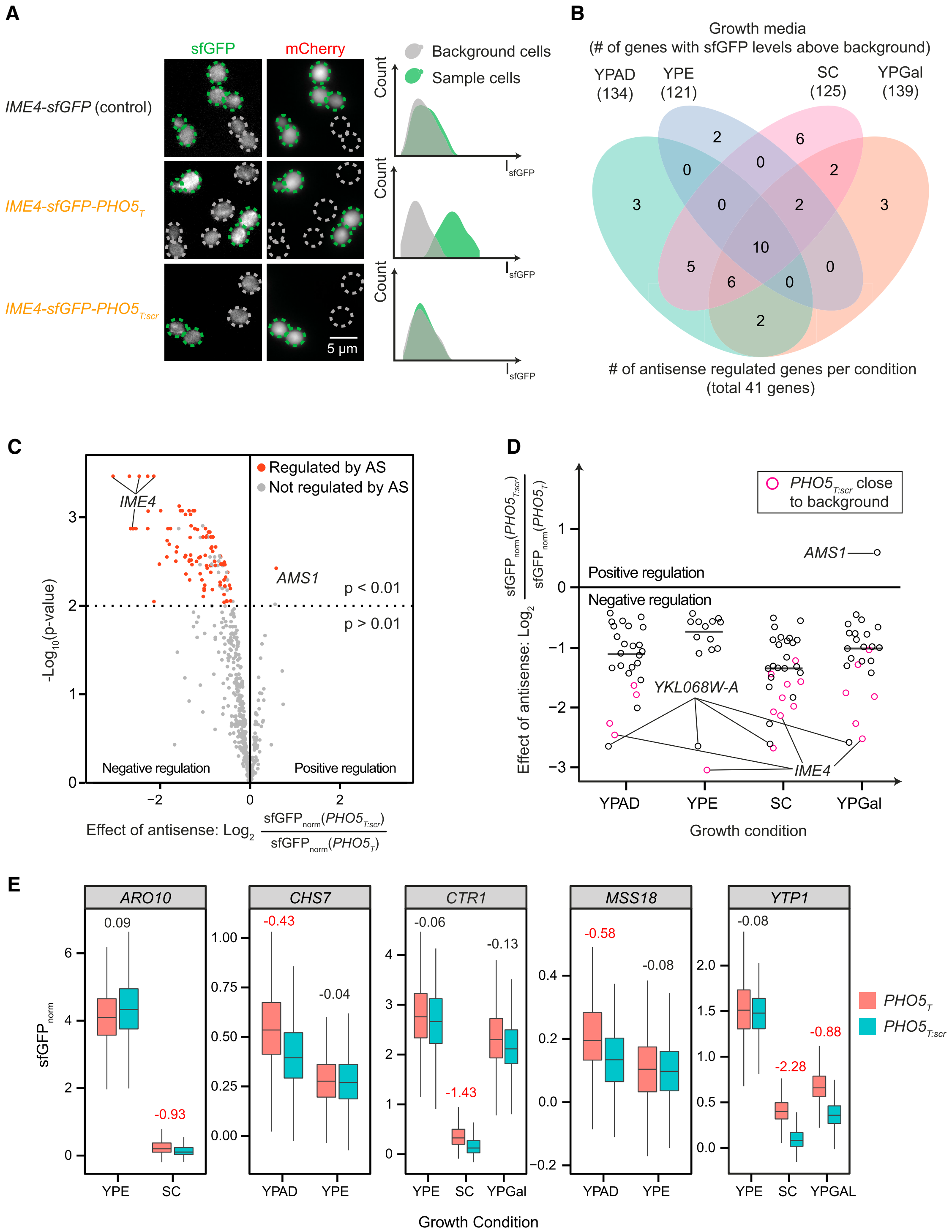

Figure 2. High-Throughput Quantitative Microscopy Identifies Antisense-Regulated Genes

(A) Cells were imaged, and whole-cell fluorescence intensities were measured using co-cultured non-fluorescent cells (identified by differential labeling with an mCherry reporter) for background subtraction and fluorescence normalization on a per-well basis (Experimental Procedures). Example cells for *IME4* in YPAD are shown. Gray, non-fluorescent cells; green, sfGFP-expressing sample cells.

(B) Venn diagram showing the number of antisense-regulated genes under the different growth conditions. Numbers in parentheses indicate the total number of genes with sfGFP levels above background (for $PHO5_T$ and/or $PHO5_{T:scr}$). In total, 41 genes were found to be antisense regulated under at least one condition.

(C) A volcano plot of p values versus log2-fold changes: ratio of sfGFP levels between $PHO5_{T:scr}$ (with antisense) and $PHO5_T$ (no antisense) strains. Each dot represents one gene in one growth condition. Red indicates genes that met our criteria for being regulated by antisense (see text).

(legend continued on next page)

the average fold change of the sfGFP levels between the $PHO5_{T:scr}$ and the $PHO5_T$ constructs. These values were well reproducible, as judged by a repetition of the experiment using a subset of the strains (R^2 = 0.81; Figure S2F). p values were obtained using a linear modeling approach (Experimental Procedures), and the significance threshold was set to $p < 0.01$. In addition, we stipulated that protein expression values of $PHO5_{T:scr}$ and sfGFP (*wt*) constructs be within a 50% expression range of each other, which was true for all but six cases, and that protein abundances between the $PHO5_T$ and $PHO5_{T:scr}$ constructs differed more than biological replicates (Experimental Procedures). Depending on the growth condition, we found between 14 out of 121 (12%) and 31 out of 125 (25%) genes with significant differences between the $PHO5_T$ and $PHO5_{T:scr}$ constructs. This corresponds to 41 genes where significant regulation by antisense was observed under at least one condition (Figures 2B and 2C).

On average, the presence of antisense led to an ~2-fold reduction in protein abundance, with effect sizes ranging from 1.35-fold (*CHS7*) to 6.3-fold (*YKL068W-A*; Figure 2D). Only one gene, *AMS1*, was positively regulated by antisense (Figures 2C and 2D). Depending on the growth condition, between 1 and 9 of the antisense-regulated genes had in the $PHO5_{T:scr}$ background sfGFP levels that were below the threshold we determined for calling a gene "expressed," suggesting a potential antisense-dependent on/off switch for these genes (Figure 2D; Table S4).

Next, we looked for genes that were regulated by antisense under only a subset of the conditions where expression was observed (Experimental Procedures). We found five such genes (Figure 2E). Interestingly, in all of those cases regulation was absent in YPE. Mostly, however, regulation between conditions did not change significantly.

To validate our findings, we used northern blotting to detect the mRNAs of a selected number of antisense-regulated genes (Figure S3A). In agreement with microscopy results, the mRNA levels were higher in the strains with the $PHO5_T$ than in the strains with the control or $PHO5_{T:scr}$ constructs. In some instances, we noted that the sense transcripts were slightly shorter for $PHO5_T$ than for $PHO5_{T:scr}$. It is currently unclear whether this is due to an antisense-specific regulation of the sense polyadenylation site (see Discussion).

These data shed light on which antisense transcripts influence sense protein levels in a native context and provide evidence that SUTs can change the protein abundance of their parent genes in at least 12%–25% of the cases. The majority of the genes was found to be negatively regulated by antisense.

The Regulatory Effect of Antisense Correlates with Antisense Levels and Anticorrelates with Sense Levels

In order to better understand the relationship of antisense-dependent effects and gene expression, we first explored whether there is a correlation between the strength of the effect of antisense regulation (microscopy data) and sense/antisense RNA levels (tiling array data). We found that the repressive effect of antisense is stronger with increasing antisense levels (Spearman's $\rho = -0.20$; Figures 3A and S4A). However, the antisense to sense ratio for a particular gene was a stronger predictor for the repressive effect ($\rho = -0.43$; Figures S4B and S4C). Finally, we found that the repressive effect of antisense declines with increasing protein expression levels ($\rho = 0.44$; Figures 3B and S4D) and that highly expressed genes ($sfGFP_{norm}$ of $PHO5_{T:scr} > 1.63$) did not exhibit regulation by antisense. These trends suggest that sense and antisense inhibit one another. However, there is also a considerable variability between genes in their susceptibility for antisense dependent regulation.

Regulation by Antisense Correlates with Transcript Start Site Overlap

Previous studies showed that antisense transcription across the TSS of the overlapping gene may cause repression of the latter mediated by a variety of chromatin-dependent mechanisms (Castelnuovo et al., 2014, 2013; van Werven et al., 2012). Consistent with this, we found that genes whose antisense transcript overlaps the TSS had a higher chance of being antisense regulated than genes whose antisense transcript terminated earlier (Figure 4A). While the size of the antisense effect was not higher in the case of TSS overlap (Figure 4B), more genes showed an ''all or nothing'' behavior (11/26 in set 1 versus 2/12 in set 2; Table S4).

TSS overlaps were classified based on tiling array data. Native elongating transcript sequencing (NET-seq; Churchman and Weissman, 2011) is a complementary method that reports on the positions of actively transcribing RNA polymerase complexes. It is thus capable of detecting transcriptional events not visible by bulk analysis methods such as tiling arrays. We made use of available NET-seq data (Churchman and Weissman, 2011) and plotted their read numbers in antisense direction relative to the position of the TSS or the stop codon of the genes in our dataset (Figure 4C). This revealed for antisense-regulated genes the presence of active antisense transcription at the sense TSS even if the respective SUTs were annotated to not overlap the TSS. Similarly, regulated genes without annotated SUTs also showed reads near the TSS. In contrast, non-regulated genes in those two groups did not show any reads near the TSS. For those genes where TSS overlap was annotated, both regulated and non-regulated genes showed reads near the TSS. This suggests that TSS overlap is not a sufficient determinant of regulation. As an alternative explanation, we noted that the peak of antisense transcription for the non-regulated genes was clearly shifted into the coding sequence at the stop codon. This could mean that some of

(D) Log2-fold changes between $PHO5_{T:scr}$ and $PHO5_T$ for each growth condition. Selected examples are labeled. Short black bars indicate the median. Colored circles indicate genes whose sfGFP intensities could not be reliably distinguished from background for the $PHO5_{T:scr}$ construct. To be able to visualize these genes in the diagram, a small offset for the $PHO5_{T:scr}$ expression value was introduced if necessary (see Experimental Procedures for details).

(E) Box plots of sfGFP intensities ($sfGFP_{norm}$, normalized to the fluorescence of the control cells without sfGFP) are shown for genes found to exhibit condition-specific regulation by antisense. Only the conditions differing in their regulatory strength are shown. Numbers indicate the log2-fold change of $PHO5_{T:scr}$ over $PHO5_T$. Red numbers indicate that the gene was found to be significantly regulated in that condition and fill colors represent the construct ($PHO5_T$ or $PHO5_{T:scr}$). Populations of both biological replicates were pooled in the boxplots for simplicity.

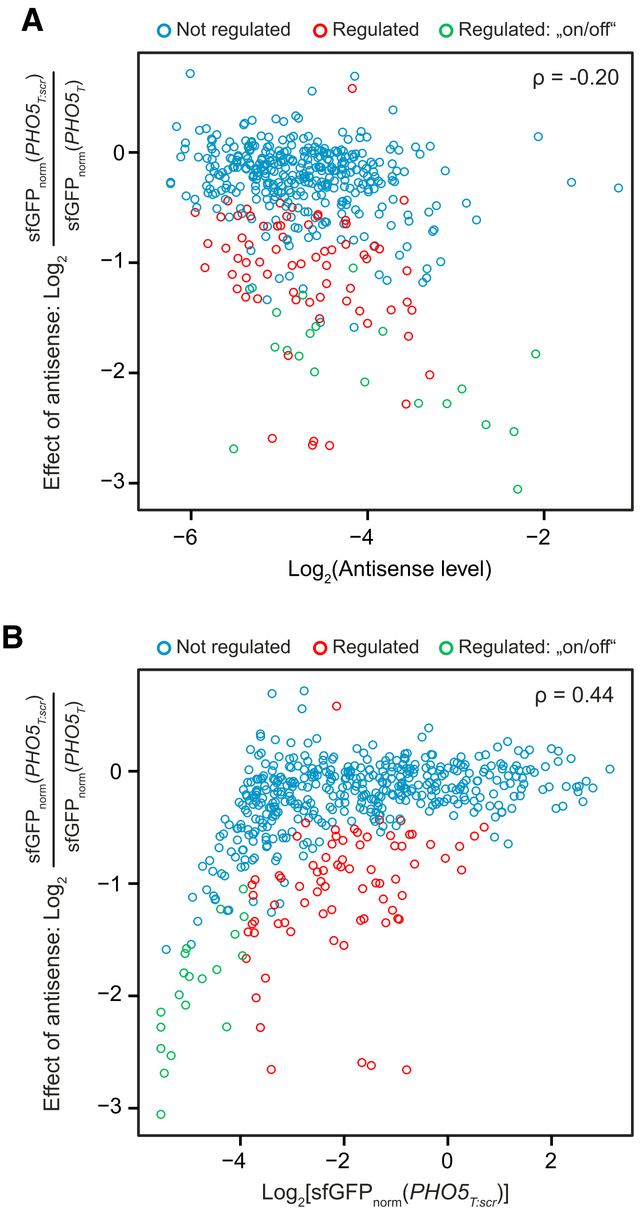

Figure 3. Correlation of Regulation by Antisense with Antisense and Protein Levels

(A) The effect of antisense on protein levels (log2-fold change of sfGFP levels between $PHO5_{T:scr}$ and $PHO5_T$) versus antisense transcript levels. Data obtained for all conditions from fluorescence microscopy and from tiling arrays are shown. These were obtained using the same strain background and the same growth conditions. Blue circles represent genes not found to be regulated by antisense. Red and green circles indicate genes with regulation by antisense with the latter being subject to an antisense-dependent on/off switch (see Figure 2 and main text). Spearman's correlation coefficient ρ is indicated in the plot.

(B) As in (A), but with sfGFP intensities of the $PHO5_{T:scr}$ construct plotted on the x axis.

the antisense transcripts in this group initiated in the coding region, which would result in a failure of termination by the $PHO5_T$ construct.

In summary, the NET-seq data analysis further strengthens the notion of a functional connection between antisense regulation and antisense transcription across the sense TSS.

Antisense-Regulated Genes Show Increased H3K4 Di- and Trimethylation

We proceeded to identify more features correlating with regulation by antisense. To ensure that potential differences are not due to differences in transcript levels, we did not consider genes without annotated antisense for this and subsequent comparisons. Of the remaining genes, antisense regulated and non-regulated genes showed similar sense and antisense levels, as measured by tiling arrays (Figures S5A and S5B). Since histone modifications have been implicated in antisense-dependent gene regulation (Camblong et al., 2007; Castelnuovo et al., 2014; Uhler et al., 2007; van Werven et al., 2012), we first tested whether specific chromatin modifications are associated with functional antisense transcripts. We made use of available datasets (Experimental Procedures) and compared histone traces of antisense-regulated and non-regulated genes relative to their TSSs and their stop codons. This revealed increased H3K4 di- and trimethylation (H3K4me2/3) at the 3′ end of the regulated genes (Figures 5A and 5B). H3R2me2 is known to counteract H3K4me3 (Kirmizis et al., 2007). Consistent with this, H3R2me2 was decreased in this region (Figure S5C). We could not detect significant changes for other histone modifications (Table S5). This implies a functional relevance of H3K4 methylation in gene regulation by antisense transcripts.

Lack of Obvious Sequence Features Associated with Regulation by Antisense

Next, we searched for sequence motifs associated with genes in either the antisense regulated or the non-regulated group. Extensive searches using databases of annotated DNA-protein binding sites, a catalog of RNA-protein interaction sites, and de novo motif identification tools did not reveal any significant results (Bailey et al., 2009; Hogan et al., 2008; Shannon, 2015; Venters et al., 2011; see Experimental Procedures for further details). Of special interest in this respect are motifs specific for transcription attenuation by the NNS complex known to limit non-coding transcription of CUTs in yeast (Arigo et al., 2006; Schulz et al., 2013). The involvement of the NNS complex in early termination and the correlation of regulation by antisense and TSS overlap suggested that non-regulated genes might be enriched in Nrd1-Nab3 binding sites. To test this, we used previously published data on Nrd1 binding sites (Hogan et al., 2008). The fraction of genes that displayed at least one Nrd1 binding site in antisense direction was increased in non-regulated genes versus regulated ones (25 out of 92 versus 5 out of 39), but this increase was not significant (Figure 5C) and may in part be due to the increased length of non-regulated genes versus regulated ones (Figure 5D). A similar comparison using strand-specific photoactivatable ribonucleoside-enhanced crosslinking and immunoprecipitation (PAR-CLIP) data (Creamer et al., 2011) did not reveal any significant differences either (data not shown).

Together, this suggests that antisense transcripts do not contain sequence information sufficient to predict their regulatory potential.

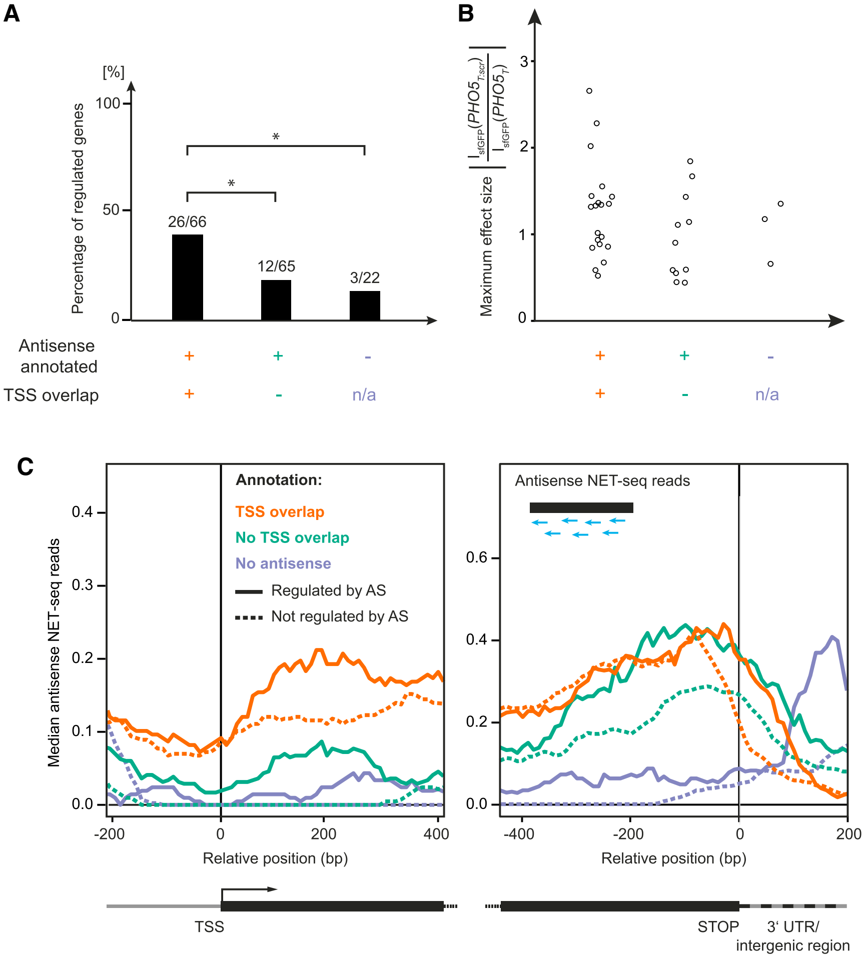

Figure 4. TSS Overlap Correlates with Regulation by Antisense Transcription

(A) Genes that were tested for antisense regulation in at least one condition were divided into three groups that differ in the degree of antisense overlap with the sense (see main text). Next, the number of genes that were regulated by antisense in at least one condition was determined. The frequency of genes regulated in at least one condition is significantly higher when the antisense is annotated to overlap the TSS than in the group without annotated TSS overlap or the group where no antisense has been annotated (Fisher's exact test, $p < 0.05$).

(B) For every gene found to be regulated in at least one condition the maximum significant log2-fold-change between $PHO5_{T:scr}$ and $PHO5_T$ was determined across growth conditions. This does not include genes with an "all or nothing" behavior (see main text).

(C) Genes were aligned at their TSS (left) or stop codons (right). The aligned NET-seq traces are shown using group-wise smoothed medians (Experimental Procedures). Colors indicate the extent of SUT overlap with the sense as annotated in Xu et al. (2009). Solid lines indicate antisense-regulated genes, whereas dashed lines indicate non-regulated genes.

Antisense-Regulated Genes Show Reduced Protein Expression Noise

Previous studies discussed a correlation between the presence of an overlapping antisense transcript and the degree of cell-to-cell variability ("noise") in protein abundance (Pelechano and Steinmetz, 2013; Xu et al., 2011). We followed the flow cytometry based noise measurement strategy of Newman et al. (Newman et al., 2006; see Experimental Procedures for details) to investigate differences in noise levels between $PHO5_T$ and $PHO5_{T:scr}$ strains. Briefly, a special gating procedure is applied to select for a homogeneous population of unbudded G1 cells (Figure S6A). Consequently, sources of extrinsic noise are minimized and the resulting coefficient of variation (CV % = (SD/mean) × 100) values are composed of intrinsic noise and extrinsic noise specific to the pathway that regulates that gene's expression (Newman et al., 2006; Raser and O'Shea, 2005). The limited sensitivity of flow cytometry meant that we could only measure noise levels for 66 genes, including 14 antisense-regulated ones (see Figure S6B for a comparison with microscopy). As previously reported, we observed an inverse proportional relationship between CV^2 and protein abundance (Figure 6A; Newman et al., 2006; Paulsson, 2004). For the antisense-regulated genes, protein abundances of the $PHO5_{T:scr}$ strains are on average smaller than for the $PHO5_T$ strains (Figure S6B). This also applies for the genes considered to be not regulated by antisense, albeit in a non-significant manner. Because of this slight deviation from zero, a direct comparison of noise between the two categories will be strongly affected by changes in protein abundances (Figure S6C). To obtain noise levels independent of this confounding influence, we adapted the analysis procedure of Newman et al., 2006 (Newman et al., 2006). We calculated the residuals of each CV value (CV_{res}) to a robust regression model of the CV values (Figure 6A). By calculating the difference in the CV_{res} values of $PHO5_{T:scr} - PHO5_T$, one can compare the noise levels of the two constructs for a given gene and for different categories in general. Interestingly, the distributions of the gene-wise differences in CV_{res} values were significantly smaller for antisense regulated genes than for non-regulated ones (Wilcoxon's rank sum test, $p < 0.05$; Figure 6B) and were mainly negative. We conclude that, provided that a gene is antisense regulated, noise levels are reduced in the presence of antisense transcription.

DISCUSSION

Reports that provide experimental evidence for the implications of antisense regulation on the expressed protein amounts of the overlapping sense gene are scarce, with most studies focusing on RNA levels or on individual genes. Here, we investigated the impact of SUT antisense transcripts on expressed protein amounts for a larger group of genes. We employed yeast

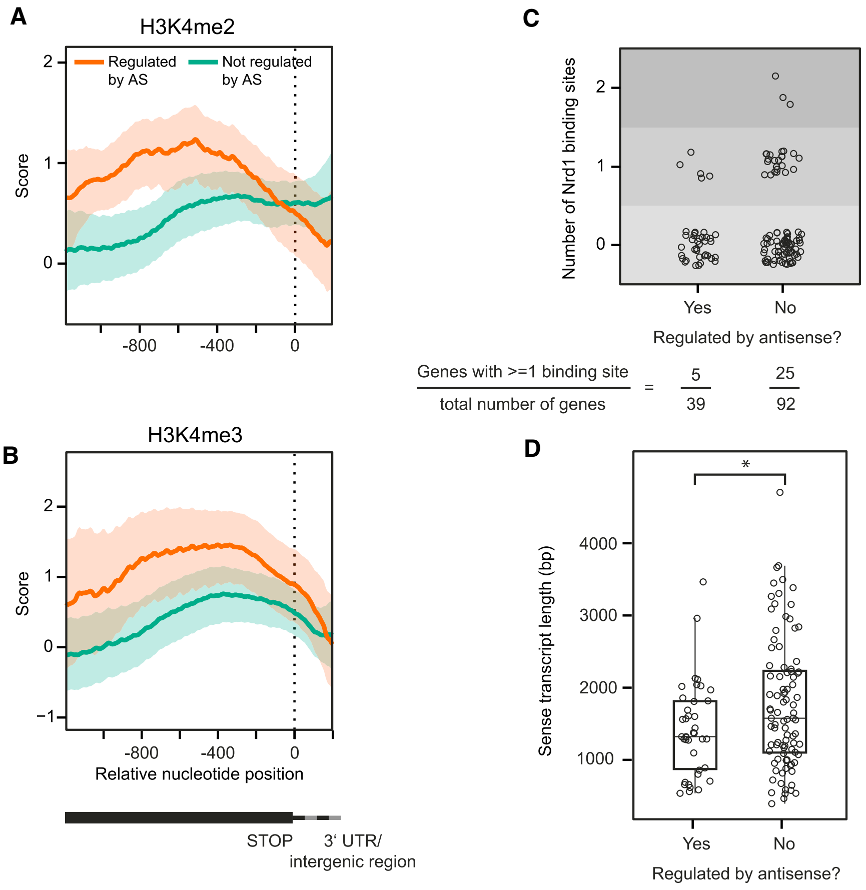

Figure 5. Features Correlating with Regulation by Antisense

(A and B) H3K4me2 and H3K4me3 densities in antisense-regulated (red) versus non-regulated (green) genes. All genes were aligned at their stop codons. Lines and ribbons indicate bootstrapping-based mean and 95% confidence interval estimates, respectively.

(C) The number of Nrd1 binding sites as determined previously (Hogan et al., 2008) was determined for all transcripts in our library and the number of binding sites per gene is shown, grouped by whether the gene was found to be regulated by antisense in at least one condition or not.

(D) Transcript lengths of genes grouped by whether they were regulated by antisense in at least one condition or not. The groups are significantly different (Wilcoxon's rank sum test, $p < 0.05$).

high-throughput strain construction and a strategy to specifically terminate antisense transcription and used this to investigate the function of >150 SUTs in regulating protein levels under four growth conditions. We found that SUTs led to a significant reduction of protein abundance for approximately one quarter of the genes. Therefore, the mere presence of an antisense SUT for a given ORF is no indication for a functional role of this ncRNA. Our results provide experimental confirmation for the previous observations from transcriptomics studies that antisense transcription seems to generally repress sense expression. Our finding that 41 out of 152 genes with detectable GFP signals exhibit antisense-dependent gene regulation under at least one condition is consistent with the idea that only a fraction of genes are sensitive to antisense transcription (Alcid and Tsukiyama, 2014; Castelnuovo et al., 2014; Schulz et al., 2013; Xu et al., 2009). We observed that the regulatory effect of antisense SUTs tended to be weak, leading on average to a 50% reduction in protein levels. Our data show that both antisense levels and antisense/sense ratios correlated with the repressive effect of antisense. At high sense levels, repressive effects were markedly reduced. We also found that the regulatory potential of antisense transcripts was increased in the case of TSS overlap and was paralleled by a reduction in protein expression noise.

The strategy to employ a DNA element (*PHO5*$_T$) to abrogate SUTs in a strand-specific manner bears the risk that the introduced element introduces additional disturbances, as is the case for any gene- and protein-tagging strategy. For example, a certain number of antisense transcripts may be initiated within the ORF of the gene, in which case the *PHO5*$_T$ strategy does not work. This might be the case for a number of non-regulated genes with TSS-overlapping SUTs as indicated by a pronounced shift of the NET-seq read maximum toward the inside of the coding region in this group (Figure 4C). Inhibition of an antisense transcript could also lead to the derepression of a cryptic downstream antisense transcript, thus reconstituting antisense repression. Moreover, the approach we used may occasionally introduce artifacts. For example, the actual strength and unidirectionality of our terminator may depend on the genomic context (Guo and Sherman, 1996). Northern blots showed that in some cases, the sense transcripts with the *PHO5*$_T$ were shorter than expected (Figure S3A). This could be due to premature termination or selection of an alternative poly(A) site as a function of the *PHO5*$_T$ element. Equally well, however, this could be caused as a function of the abrogated antisense RNA. Nevertheless, we are confident that the *PHO5*$_T$ element functions as expected in a majority of the cases. First, there was a neutral effect of both the *PHO5*$_T$ and the *PHO5*$_{T:scr}$ constructs on *IME4-sfGFP* RNA levels in a diploid background, where the *RME2* antisense transcript is efficiently repressed. Second, we would expect that general premature termination of transcripts results in decreased stability. The results for *IME4*, the absence of mRNA degradation products in our northern blots and the fact that protein levels for the *PHO5*$_T$ strains were usually increased indicate that this is not a general concern.

Alternative approaches to study antisense transcription employ mutants in genes that globally affect the stability and transcription of non-coding RNAs. However, such mutations are likely to influence a cell's RNA homeostasis globally, and hence the predictive value for the assignment of regulatory

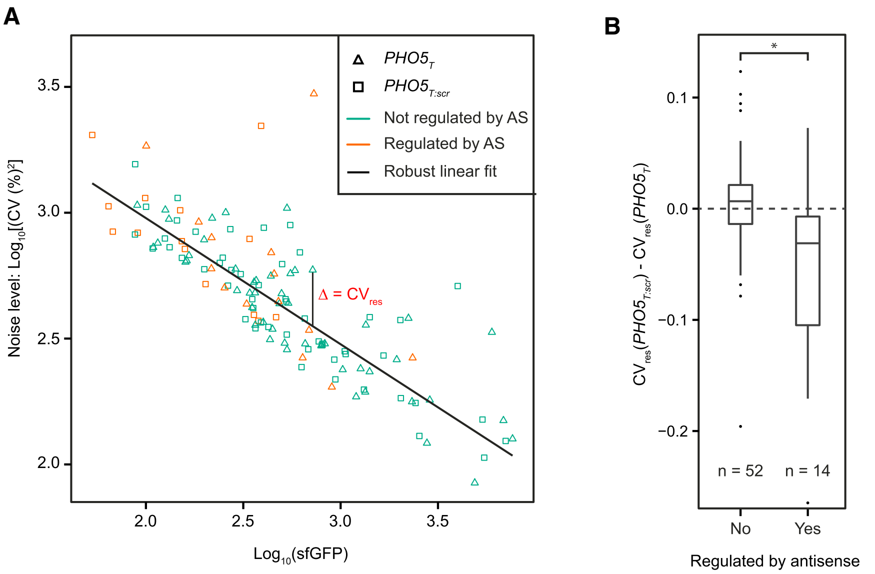

Figure 6. Impact of Antisense Suppression on Gene Expression Noise

(A) Log_{10}[CV (%)^2] versus log_{10}(sfGFP) of 66 genes permit robust linear fitting (solid black line) and the calculation of noise levels corrected for differences in abundance (CV_{res}; one example is indicated). Different fits are possible, but the result (shown in B) is the same in all cases (see also Figure S6D and Experimental Procedures). *PHO5*$_T$ and *PHO5*$_{T:scr}$ strains are indicated by triangles and squares, respectively. Genes regulated by antisense are shown in orange, whereas non-regulated genes are shown in green.

(B) Distribution of the gene-wise differences of CV_{res} values of *PHO5*$_{T:scr}$ minus *PHO5*$_T$ constructs from (A), grouped depending on whether the genes were found to be antisense regulated under the growth condition used for FACS (SC) or not. The difference between the groups is significant (Wilcoxon's rank sum test, $p < 0.05$).

functions of antisense transcripts is limited. Interestingly, the clustered regularly interspaced short palindromic repeats (CRISPR)/Cas9 system has been recently used in yeast to suppress a ncRNA in a strand-specific and position-dependent manner (Lenstra et al., 2015). This offers the advantage of not manipulating the endogenous locus. However, it requires gene-specific optimization in order to identify guide RNAs that are effective in quenching the antisense transcript (Lenstra et al., 2015), which makes it difficult to use this strategy in systemic studies.

Antisense regulation might affect gene noise with or without affecting the average expression level of a gene. Interestingly, we found that regulation of protein amounts by antisense results in a concomitant reduction in protein expression noise. Our fluorescence-activated cell sorting (FACS) measurement could only assess genes that are well expressed. For this subset of antisense-regulated genes, our result is in contrast to the view that antisense transcription increases the noise of genes in the "on" state (Pelechano and Steinmetz, 2013; Xu et al., 2011). It will be interesting to study whether the effect we observed results from antisense-regulated genes being intrinsically less noisy or because the pathways involved in antisense-dependent repression act as extrinsic low-level noise sources.

The fact that 13 of 41 genes showed full repression of protein expression under at least one growth condition (within the limits of sfGFP detection; Figure 2D; Table S4) supports the idea that antisense is frequently involved in the switching between "on" and "off" states and serves to suppress the leakiness of gene expression (Castelnuovo et al., 2013; Hongay et al., 2006; Lenstra et al., 2015; Xu et al., 2011). However, whether these potential on/off switches are associated with a physiological function remains to be determined for each individual gene.

What distinguishes antisense-regulated genes from non-regulated ones? First, our data indicate that increasing antisense levels led to a stronger regulation by antisense while higher sense levels reduced such an effect. This favors the idea that sense and antisense competitively inhibit each other as studied in detail in a previous report using an artificial gene construct in yeast (Buetti-Dinh et al., 2009). However, in contrast to that study, we could not observe a similar decrease in the repressive effect for weakly expressed genes.

Next, we found that antisense transcription overlapping the TSS is a strong predictor for the functionality of antisense transcription. NET-seq data revealed reads around the TSS even for those genes where tiling array experiments did not detect transcripts in that region. This suggests that the act of antisense transcription rather than the amount of the antisense transcript is of functional importance. This would also imply that the distinction between different types of ncRNAs such as CUTs/XUTs/NUTs/SUTs, while interesting in terms of RNA metabolism, may be less important from a functional point of view. For example, Castelnuovo and colleagues presented evidence that led them to hypothesize that the frequency of CUTs escaping early termination and extending into the sense TSS is inversely correlated with sense levels (Castelnuovo et al., 2014, 2013). We propose that similar mechanisms are at work for SUTs. In this respect, it is unclear which role the NNS complex plays in regulating protein abundance by antisense SUTs. While we found a slight increase in the number of Nrd1 binding sites in the non-regulated gene set, this increase was not significant. At the same time, early termination events are a mechanism that could help to explain how antisense SUTs are prevented from reaching the sense TSS. The fact that non-regulated genes are longer than antisense regulated ones could further decrease the chance of TSS overlap. We speculate that the impact of antisense may be modulated by a combination of strand-specific termination signals and a relative shift of TSS positions, for example by changing the lengths or initiation sites of transcription units. We also did not find any other motifs or protein binding sites associated with antisense-dependent gene regulation. Considering the importance of where in the gene antisense transcription takes place, it is possible that narrowing motif searches

down to certain positions in the gene will reveal sequence motifs missed in our analysis.

When comparing histone modification profiles across the genes of interest we found a significant increase in H3K4me2/3 distribution at the end of antisense-regulated genes. H3K4me3 is a modification associated with actively transcribed genes (Pokholok et al., 2005). Thus, one explanation might be that transcription of the antisense RNA in the regulated genes is more active in certain regions and thus redistributes this mark into the gene body, as was proposed in a recent study (Murray et al., 2015). H3K4me3 has also been linked to *SET1*-dependent antisense transcription (Castelnuovo et al., 2014; Margaritis et al., 2012). Our data provide evidence for a widespread functional role of this modification in gene regulation by SUTs. No other histone marks were found to be associated with antisense dependent gene regulation, suggesting that they are not involved in the regulation of protein amounts.

Even genes whose antisense SUTs overlapped the TSS were not always regulated by antisense transcription. This indicates that TSS overlap is either not sufficient to impose antisense-dependent regulation or that certain factors blocked such a repression under the conditions tested. Other possibilities include that the regulated genes were more responsive to certain chromatin modifications under the conditions tested, as exemplified by the genes subject to condition-specific regulation.

In summary, we conclude that the majority of antisense transcripts are unlikely to be effector molecules whose synthesis and presence is involved in regulating the abundance of the sense genes. A smaller fraction of antisense transcripts exhibit weak suppressive and denoising functions that may in some cases lead to a complete shutdown of the sense gene. Given that cases of strong and functional antisense-dependent gene regulation have been observed in yeast, this argues that single antisense transcripts may acquire new roles to regulate the sense gene by making use of a variety of different mechanisms that differ from gene to gene. This leaves room for the evolution of gene-specific mechanisms by which antisense transcription may acquire new physiologically relevant regulatory functions.

EXPERIMENTAL PROCEDURES

Yeast Strains and Culturing Conditions

Yeast cells were grown according to standard methods (Sherman, 2002). Cultures were grown to logarithmic phase (optical density 600 [OD_{600}] between 0.5 and 1.0) unless otherwise stated. See Supplemental Experimental Procedures for a list of strains and growth media.

Antisense Library Construction

Details about plasmids and strains are listed in Supplemental Experimental Procedures. Briefly, gene-specific oligos containing S2/S3 annealing sites (Knop et al., 1999) for the template cassettes (pMaM201 with *sfGFP-PHO5*$_T$, pMaM203 with *sfGFP-PHO5*$_{T:scr}$, and pMaM175 with *sfGFP*) were used to generate PCR products using a high-fidelity polymerase. Strain YMaM330 was used for transformation, and for each transformation, six clones were picked, singled out, and validated using colony PCR.

RNA Extractions and Northern Blots

For all methods, total RNA was extracted using a hot phenol protocol (Collart and Oliviero, 2001). Remaining DNA was removed using the TURBO DNA-free Kit (Life Technologies). Northern blotting is explained in Supplemental Experimental Procedures.

Tiling Arrays

The background strain of the antisense library (YMaM330) was grown to mid-log phase in one of the four growth media (YPAD, YPGal, YPE, or SC), and total RNA was extracted and hybridized to tiling arrays as described previously (Xu et al., 2009). The dataset for the whole genome is available in a searchable web database (http://steinmetzlab.embl.de//cgi-bin/viewKnopLabArray.pl?showSamples=KnopHaploidTest2&type=heatmap).

Fluorescence Microscopy

Details about imaging and image processing can be found in Supplemental Experimental Procedures. Briefly, sample cells were mixed 1:1 with sfGFP-negative control cells for background subtraction and normalization on a well-by-well basis. Exponentially growing cells were fixed and seeded on 384-well microscopy plates. Imaging was performed on a Nikon Ti-E screening wide-field epifluorescence microscope using different exposure times for sfGFP and including controls to correct for shading artifacts. Image post-processing, quantification, and quality control were performed using custom scripts in ImageJ, MATLAB (MathWorks), and R (R Core Team, 2015). The whole single-cell dataset can be downloaded from the University of Heidelberg heiDATA Dataverse Network (http://dx.doi.org/10.11588/data/10073). Scripts and raw imaging data are available upon request.

Bioinformatic and Statistical Analyses

Data analyses were mainly performed using the open source software *R* (R Core Team, 2015), making extensive use of the Bioconductor framework (Huber et al., 2015) and numerous publicly available datasets. Please see Supplemental Experimental Procedures for further details. Scripts are available upon request.

Flow Cytometry

Cells were grown to mid-log phase in SC medium in 96-well plates and analyzed using a flow cytometer equipped with a high throughput stage (BD FACSCanto RUO HTS). 100,000 events were recorded per well. Gating and calculations to obtain noise estimates were done in R with the help of the flowCore package (Ellis et al., 2015), essentially using the method of Newman et al. (2006). See Supplemental Experimental Procedures for further details. Scripts and data files can be downloaded from the University of Heidelberg heiDATA Dataverse Network (http://dx.doi.org/10.11588/data/10073).

Western Blots

Proteins were extracted from mid log phase cell cultures grown in the respective medium using the trichloroacetic acid (TCA) method (Knop et al., 1999). Proteins were then separated by SDS-PAGE as described (Laemmli, 1970) and transferred to nitrocellulose membranes using a semi-wet blotter (XCell II Blot Module; Invitrogen). Membranes were incubated overnight with primary anti-GFP antibodies (Abcam) or anti-PGK1 antibodies (Molecular Probes). Secondary antibodies were labeled with $Alexa_{680}$ (Invitrogen) or $IRDye_{800}$ (Rockland Immunochemicals). Detection and quantification was performed with an Odyssey Infrared Imaging System (Li-Cor Biosciences).

qRT-PCRs

DNase-treated RNA from exponentially growing yeast cells was used as an input for reverse transcription using 2 pmol gene-specific primer and 1 μg RNA using SuperScript III reverse transcriptase (Invitrogen) according to the manufacturer's instructions but supplemented with 20 μg/ml actinomycin D to ensure strand specificity of the reverse transcription (Perocchi et al., 2007). For qPCR, cDNA samples and -RT controls were diluted to 1 ml, and 2.5 μl were amplified using the LightCycler 480 SYBR Green I Master Mix (Roche). Actin mRNA was used as the reference gene.

ACCESSION NUMBERS

The accession number for the tiling array data reported in this paper is ArrayExpress: E-MTAB-4731.

SUPPLEMENTAL INFORMATION

Supplemental Information includes Supplemental Experimental Procedures, six figures, and five tables and can be found with this article online at http://dx.doi.org/10.1016/j.celrep.2016.05.043.

AUTHOR CONTRIBUTIONS

M.K. conceived the project with input from A.K. and L.M.S.; A.K., M.M., and D.B. constructed the library; F.H. and D.B. designed and analyzed the experiments with input from I.G.; D.B. and F.H. performed the microscopy with technical assistance from P.T.; I.G. performed and analyzed the tiling array experiments; F.H. performed bioinformatics and statistical analyses with input from I.G.; all authors commented on the manuscript; and F.H., D.B., and M.K. wrote the manuscript.

ACKNOWLEDGMENTS

We thank Julien Gagneur, Zhenyu Xu, and Wu Wei for their help selecting genes for antisense library construction; Gaël Yvert, Florent Chuffart, Bernd Fischer, and the anonymous reviewers for helpful advice or suggestions; and Allan Jones and Martin Štefl for comments on the manuscript. This work was supported by the Deutsche Forschungsgemeinschaft (DFG-KN498/8-1 to M.K. and DFG-STE1422/3-1 to L.M.S.), the Sonderforschungsbereich 1036 (SFB1036, to F.H. and A.K.), a PhD fellowship from the Boehringer Ingelheim Fonds (to D.B.), and a PhD fellowship from the HBIGS graduate school (to F.H.).

Received: February 11, 2016
Revised: April 8, 2016
Accepted: May 10, 2016
Published: June 9, 2016

REFERENCES

Alcid, E.A., and Tsukiyama, T. (2014). ATP-dependent chromatin remodeling shapes the long noncoding RNA landscape. Genes Dev. *28*, 2348–2360.

Arigo, J.T., Eyler, D.E., Carroll, K.L., and Corden, J.L. (2006). Termination of cryptic unstable transcripts is directed by yeast RNA-binding proteins Nrd1 and Nab3. Mol. Cell *23*, 841–851.

Bailey, T.L., Boden, M., Buske, F.A., Frith, M., Grant, C.E., Clementi, L., Ren, J., Li, W.W., and Noble, W.S. (2009). MEME SUITE: tools for motif discovery and searching. Nucleic Acids Res. *37*, W202–W208.

Bertone, P., Stolc, V., Royce, T.E., Rozowsky, J.S., Urban, A.E., Zhu, X., Rinn, J.L., Tongprasit, W., Samanta, M., Weissman, S., et al. (2004). Global identification of human transcribed sequences with genome tiling arrays. Science *306*, 2242–2246.

Buetti-Dinh, A., Ungricht, R., Kelemen, J.Z., Shetty, C., Ratna, P., and Becskei, A. (2009). Control and signal processing by transcriptional interference. Mol. Syst. Biol. *5*, 300.

Camblong, J., Iglesias, N., Fickentscher, C., Dieppois, G., and Stutz, F. (2007). Antisense RNA stabilization induces transcriptional gene silencing via histone deacetylation in S. cerevisiae. Cell *131*, 706–717.

Camblong, J., Beyrouthy, N., Guffanti, E., Schlaepfer, G., Steinmetz, L.M., and Stutz, F. (2009). Trans-acting antisense RNAs mediate transcriptional gene cosuppression in S. cerevisiae. Genes Dev. *23*, 1534–1545.

Castelnuovo, M., Rahman, S., Guffanti, E., Infantino, V., Stutz, F., and Zenklusen, D. (2013). Bimodal expression of PHO84 is modulated by early termination of antisense transcription. Nat. Struct. Mol. Biol. *20*, 851–858.

Castelnuovo, M., Zaugg, J.B., Guffanti, E., Maffioletti, A., Camblong, J., Xu, Z., Clauder-Münster, S., Steinmetz, L.M., Luscombe, N.M., and Stutz, F. (2014). Role of histone modifications and early termination in pervasive transcription and antisense-mediated gene silencing in yeast. Nucleic Acids Res. *42*, 4348–4362.

Churchman, L.S., and Weissman, J.S. (2011). Nascent transcript sequencing visualizes transcription at nucleotide resolution. Nature *469*, 368–373.

Collart, M.A., and Oliviero, S. (2001). Preparation of yeast RNA. In Current Protocols in Molecular Biology (John Wiley & Sons).

Creamer, T.J., Darby, M.M., Jamonnak, N., Schaughency, P., Hao, H., Wheelan, S.J., and Corden, J.L. (2011). Transcriptome-wide binding sites for components of the Saccharomyces cerevisiae non-poly(A) termination pathway: Nrd1, Nab3, and Sen1. PLoS Genet. *7*, e1002329.

Csárdi, G., Franks, A., Choi, D.S., Airoldi, E.M., and Drummond, D.A. (2015). Accounting for experimental noise reveals that mRNA levels, amplified by post-transcriptional processes, largely determine steady-state protein levels in yeast. PLoS Genet. *11*, e1005206.

David, L., Huber, W., Granovskaia, M., Toedling, J., Palm, C.J., Bofkin, L., Jones, T., Davis, R.W., and Steinmetz, L.M. (2006). A high-resolution map of transcription in the yeast genome. Proc. Natl. Acad. Sci. USA *103*, 5320–5325.

Davis, C.A., and Ares, M., Jr. (2006). Accumulation of unstable promoter-associated transcripts upon loss of the nuclear exosome subunit Rrp6p in Saccharomyces cerevisiae. Proc. Natl. Acad. Sci. USA *103*, 3262–3267.

Drinnenberg, I.A., Weinberg, D.E., Xie, K.T., Mower, J.P., Wolfe, K.H., Fink, G.R., and Bartel, D.P. (2009). RNAi in budding yeast. Science *326*, 544–550.

Drinnenberg, I.A., Fink, G.R., and Bartel, D.P. (2011). Compatibility with killer explains the rise of RNAi-deficient fungi. Science *333*, 1592.

Ellis, B., Haaland, P., Hahne, F., Le Meur, N., Gopalakrishnan, N., Spidlen, J., Jiang, M., 2015. flowCore: basic structures for flow cytometry data. R package version 1.38.1.

Gavin, A.-C., Aloy, P., Grandi, P., Krause, R., Boesche, M., Marzioch, M., Rau, C., Jensen, L.J., Bastuck, S., Dümpelfeld, B., et al. (2006). Proteome survey reveals modularity of the yeast cell machinery. Nature *440*, 631–636.

Gelfand, B., Mead, J., Bruning, A., Apostolopoulos, N., Tadigotla, V., Nagaraj, V., Sengupta, A.M., and Vershon, A.K. (2011). Regulated antisense transcription controls expression of cell-type-specific genes in yeast. Mol. Cell. Biol. *31*, 1701–1709.

Ghaemmaghami, S., Huh, W.-K., Bower, K., Howson, R.W., Belle, A., Dephoure, N., O'Shea, E.K., and Weissman, J.S. (2003). Global analysis of protein expression in yeast. Nature *425*, 737–741.

Guo, Z., and Sherman, F. (1996). Signals sufficient for 3′-end formation of yeast mRNA. Mol. Cell. Biol. *16*, 2772–2776.

Hogan, D.J., Riordan, D.P., Gerber, A.P., Herschlag, D., and Brown, P.O. (2008). Diverse RNA-binding proteins interact with functionally related sets of RNAs, suggesting an extensive regulatory system. PLoS Biol. *6*, e255.

Hongay, C.F., Grisafi, P.L., Galitski, T., and Fink, G.R. (2006). Antisense transcription controls cell fate in Saccharomyces cerevisiae. Cell *127*, 735–745.

Huber, W., Carey, V.J., Gentleman, R., Anders, S., Carlson, M., Carvalho, B.S., Bravo, H.C., Davis, S., Gatto, L., Girke, T., et al. (2015). Orchestrating high-throughput genomic analysis with Bioconductor. Nat. Methods *12*, 115–121.

Huh, W.-K., Falvo, J.V., Gerke, L.C., Carroll, A.S., Howson, R.W., Weissman, J.S., and O'Shea, E.K. (2003). Global analysis of protein localization in budding yeast. Nature *425*, 686–691.

Irniger, S., Egli, C.M., and Braus, G.H. (1991). Different classes of polyadenylation sites in the yeast Saccharomyces cerevisiae. Mol. Cell. Biol. *11*, 3060–3069.

Khmelinskii, A., Meurer, M., Duishoev, N., Delhomme, N., and Knop, M. (2011). Seamless gene tagging by endonuclease-driven homologous recombination. PLoS ONE *6*, e23794.

Khmelinskii, A., Blaszczak, E., Pantazopoulou, M., Fischer, B., Omnus, D.J., Le Dez, G., Brossard, A., Gunnarsson, A., Barry, J.D., Meurer, M., et al. (2014). Protein quality control at the inner nuclear membrane. Nature *516*, 410–413.

Kirmizis, A., Santos-Rosa, H., Penkett, C.J., Singer, M.A., Vermeulen, M., Mann, M., Bähler, J., Green, R.D., and Kouzarides, T. (2007). Arginine methylation at histone H3R2 controls deposition of H3K4 trimethylation. Nature *449*, 928–932.

Knop, M., Siegers, K., Pereira, G., Zachariae, W., Winsor, B., Nasmyth, K., and Schiebel, E. (1999). Epitope tagging of yeast genes using a PCR-based strategy: more tags and improved practical routines. Yeast *15* (*10B*), 963–972.

Laemmli, U.K. (1970). Cleavage of structural proteins during the assembly of the head of bacteriophage T4. Nature *227*, 680–685.

Lenstra, T.L., Coulon, A., Chow, C.C., and Larson, D.R. (2015). Single-molecule imaging reveals a switch between spurious and functional ncRNA transcription. Mol. Cell *60*, 597–610.

Margaritis, T., Oreal, V., Brabers, N., Maestroni, L., Vitaliano-Prunier, A., Benschop, J.J., van Hooff, S., van Leenen, D., Dargemont, C., Géli, V., and Holstege, F.C.P. (2012). Two distinct repressive mechanisms for histone 3 lysine 4 methylation through promoting 3′-end antisense transcription. PLoS Genet. *8*, e1002952.

Marquardt, S., Escalante-Chong, R., Pho, N., Wang, J., Churchman, L.S., Springer, M., and Buratowski, S. (2014). A chromatin-based mechanism for limiting divergent noncoding transcription. Cell *157*, 1712–1723.

Murray, S.C., Haenni, S., Howe, F.S., Fischl, H., Chocian, K., Nair, A., and Mellor, J. (2015). Sense and antisense transcription are associated with distinct chromatin architectures across genes. Nucleic Acids Res. *43*, 7823–7837.

Nadal-Ribelles, M., Solé, C., Xu, Z., Steinmetz, L.M., de Nadal, E., and Posas, F. (2014). Control of Cdc28 CDK1 by a stress-induced lncRNA. Mol. Cell *53*, 549–561.

Nagalakshmi, U., Wang, Z., Waern, K., Shou, C., Raha, D., Gerstein, M., and Snyder, M. (2008). The transcriptional landscape of the yeast genome defined by RNA sequencing. Science *320*, 1344–1349.

Neil, H., Malabat, C., d'Aubenton-Carafa, Y., Xu, Z., Steinmetz, L.M., and Jacquier, A. (2009). Widespread bidirectional promoters are the major source of cryptic transcripts in yeast. Nature *457*, 1038–1042.

Newman, J.R.S., Ghaemmaghami, S., Ihmels, J., Breslow, D.K., Noble, M., DeRisi, J.L., and Weissman, J.S. (2006). Single-cell proteomic analysis of S. cerevisiae reveals the architecture of biological noise. Nature *441*, 840–846.

Nguyen, T., Fischl, H., Howe, F.S., Woloszczuk, R., Serra Barros, A., Xu, Z., Brown, D., Murray, S.C., Haenni, S., Halstead, J.M., et al. (2014). Transcription mediated insulation and interference direct gene cluster expression switches. eLife *3*, e03635.

Paulsson, J. (2004). Summing up the noise in gene networks. Nature *427*, 415–418.

Pédelacq, J.-D., Cabantous, S., Tran, T., Terwilliger, T.C., and Waldo, G.S. (2006). Engineering and characterization of a superfolder green fluorescent protein. Nat. Biotechnol. *24*, 79–88.

Pelechano, V., and Steinmetz, L.M. (2013). Gene regulation by antisense transcription. Nat. Rev. Genet. *14*, 880–893.

Perocchi, F., Xu, Z., Clauder-Münster, S., and Steinmetz, L.M. (2007). Antisense artifacts in transcriptome microarray experiments are resolved by actinomycin D. Nucleic Acids Res. *35*, e128.

Pokholok, D.K., Harbison, C.T., Levine, S., Cole, M., Hannett, N.M., Lee, T.I., Bell, G.W., Walker, K., Rolfe, P.A., Herbolsheimer, E., et al. (2005). Genome-wide map of nucleosome acetylation and methylation in yeast. Cell *122*, 517–527.

R Core Team (2015). R: A Language and Environment for Statistical Computing (R Foundation for Statistical Computing).

Raser, J.M., and O'Shea, E.K. (2005). Noise in gene expression: origins, consequences, and control. Science *309*, 2010–2013.

Schulz, D., Schwalb, B., Kiesel, A., Baejen, C., Torkler, P., Gagneur, J., Soeding, J., and Cramer, P. (2013). Transcriptome surveillance by selective termination of noncoding RNA synthesis. Cell *155*, 1075–1087.

Shannon, P., 2015. MotifDb: an annotated collection of protein-DNA binding sequence motifs. R package version 1.14.0.

Sherman, F. (2002). Getting started with yeast. Methods Enzymol. *350*, 3–41.

Uhler, J.P., Hertel, C., and Svejstrup, J.Q. (2007). A role for noncoding transcription in activation of the yeast PHO5 gene. Proc. Natl. Acad. Sci. USA *104*, 8011–8016.

Uprety, B., Kaja, A., Ferdoush, J., Sen, R., and Bhaumik, S.R. (2016). Regulation of antisense transcription by NuA4 histone acetyltransferase and other chromatin regulatory factors. Mol. Cell. Biol. *36*, 992–1006.

van Dijk, E.L., Chen, C.L., d'Aubenton-Carafa, Y., Gourvennec, S., Kwapisz, M., Roche, V., Bertrand, C., Silvain, M., Legoix-Né, P., Loeillet, S., et al. (2011). XUTs are a class of Xrn1-sensitive antisense regulatory non-coding RNA in yeast. Nature *475*, 114–117.

van Werven, F.J., Neuert, G., Hendrick, N., Lardenois, A., Buratowski, S., van Oudenaarden, A., Primig, M., and Amon, A. (2012). Transcription of two long noncoding RNAs mediates mating-type control of gametogenesis in budding yeast. Cell *150*, 1170–1181.

Venters, B.J., Wachi, S., Mavrich, T.N., Andersen, B.E., Jena, P., Sinnamon, A.J., Jain, P., Rolleri, N.S., Jiang, C., Hemeryck-Walsh, C., and Pugh, B.F. (2011). A comprehensive genomic binding map of gene and chromatin regulatory proteins in Saccharomyces. Mol. Cell *41*, 480–492.

Wyers, F., Rougemaille, M., Badis, G., Rousselle, J.-C., Dufour, M.-E., Boulay, J., Régnault, B., Devaux, F., Namane, A., Séraphin, B., et al. (2005). Cryptic pol II transcripts are degraded by a nuclear quality control pathway involving a new poly(A) polymerase. Cell *121*, 725–737.

Xu, Z., Wei, W., Gagneur, J., Perocchi, F., Clauder-Münster, S., Camblong, J., Guffanti, E., Stutz, F., Huber, W., and Steinmetz, L.M. (2009). Bidirectional promoters generate pervasive transcription in yeast. Nature *457*, 1033–1037.

Xu, Z., Wei, W., Gagneur, J., Clauder-Münster, S., Smolik, M., Huber, W., and Steinmetz, L.M. (2011). Antisense expression increases gene expression variability and locus interdependency. Mol. Syst. Biol. *7*, 468.

Cell Reports, Volume *15*

Supplemental Information

Protein Abundance Control

by Non-coding Antisense Transcription

Florian Huber, Daria Bunina, Ishaan Gupta, Anton Khmelinskii, Matthias Meurer, Patrick Theer, Lars M. Steinmetz, and Michael Knop

Figure S1

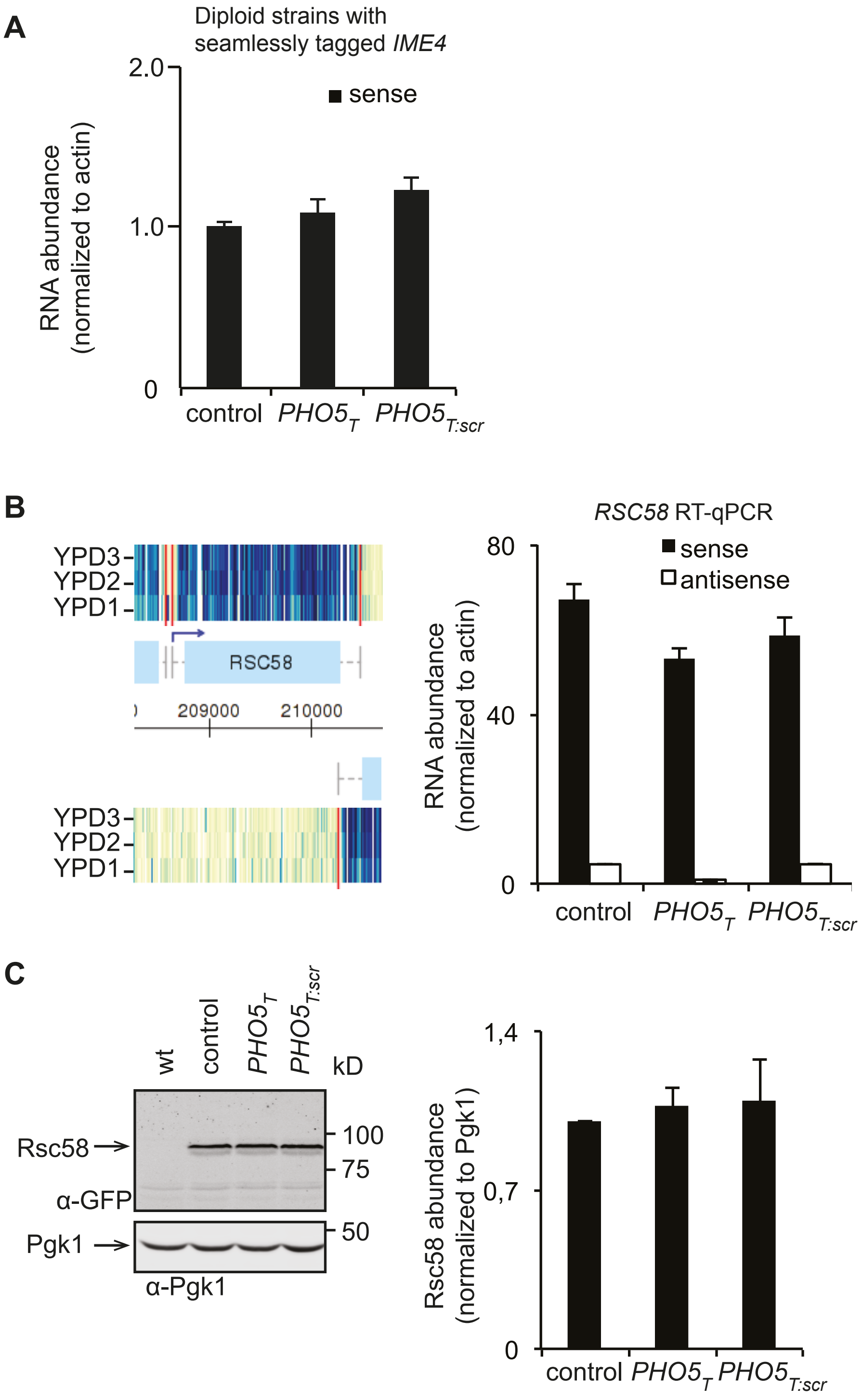
A
Diploid strains with
seamlessly tagged IME4
sense
RNA abundance
(normalized to actin)
2.0
1.0
0
control
PHO5T
PHO5T:scr
B
YPD3
YPD2
YPD1
RSC58
209000
210000
RSC58 RT-qPCR
sense
antisense
80
40
0
control
PHO5T
PHO5T:scr
C
wt
control
PHO5T
PHO5T:scr
kD
Rsc58
100
75
α-GFP
Pgk1
50
α-Pgk1
Rsc58 abundance
(normalized to Pgk1)
1,4
0,7
0
control
PHO5T
PHO5T:scr

Figure S1. qRT-PCRs and Western blots of diploid *IME4* and *RSC58*, related to Figure 1. (**A**) Sense RNA abundances in the diploid strains with *IME4*-sfGFP (or $PHO5_T$ or $PHO5_{T:scr}$) were analysed by qRT-PCR. Values are normalized to *ACT1* and the control (sfGFP only) was set to 1. Error bars indicate standard deviation of 3 replicates. Antisense transcripts were not detectable as they could not be distinguished from –RT controls. (**B**) Transcript abundances around the *RSC58* locus from the tiling array data are shown. Watson and Crick strands are at the top and the bottom, respectively. Three biological replicates in YPD medium are depicted. Darker blue color indicates higher hybridization signal. On the right RNA levels are shown, normalized to *ACT1* as determined by qRT-PCR for *RSC58*. Black: sense levels, gray: antisense levels. Error bars indicate standard deviation of 3 replicates. (**C**) Rsc58 protein abundance in the indicated constructs was determined by Western blotting with anti-GFP antibodies. The quantification of the normalized to Pgk1 band intensities for 3 replicates is shown on the right, error bars indicate standard deviations of 3 replicates.

Figure S2

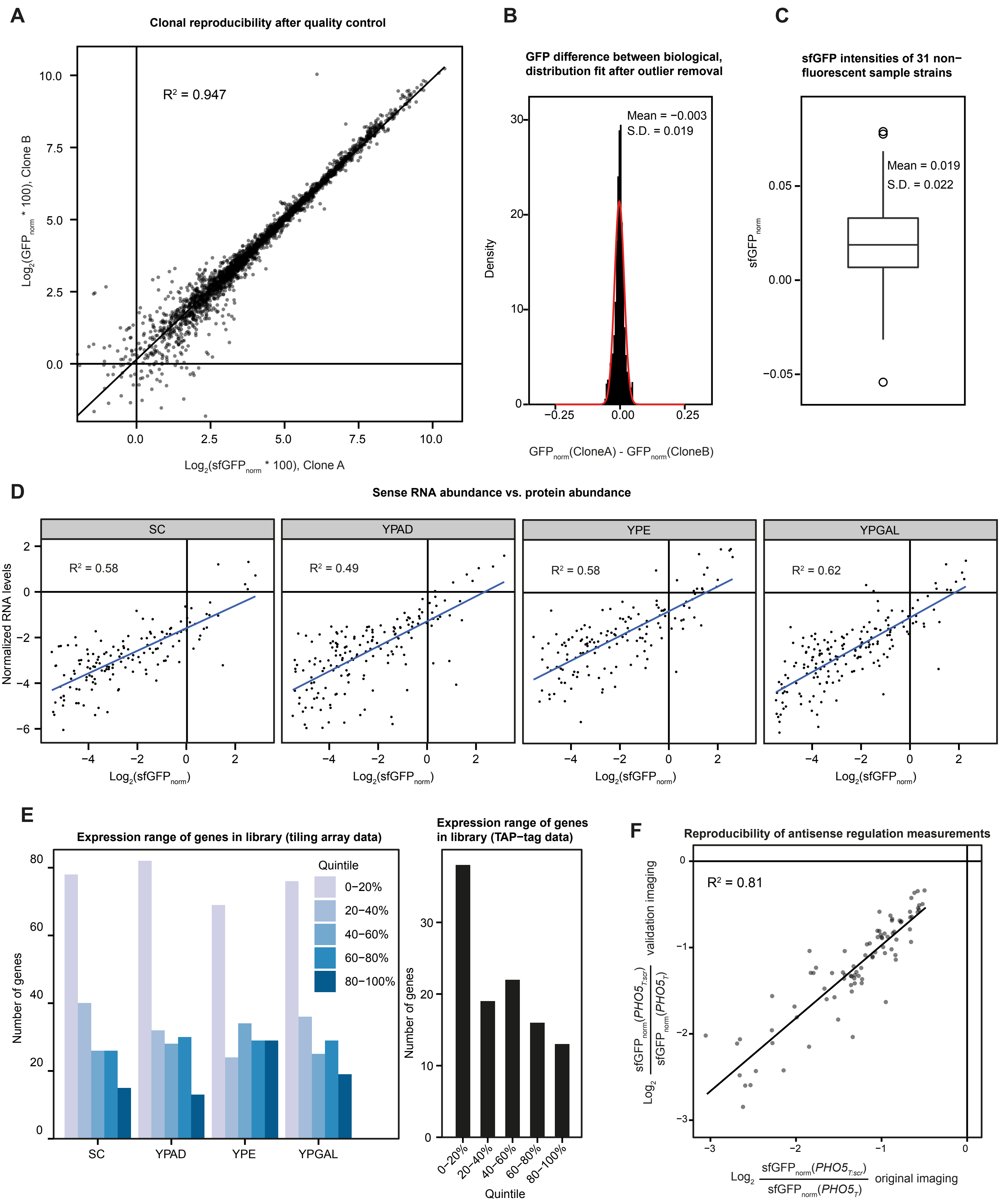
A
Clonal reproducibility after quality control
R² = 0.947
Log2(GFPnorm * 100), Clone B
Log2(sfGFPnorm * 100), Clone A
B
GFP difference between biological, distribution fit after outlier removal
Mean = −0.003
S.D. = 0.019
Density
GFPnorm(CloneA) - GFPnorm(CloneB)
C
sfGFP intensities of 31 non−fluorescent sample strains
Mean = 0.019
S.D. = 0.022
sfGFPnorm
D
Sense RNA abundance vs. protein abundance
SC
YPAD
YPE
YPGAL
R² = 0.58
R² = 0.49
R² = 0.58
R² = 0.62
Normalized RNA levels
Log2(sfGFPnorm)
E
Expression range of genes in library (tiling array data)
Quintile
0−20%
20−40%
40−60%
60−80%
80−100%
Number of genes
SC
YPAD
YPE
YPGAL
Expression range of genes in library (TAP−tag data)
Quintile
F
Reproducibility of antisense regulation measurements
R² = 0.81
Log2 sfGFPnorm(PHO5T:scr) / sfGFPnorm(PHO5T) validation imaging
Log2 sfGFPnorm(PHO5T:scr) / sfGFPnorm(PHO5T) original imaging

Figure S2. Quality control statistics of high throughput fluorescence microscopy and Northern blots, related to Figure 2. (**A**) Plot of the $Log_2(sfGFP_{norm} * 100)$ values of all pairs of biological replicates (clones) of the library after quality control with Pearson's R^2 indicated. (**B**) Distribution of the differences of normalized GFP intensities $sfGFP_{norm}$ of clones A and B before quality control but after outlier removal. The red line indicates a normal distribution with the mean and standard deviation values of the distribution as annotated in the plot. (**C**) Distribution of the normalized GFP intensities $sfGFP_{norm}$ (normalized to the fluorescence of the control cells without sfGFP) of 31 strains known to be non-fluorescent due to sfGFP frameshift mutations. The mean and standard deviation of this distribution was used to define thresholds for calling a gene expressed as explained in the Supplemental Experimental Procedures section below. (**D**) Normalized RNA levels from our tiling array data were plotted against protein expression values values obtained from microscopy. Straight lines are linear fits with the corresponding R^2 values indicated in the plot. (**E**) Left panel: using tiling array data, the number of genes in the antisense library falling in the respective quintile was plotted for every growth condition. Right panel: Using TAP-tag protein expression data obtained by (Ghaemmaghami et al., 2003) the number of genes in the antisense library falling in the respective quintile was plotted. Only values in YPAD were considered as the TAP-tag data were also obtained in that medium. The counts are lower than in the left panel because not for all genes in the library protein expression values were available. (**F**) 33 of the genes found to be regulated by antisense ('original imaging') were reimaged ('validation imaging'). For the conditions where those genes were found to be regulated in the original imaging, the ratios from the original imaging and the validation imaging were plotted against each other. The straight line is a linear fit with Pearson's R^2 shown in the plot.

Figure S3

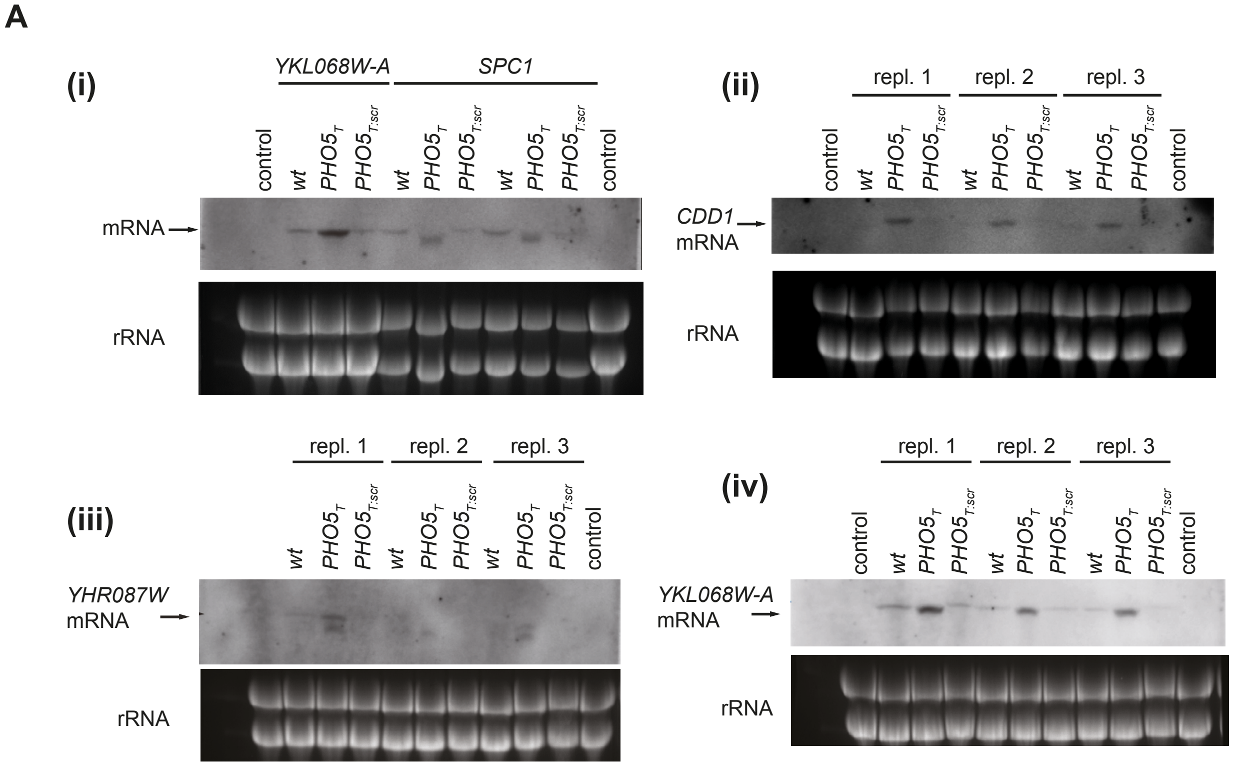
A
(i)
YKL068W-A
SPC1
control
wt
PHO5T
PHO5T:scr
mRNA
rRNA
(ii)
repl. 1
repl. 2
repl. 3
CDD1 mRNA
rRNA
(iii)
YHR087W mRNA
rRNA
(iv)
YKL068W-A mRNA
rRNA

Figure S3. Northern blots against mRNA of selected antisense-regulated genes, related to Figure 2. (**A**) Total RNA from up to three independent clones of each construct of the selected library genes was extracted and separated on the gels, followed by transfer to nylon membranes. The mRNAs of the indicated genes were detected using a DIG-labelled probe annealing in the sfGFP region of the transcripts. Ribosomal RNA staining by ethidium bromide was used as a loading control.

Figure S4

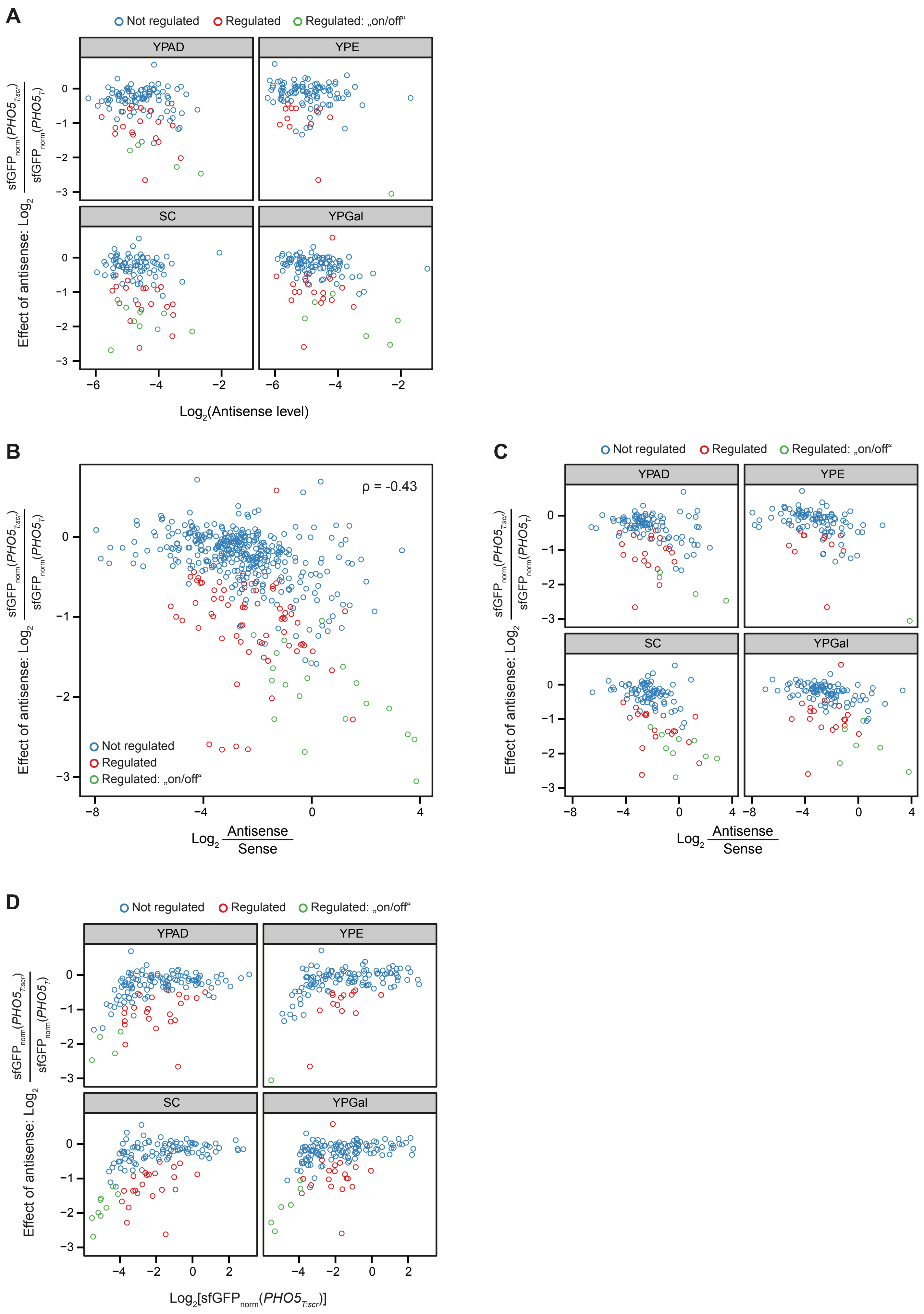
A
Not regulated
Regulated
Regulated: „on/off“
YPAD
YPE
SC
YPGal
Effect of antisense: $\log_2 \frac{sfGFP_{norm}(PHO5_{T:scr})}{sfGFP_{norm}(PHO5_T)}$
$\log_2$(Antisense level)
B
ρ = -0.43
$\log_2 \frac{Antisense}{Sense}$
C
D
$\log_2[sfGFP_{norm}(PHO5_{T:scr})]$

Figure S4. Correlation of regulation by antisense and antisense/protein levels, related to Figure 3. (**A**) The effect of antisense as measured by microscopy (log2-fold change of sfGFP levels between $PHO5_{T:scr}$ and $PHO5_{T}$) was plotted against the tiling array based antisense levels for all genes with annotated antisense. The different growth conditions are plotted in separate panels. (**B**). As in (**A**) but with the $\log_2$ of antisense/sense ratios as measured by tiling arrays plotted on the x-axis. (**C**) As in (B) but with different conditions plotted in separate panels. (**D**) As in (A) but with sfGFP intensities of the $PHO5_{T:scr}$ construct plotted on the x-axis.

Figure S5

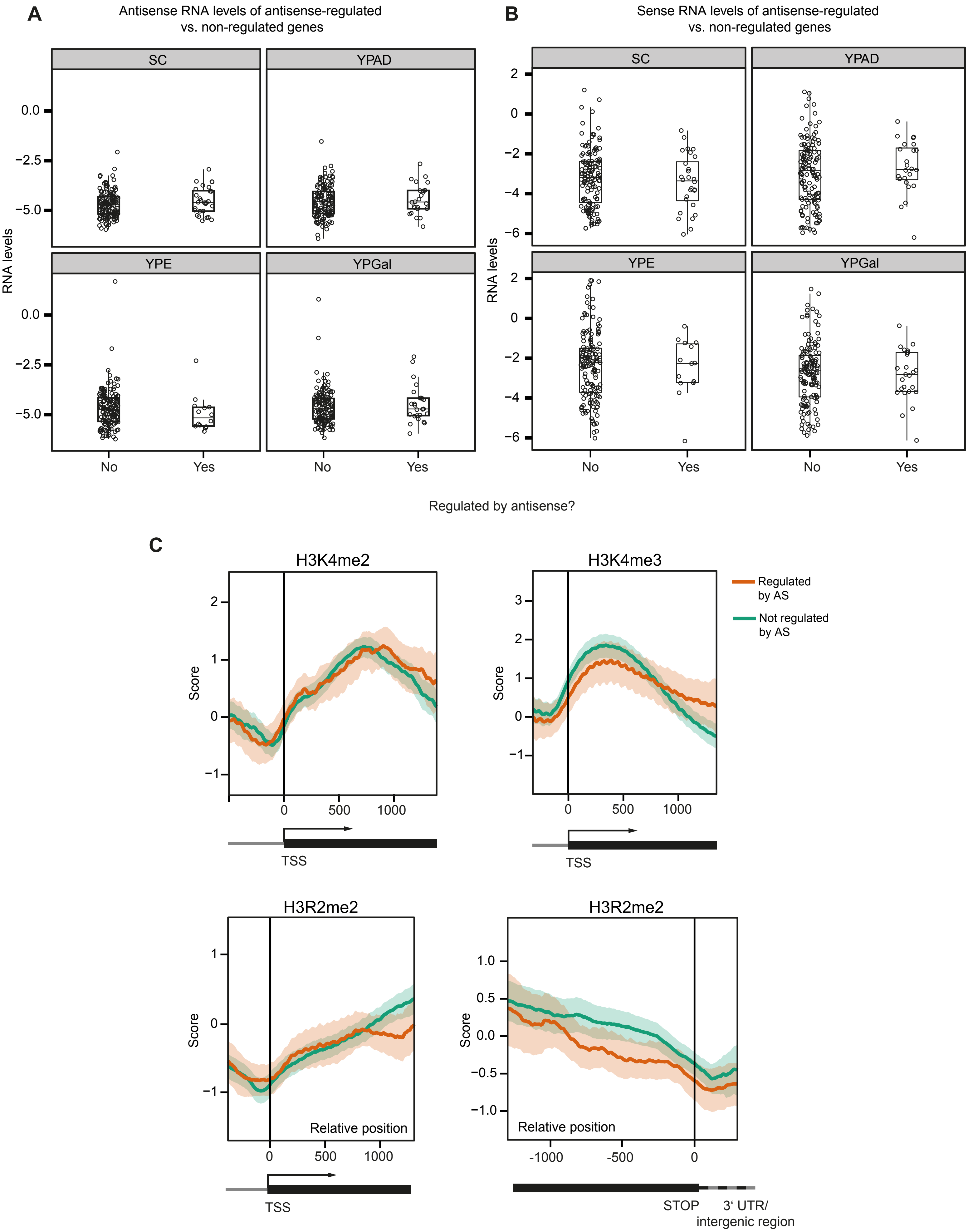
A
Antisense RNA levels of antisense-regulated
vs. non-regulated genes
SC
YPAD
YPE
YPGal
RNA levels
0.0
−2.5
−5.0
No
Yes
B
Sense RNA levels of antisense-regulated
vs. non-regulated genes
2
0
−2
−4
−6
Regulated by antisense?
C
H3K4me2
H3K4me3
Regulated
by AS
Not regulated
by AS
Score
TSS
H3R2me2
Relative position
−1000
−500
0
500
1000
STOP
3' UTR/
intergenic region

Figure S5. RNA levels of library genes and histone modification metagene plots, related to Figure 5. (**A**) Tiling array RNA expression levels of all the SUTs in the antisense library split by condition and depending on whether the corresponding sense gene was regulated by antisense in the respective condition. (**B**) Sense expression levels for the same genes shown in (A). (**C**) H3K4me2, H3K4me3 and H3R2me2 densities in antisense-regulated (red) vs. non-regulated (green) genes relative to either the TSS or the STOP codon as shown below the plots. Lines and ribbons indicate bootstrapping-based mean and 95% confidence interval estimates, respectively.

Figure S6

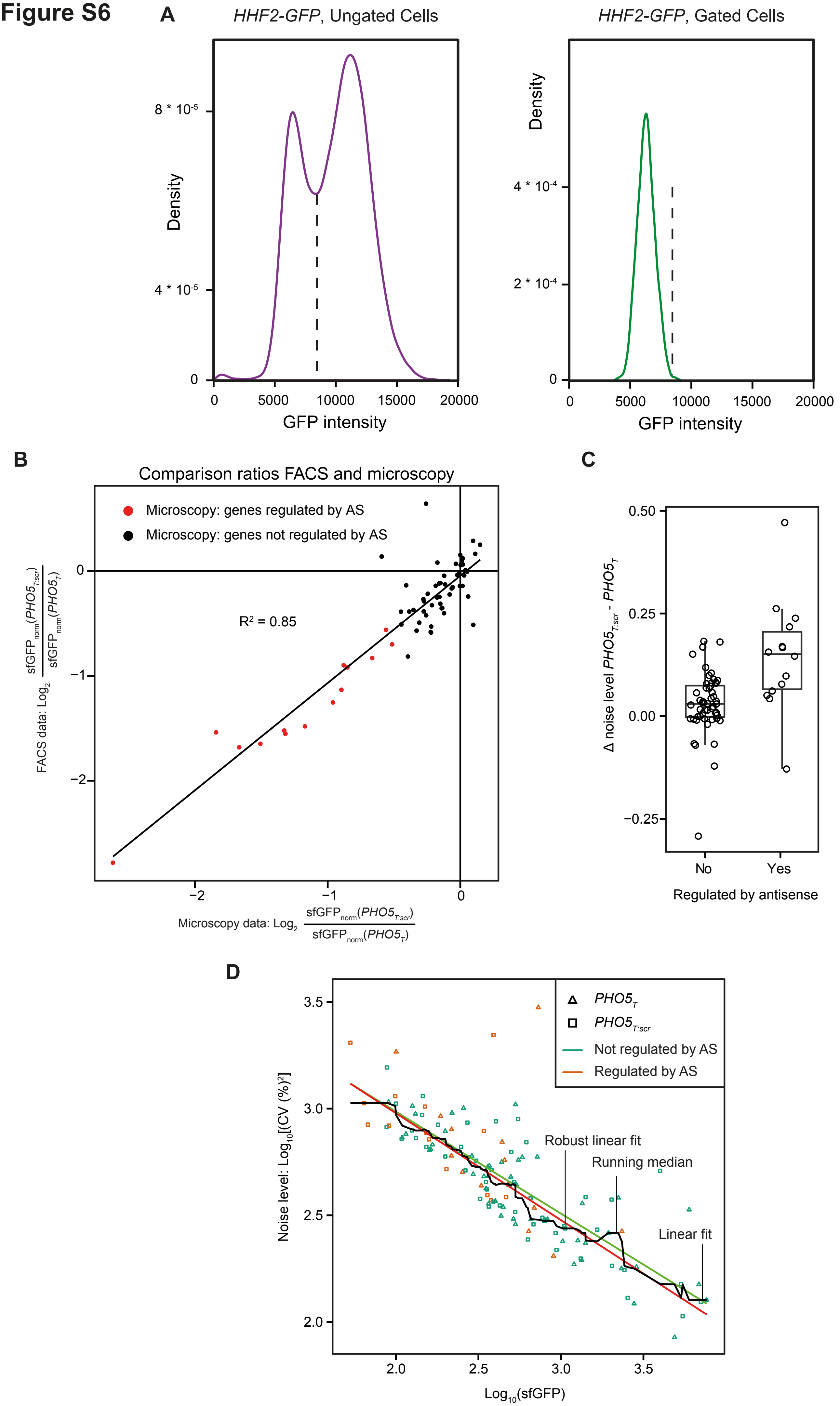

A
HHF2-GFP, Ungated Cells
HHF2-GFP, Gated Cells
Density
GFP intensity
B
Comparison ratios FACS and microscopy
Microscopy: genes regulated by AS
Microscopy: genes not regulated by AS
R² = 0.85
C
Δ noise level PHO5T:scr - PHO5T
No
Yes
Regulated by antisense
D
PHO5T
PHO5T:scr
Not regulated by AS
Regulated by AS
Robust linear fit
Running median
Linear fit
Noise level: Log10[(CV (%)²]
Log10(sfGFP)

Figure S6. Quality control of FACS results, related to Figure 6. (**A**) Distribution of GFP intensities of the histone gene *HHF2* before (left) and after (right) gating to obtain noise levels, as described in the Supplemental Experimental Procedures. (**B**) Log2 value sof the ratios of $PHO5_{T:scr}$ over $PHO5_T$ obtained either by FACS (y-axis) or microscopy (x-axis) were plotted against each other. Red color indicates that the gene was found to be regulated by antisense in microscopy. The straight line indicates a linear fit with (Pearson's $R^2 = 0.85$). The underlying data are from the SC growth condition. (C) A direct comparison of the gene-wise differences in noise levels (in units of $Log_{10}[CV\ (\%)^2]$, see Figure 6A) of $PHO5_{T:scr}$ minus $PHO5_T$ constructs. (**D**) In the original study by Churchman et al., a running median (black line) was used to fit the correlation between $Log_{10}(CV^2)$ and $Log_{10}(GFP)$. Due to the lower number of genes in our study, we found a robust linear fit to be less noisy (red line) and be less affected by outliers than a conventional linear fit (green line). Importantly, the outcome of the statistical test as shown in Figure 6B was not affected by the fitting procedure (data not shown).

Supplemental Tables

Table S1. Oligos used for antisense library construction, related to Figure 1.

This table is provided as a separate Excel file.

Table S2. Microscopy measurement values and quality control results of microscopy, related to Figure 2.

This table is provided as a separate Excel file.

Table S3. Quality control statistics, related to Figure 2.

Result of quality control	Number of wells	Percentage
**OK**	4217	93.5%
**Dead GFP**	212	4.7%
**QC fail**	83	1.8%
	**4512 total**<br>(4 conditions * 3 constructs * 2 clones * 188 genes)	100%

Table S4. Antisense regulated genes, related to Figure 2.

Growth condition	TSS overlap	No TSS overlap	No annotated antisense
YPAD	*CDD1, COX5B, FCY21, IME4, IML3, MCH5, MSS18, MTH1, OPT1, PTH1, SPC1, YHR022C-A, YHR087W, YKL068W-A, YNR004W, YPL162C, YTP1*	*CHS7, CTR1, PAN6, SHE9, SUR1, YHR112C*	*MRPL23, RCR2, YLR108C*
YPE	*CDD1, COX5B, IME4, NCA3, SPC1, YDR185C, YHR087W, YKL068W-A, YPL162C*	*ELO1, PAN6, SUR1, YGR203W*	*RCR2*
SC	*ARO10, CDD1, COX5B, FCY21, IME4, MBR1, MIG2, NCA3, OPT1, PTH1, SPC1, YDR185C, YGP1, YHR022C-A, YHR087W, YIL089W, YKL068W-A, YLR091W, YNR004W, YPL162C, YTP1*	*CTR1, MEP1, PAN6, SHE9, SUR1, YHR112C, YOR011W-A*	*MRPL23, RCR2, YLR108C*
YPGal	*CDD1, COX5B, FCY21, FET4, IME4, MCH5, MSS18, NCA3, PTH1, SPC1, YDR185C, YHR022C-A, YHR087W, YIL089W, YKL068W-A, YLR091W, YNR004W, YPL162C, YTP1*	*AMS1, JEN1, PAN6, SUR1, YHR112C*	*RCR2*

XY = Genes with possible switching behavior (PHO5$_{T:scr}$ values below expression threshold) in the respective condition.

Table S5. Tested histone modifications, related to Figure 5.

Modification	5' ends	3' ends	Reference
Nucleosome density	=	=	(Kirmizis et al., 2007)
H3K4me1	=	=	(Kirmizis et al., 2007)
H3K4me2	=	+	(Kirmizis et al., 2007)
H3K4me3	=	+	(Kirmizis et al., 2007)
H3K36me3	=	=	(Kirmizis et al., 2009)
H3K79me3	=	=	(Kirmizis et al., 2009)
H3K4ac	=	=	(Guillemette et al., 2011)
H3K9ac	=	=	(Pokholok et al., 2005)
H3K14ac	=	=	(Pokholok et al., 2005)
H4ac	=	=	(Pokholok et al., 2005)
H3R2me1	=	=	(Kirmizis et al., 2009)
H3R2me2	=	-	(Kirmizis et al., 2007)
Spt15	=	=	(Venters and Pugh, 2008)
Sua7	=	=	(Venters and Pugh, 2008)
Ser2-P	=	=	(Kim et al., 2010)
Ser5-P	=	=	(Kim et al., 2010)
Ser7-P	=	=	(Kim et al., 2010)

Supplemental Experimental Procedures

Yeast growth media. YPAD, YPGal and YPE were made by mixing 10 g/l peptone (BD), 10 g/l yeast extract (BD) and 0.05 g/l adenine (Sigma) and adding 2% of carbon source (glucose/galactose/ethanol, 20 g, 20 g and 20 ml, respectively). SC medium was made as previously described (Sherman, 2002).

Yeast strains. The background strain for construction of the antisense library was YMaM330: *MATα, can1Δ::STE2pr-his5 lyp1Δ::STE3pr-LEU2, his3Δ1 ura3Δ0 met15Δ0, leu2Δ0::GAL1pr-I-SCEI-natNT2* (Khmelinskii et al., 2011). The mCherry containing strain used for crossing was YDB1: *leu2Δ0 met15Δ0 ura3Δ0 his3Δ1::pGPD-mCherry*. The diploid *IME4* strains were obtained by crossing the looped-out library strains with YDB1, resulting in either *IME4-sfGFP/IME4*; *IME4-sfGFP-PHO5*$_T$*/IME4* or *IME4-sfGFP-PHO5*$_{T:scr}$*/ IME4.*

Plasmids. pFA6a is an *E. coli* plasmid with an *ampR* cassette (Wach et al., 1994). For seamless tagging with sfGFP plasmid pMaM175 was used (pFA6a-S3-sfGFP-SceIsite-S.Parad.Tcyc1-ScURA3-SceIsite-sfGFPΔN–S2). For tagging with *sfGFP-PHO5*$_T$ plasmid pMaM201 was used (pFA6a-S3-sfGFP-SceIsite-S.Parad.Tcyc1-ScURA3-SceIsite-sfGFPΔN–Tpho5-S2). For tagging with *PHO5*$_{T:scr}$ plasmid pMaM203 was used (pFA6a-S3-sfGFP-SceIsite-S.Parad.Tcyc1-ScURA3 -SceIsite-sfGFPΔN–Tpho5scr-S2). For integration of mCherry pDB2 (pRS303H with pGPD-mCherry) was used. All plasmids are available upon request.

Selection of genes for tagging. Genes with antisense SUT transcription were selected based on annotations from previous tiling array data (Xu et al., 2009). From all genes with antisense transcripts starting downstream of the STOP codon we randomly selected 81 ORFs whose antisense transcripts were annotated to overlap the TSS (including *IME4*) and 81 genes where this was not the case. As a third group, we added 26 ORFs without annotated antisense. Genes were C-terminally tagged using a cassette that allows for seamless tagging in the YMaM330 background (Khmelinskii et al., 2011).

Seamless tagging procedure. For C-terminal tagging of the selected genes 188 pairs of standard S2/S3 gene-specific oligonucleotide primers were synthesised, each of which contained 55 bp homology to the target locus to allow in-frame fusion of the sfGFP (Janke et al., 2004). Plasmid pMaM175 contained a seamless tagging cassette for tagging with sfGFP without any additional terminator sequence. The cassette was flanked by S2/S3 primer binding sites and contained two sfGFPs: one full sequence and an N-terminal truncation for subsequent homologous recombination. In between, there was a *URA3* marker surrounded by two I-SceI endonuclease sites. The order of genetic elements was thus pFA6a-S2site-sfGFP-SceI-URA3-SceI-ΔN-sfGFP-S3site. To create *PHO5*$_T$ and *PHO5*$_{T:scr}$ constructs, the *PHO5*$_T$ or *PHO5*$_{T:scr}$ sequence were inserted in antisense direction to the cassette described above between the ΔN-sfGFP and S3 site, resulting in plasmids pMaM201 and pMaM203. The haploid parent yeast strain YMaM330 was transformed with the PCR products generated from one of the cassettes using gene-specific primers. The clones were selected on SC -uracil medium (synthetic complete media with 2% glucose lacking uracil). Insertion of the cassette by homologous recombination was verified by PCR of samples from individual colonies using ORF specific forward primers and the generic reverse primer

sfGFPrev_alt2. Four positive clones of each strain were frozen and two were taken for further experiments. A list of all the ORFs with S2/S3 and validation primers can be found in Table S1.

To later distinguish sample from autofluorescent control cells in microscopy, the antisense library was crossed to YDB1, which contains a heterologous copy of mCherry, and subsequently selected for haploids containing both the tagged genes and mCherry using the SGA protocol (Tong and Boone, 2007). Two clones of the antisense library strains were then subjected to the loop out of the *URA3* marker.

Loop-out of the marker is based upon conditional expression of the I-SceI endonuclease on Galactose/Raffinose plates and subsequent double strand break repair via homologous recombination (Khmelinskii et al., PloS One 2011). After stimulating the loop-out with galactose cells with looped out marker are selected for by counterselection of the *URA3* marker on SC medium containing 5-fluoroотic acid. The final seamlessly tagged strains were used in all further experiments.

Fluorescence microscopy.

Cells were inoculated to 96-well plates in their respective media and grown to saturation over night at 30 °C. For growth in YPE the cells were additionally pre-cultured in YPE after saturation in YPAD. Saturated cultures were diluted to OD_{600} = 0.025 into fresh medium and mixed 1:1 with sfGFP-negative control cells for background subtraction and signal normalization on a well-by-well basis. Populations were distinguished by using an mCherry cassette contained in the antisense library. Cultures were grown to mid log phase for 7-8 hours. 70 µl of the cultures were transferred to 96-well PCR plates and fixed with 70 µl of freshly prepared 8% paraformaldehyde in 1x PBS for 5 minutes. The cells were washed 5 times using 1x PBS and then resuspended in 120 µl of 1x PBS. 20 µl of the cell suspension were used for microscopy in 384-well glass-bottom microscopy plates (Matriplate, GE Healthcare) pretreated with concanavalin A (100 µg/ml, Sigma). Imaging was performed on a Nikon Ti-E wide field epifluorescence screening microscope with a 60x ApoTIRF oil-immersed objective (1.49 NA, Nikon), an LED light engine (SpectraX, Lumencor), a 2048x2048 pixel (6.5 µm) sCMOS camera (Flash4, Hamamatsu) and an autofocus system (Perfect Focus System, Nikon). Nine images for each of mCherry, sfGFP and brightfield channels were recorded per position using three different exposure times for sfGFP. Wells to correct for shading artifacts were set up for every plate.

Image postprocessing and cell segmentation. All fluorescence images were flat-field corrected to correct for uneven illumination across the field of view. Before flat-field correction, the camera offset, which is introduced by manufacturers to prevent negative values, was subtracted from all images (including reference images). Next, flat-field correcation was done on a plate-by-plate basis by dividing every image by a reference image derived from a well containing a diluted recombinant mCherry-sfGFP fusion protein. To obtain the reference image, all images from the corresponding well were median projected, smoothed by a combination of median, mean and Gaussian filters and divided by the image median. Next, cells were segmented by processing out of focus brightfield images using a modified version of the pipeline published by Buggenthin and colleagues (Buggenthin et al., 2013). The obtained images were further processed using custom ImageJ scripts to generate cell masks of the appropriate size. These scripts minimized the number of masks derived from out of focus cells and excluded all masks with an area <5 µm^2 or >15 µm^2 or with a circularity <0.85. Sample cells and autofluorescent control cells were co-cultured and distinguished by a heterologous mCherry cassette in the

sample cells. Masks were therefore split into a sample and an autofluorescent control cell population using image-based thresholding in the mCherry channel. Next, masks were applied to the flat-field corrected GFP images resulting for every cell in mean fluorescence intensity values and morphological parameters.

Calculation of normalized sfGFP values and determining a gene expression threshold. Background subtraction and normalization were done for every well independently to control for well-to-well and plate-to-plate variability. For every well, background subtraction was done by subtracting the median sfGFP intensity of the autofluorescent background cell population from the sfGFP intensity of every sample cell. The resulting background subtracted sfGFP intensities of the sample cells were then normalized by dividing by the median sfGFP intensity of the autofluorescent background cell population. Thus, the normalized sfGFP intensity for every sample cell is calculated by:

$$sfGFP_{norm} = \frac{sfGFP - median(sfGFP_{bg})}{median(sfGFP_{bg})}$$

where $sfGFP_{norm}$ is the normalized sfGFP intensity of a sample cell and *sfGFP* denotes its raw sfGFP signal intensity. median($sfGFP_{bg}$) is the median sfGFP signal intensity of the autofluorescent background cell population. The obtained median of the normalized $sfGFP_{norm}$ intensities of the sample cell population represented our preliminary gene expression values.

To find out which strains expressed their tagged genes above background, we determined the normalized sfGFP intensity of 31 non-functional sfGFP strains in the library where both visual inspection and Sanger sequencing confirmed the absence of functional sfGFP as compared to the second clone of the strain. We considered the preliminary expression values of those strains as our background (Figure S2C). The resulting mean (0.019) was subtracted from preliminary expression values to obtain final gene expression values normalized to the autofluorescence of non-fluorescent cells on a well-by-well basis (termed $\text{sfGFP}_{\text{norm}}$). Subsequently, we defined a strain to be expressed above background if the final gene expression value was higher than 3 standard deviations of the $\text{sfGFP}_{\text{norm}}$ values of the non-functional sfGFP strains (resulting in a threshold of 0.065, see Figure S2C). To prevent ratios from approaching infinity, gene expression values found to be lower than 1 standard deviation of the non-functional strains were set to 1 standard deviation of those strains.

Quality control of microscopy data. First, uniformity of growth between sample and autofluorescent control cells was determined by checking the differences in the median areas between the two populations. We ensured that these values were centered around 0 with only minor deviations (< 0.5 μm^2, data not shown). Three sfGFP exposure times, 50 ms, 300 ms, and 1000 ms were applied during imaging. Suitable exposure times were chosen independently for each well by taking the highest possible exposure time where images were not overexposed. Only two genes, *FBA1* and *PDC1* were overexposed at all exposure times and excluded from further analysis. Proper separation of mCherry negative and mCherry positive populations (see Image postprocessing and cell segmentation section above) was checked by requiring that median mCherry intensities differed by at least four-fold between the two populations and by performing occasional visual inspection. Wells failing to meet these criteria were excluded from further analysis. Wells were also excluded from the analysis if the number of cells in any of the two populations was less than 100 as we found medians obtained from such population sizes to be

less well reproducible (data not shown). However, cell numbers were in general much higher with the median cell number per well being 950 for every population and the lower decile at 357 cells.

Next, we identified non-fluorescent strains among the expressed strains. These are a result of sequence mutations introduced during the library construction which are not detectable by colony PCR and typically result in one of the two biological replicates (clones) lacking fluorescence. To identify such cases, we first determined how much clones differed on average by fitting a normal distribution to the distribution of interclonal gene expression differences (Figure S2B). If two clones differed more than expected from this distribution and were more than 50% different from each other, the clone with a weaker expression was flagged. If a flagged clone was found to be consistently not expressed under any of the growth conditions, it was considered bad and excluded from further analysis, otherwise it was excluded only in the condition were quality control was not passed. A table containing the quality control results for the whole microscopy run can be found in Table S2. Summary statistics of quality control and reproducibility of our dataset after quality control can be found in Table S3. Subsequent Sanger sequencing revealed that >90% of the excluded clones showed frameshift mutations in the regions responsible for homologous recombination of the tagging cassette (data not shown).

Determining antisense-regulated genes. To determine genes regulated by antisense in a particular growth condition, we calculated p-values for the log2-fold changes of the ratios between the $PHO5_{T:scr}$ and the $PHO5_{T}$ strains using a linear modelling approach as implemented in the limma package of Bioconductor (Ritchie et al., 2015; Smyth, 2004). Multiple testing correction was done using the method by Benjamini and Hochberg (Benjamini and Hochberg, 1995). Hits were required to have p value of <0.01 and to differ more than 4 standard deviations of the distribution of interclonal differences (Figure S2B). Genes were $PHO5_{T:scr}$ differed more than 50% from the control (sfGFP only) strains were excluded prior to this analysis because we reasoned that in those cases the introduction of additional nucleotides at the 3' end of the gene led to a major impairment of native gene function. We also did not consider genes that under the respective condition fell below the expression in both the $PHO5_{T}$ and the $PHO5_{T:scr}$ constructs.

Determining condition-specific regulation. All genes that were regulated in at least one condition were selected as candidates for condition-specific regulation. Cases where $\log_2(PHO5_{T:scr}/PHO5_{T})$ differed significantly were determined using limma, setting the significance cutoff to p = 0.01 and requiring that $\log_2$-fold changes differ by at least 1 and/or approach 0 in one of the tested conditions. To call condition-specific regulation, we furthermore stipulated that (i) both $PHO5_{T:scr}$ and $PHO5_{T}$ be above the expression threshold because in those cases obtained $\log_2(PHO5_{T:scr}/PHO5_{T})$ are better reproducible and (ii) that the gene be significantly regulated (see above) in not all of the conditions.

Metagene analyses. For NET-seq metagene analysis (Figure 4C) the genome coordinates of 750 bp regions were retrieved for every gene, ranging either from -300 bp to +450 bp of the transcript start sites (TSSs) or from -450 bp to + 300 bp of the STOP codon (TSS and STOP being coordinates 0). TSS positions were based on tiling array data and are available upon request. Next, NET-seq read numbers at the respective regions were retrieved for every gene using the dataset from Churchman and colleagues (Churchman and Weissman, 2011). Genes were then first grouped into antisense-regulated and non-regulated genes and those groups were further

split up by their original classification, i.e. depending on whether antisense transcripts were annotated and if so, whether they overlapped the TSS or not. Only genes tested for regulation (see section "Determining antisense-regulated genes" above) were considered and antisense regulation was based on data obtained in YPAD as this corresponds to the condition used by Churchman et al. NET-seq read numbers were then averaged for every gene in bins of 10 nucleotides and the median read numbers of those bins determined for every group. Finally, the resulting traces were smoothed using a running mean of 10 bins. For metagene traces of histone modifications (Figures 5A and B and Figure S5C) the approach was similar but with changes in parameter settings as follows: Genes that did not have an annotated antisense transcript were not considered. The bin size was set to 1 nucleotide and the window size was set to 150 bins. Mean values and confidence intervals at a 95% level were calculated using nonparametric bootstrapping as implemented in the R Hmisc package, version 3.17-0. For a description of the data sources used see the paragraph "Data sources" below.

Data sources. Transcript boundaries and ncRNA annotations were based on tiling array data sets obtained in our laboratory as described above. Gene sequences, gene models and annotations were derived from Bioconductor packages using genome version sacCer3 from UCSC (2011). NET-seq data were obtained from (Churchman and Weissman, 2011). ChIP-chip data on H3K4me3 and H3R2me2 were obtained from (Kirmizis et al., 2007). ChIP-chip data on H3K36me3, H3K79me3, H3R2me1 data were obtained from (Kirmizis et al., 2009). ChIP-chip on H3K4ac was obtained from (Guillemette et al., 2011). Data on H3K9ac, H3K14ac and H4ac was obtained from (Pokholok et al., 2005). Data on Sua7 and Spt15 occupancy was obtained from (Venters and Pugh, 2008). PolII CTD phosphorylation patterns were obtained from (Kim et al., 2010). Binding sites of RNA-binding proteins were obtained from (Hogan et al., 2008). For identification of known DNA sequence motifs, the Bioconductor MotifDb package was used (Shannon, 2015). The motif database was cleared of redundant entries by preferring ScerTF over JASPAR over UniPROBE derived motifs. To discover *de novo* motifs that are enriched in hits vs. non-hits or vice versa, the tool DREME from the MEME suite, version 4.10.2 (Bailey et al., 2009).

Flow cytometry: data processing and noise estimation. Strains with intact sfGFP fluorescence (using the information obtained by microscopy) were obtained from our antisense library by streaking to single colonies. Two independent colonies (clones) were then grown in 96-well plates (8 plates in total). We recorded 100,000 events per well of mid-log phase cells growing in SC using a flow cytometer equipped with a high throughput stage (BD FACSCanto RUO HTS). Subsequently, single (G1) cells were selected by plotting the side scatter width of all cells and selecting for the smaller population (Matthias Meurer, personal communication). Next, a circular gate was defined as described in (Newman et al., 2006) that results in a homogeneous yeast cell population where the influence of extrinsic noise is minimized (leaving roughly 5000 – 10000 cells per well using a radius of 5000). Briefly, the medians of forward and side scatter widths are taken as the center of a circular gate and the radius of this gate is reduced in small steps. Initially, the coefficient of variation (CV) drops but at some point stabilizes at a certain value that is largely dominated by intrinsic noise (Newman et al., 2006). sfGFP values were normalized plate-wise by dividing by fluorescent beads added to all the wells of the first column of every plate. A threshold for expression was determined by measuring a non-fluorescent strain for every plate and calculating a 99.9% confidence interval. Strains with sfGFP values below this threshold were

not considered. For reproducibility, we stipulated that sfGFP and CV values be within a 15% range of each other. We validated the strategy by comparing the CV values obtained from a set of reference strains used in Newman et al., 2006. Similar values were obtained (data not shown). As an additional control, the GFP intensity profile of a *HHF2-GFP* strain resulted after gating in a uniform population of G1 cells (Figure S6A). To account for the dependence of the CV values on protein abundance (Figure 6A) we calculated a robust linear fit to the data, using the implementation as in the MASS package in R, version 7.3-45. We found this to be a better fit than the approach using a running median in Newman's study. Subsequent calculation of the residuals to that fit enable the assignment of noise levels while accounting for changes in protein abundance. We then subtracted the residual of $PHO5_{T:scr}$ from the one in $PHO5_T$ for every gene separately and compared the distributions of antisense-regulated genes to non-regulated ones as previously determined by microscopy in SC medium (Figure 6B).

Statistical tests. Unless otherwise specified, two distributions were compared as follows: First, the underlying data was tested for normality using the Shapiro-Wilk test. Next, in the case of normal distribution a Student's t-test was performed, otherwise a Wilcoxon's rank sum test (all tests run as implemented in R, package stats, version 3.2.2). Multiple testing corrections were performed if adequate, using the method by Benjamini and Hochberg (Benjamini and Hochberg, 1995).

Northern blotting. Total yeast RNA was extracted from mid-log phase cultures as described in (Collart and Oliviero, 2001). Northern blotting of total RNA was performed as described (Luke et al., 2008) with some modifications. Briefly, 20 µg of total RNA were separated by electrophoresis on a formaldehyde agarose 1.2% gel. RNA was transferred to IMMOBILON NY+ charged nylon membrane (Millipore). Blots were blocked in UltraHyb hybridization buffer (Ambion) for 1h at 42 °C and hybridized for 14-16h with DIG-labelled probes (DIG-labeling kit, Roche). Blots were washed (2x SSC with 0.1% SDS; 0.5x SSC with 0.1% SDS; 0.2x SSC with 0.1% SDS), then blocked and incubated with anti-DIG antibodies (Roche). The signals were detected using CDP-star reagent (Roche) and a chemiluminescent imager. Ribosomal RNA stained with ethidium bromide was used as a loading control.

RT-qPCR primer sequences.
sfGFP fwd: TGTTAGAGGTGAGGGCGAAG
sfGFP rev: TACTAGGGTTGGCCAAGGAA
actin fwd: TGATGACTTGACCATCTGGAAGTTCGTAGG
actin rev: GGACTTCGAACAAGAAATGCAAACCGC
sfGFPrev_alt2: CTCTTCACCCTTGGACATAGC

Supplemental References

Benjamini, Y., Hochberg, Y., 1995. Controlling the False Discovery Rate: A Practical and Powerful Approach to Multiple Testing. J. R. Stat. Soc. Ser. B Methodol. 57, 289–300.

Buggenthin, F., Marr, C., Schwarzfischer, M., Hoppe, P.S., Hilsenbeck, O., Schroeder, T., Theis, F.J., 2013. An automatic method for robust and fast cell detection in bright field images from high-throughput microscopy. BMC Bioinformatics 14, 297.

Guillemette, B., Drogaris, P., Lin, H.-H.S., Armstrong, H., Hiragami-Hamada, K., Imhof, A., Bonneil, É., Thibault, P., Verreault, A., Festenstein, R.J., 2011. H3 Lysine 4 Is Acetylated at Active Gene Promoters and Is Regulated by H3 Lysine 4 Methylation. PLoS Genet 7, e1001354. doi:10.1371/journal.pgen.1001354

Janke, C., Magiera, M.M., Rathfelder, N., Taxis, C., Reber, S., Maekawa, H., Moreno-Borchart, A., Doenges, G., Schwob, E., Schiebel, E., Knop, M., 2004. A versatile toolbox for PCR-based tagging of yeast genes: new fluorescent proteins, more markers and promoter substitution cassettes. Yeast 21, 947–962. doi:10.1002/yea.1142

Kim, H., Erickson, B., Luo, W., Seward, D., Graber, J.H., Pollock, D.D., Megee, P.C., Bentley, D.L., 2010. Gene-specific RNA polymerase II phosphorylation and the CTD code. Nat. Struct. Mol. Biol. 17, 1279–1286. doi:10.1038/nsmb.1913

Kirmizis, A., Santos-Rosa, H., Penkett, C.J., Singer, M.A., Green, R.D., Kouzarides, T., 2009. Distinct transcriptional outputs associated with mono- and dimethylated histone H3 arginine 2. Nat. Struct. Mol. Biol. 16, 449–451. doi:10.1038/nsmb.1569

Luke, B., Panza, A., Redon, S., Iglesias, N., Li, Z., Lingner, J., 2008. The Rat1p 5′ to 3′ Exonuclease Degrades Telomeric Repeat-Containing RNA and Promotes Telomere Elongation in Saccharomyces cerevisiae. Mol. Cell 32, 465–477. doi:10.1016/j.molcel.2008.10.019

Ritchie, M.E., Phipson, B., Wu, D., Hu, Y., Law, C.W., Shi, W., Smyth, G.K., 2015. limma powers differential expression analyses for RNA-sequencing and microarray studies. Nucleic Acids Res. doi:10.1093/nar/gkv007

Smyth, G.K., 2004. Linear Models and Empirical Bayes Methods for Assessing Differential Expression in Microarray Experiments. Stat. Appl. Genet. Mol. Biol. 3, 1–25.

Tong, A.H.Y., Boone, C., 2007. 16 High-Throughput Strain Construction and Systematic Synthetic Lethal Screening in, in: Methods in Microbiology. Elsevier, pp. 369–707.

Venters, B.J., Pugh, B.F., 2008. A canonical promoter organization of the transcription machinery and its regulators in the Saccharomyces genome. Genome Res. 19, 360–371. doi:10.1101/gr.084970.108

Wach, A., Brachat, A., Pöhlmann, R., Philippsen, P., 1994. New heterologous modules for classical or PCR-based gene disruptions in Saccharomyces cerevisiae. Yeast 10, 1793–1808. doi:10.1002/yea.320101310
